# Highly Active Cu_14_ Cluster Precisely Activates Autophagy Inhibitor to Amplify Cuproptosis Immunotherapy

**DOI:** 10.1002/advs.202517021

**Published:** 2026-03-26

**Authors:** Qiu‐Xu Zang, Wei‐Tong Chen, Yu‐Ying Shen, Zhao‐Yang Wang, Kai Li, Xueli Zhao, Yanjuan Sang, Xiaoyuan Chen, Shuang‐Quan Zang

**Affiliations:** ^1^ State Key Laboratory of Metabolic Dysregulation & Prevention and Treatment of Esophageal Cancer Henan International Joint Laboratory of Tumor Theranostic Cluster Materials College of Chemistry Zhengzhou University Zhengzhou Henan P. R. China; ^2^ Tianjian Laboratory of Advanced Biomedical Sciences Institute of Advanced Biomedical Sciences Zhengzhou University Zhengzhou Henan P. R. China; ^3^ Shandong Provincial Key Laboratory of Precision Oncology Shandong Cancer Hospital and Institute Shandong First Medical University and Shandong Academy of Medical Sciences Jinan Shandong P. R. China

**Keywords:** autophagy suppression, copper cluster, cuproptosis, ICD, prodrug activation

## Abstract

Transition metal‐mediated cleavage mechanisms have emerged as an effective means to mitigate the off‐target toxicity of conventional therapeutic agents. However, the utilization of non‐essential metal catalysts and suboptimal catalytic efficiency often compromises their therapeutic efficacy. Given the critical role of copper in biological processes and the high catalytic activities of atomically precise copper clusters rich in Cu^+^, herein, a Cu_14_ cluster‐mediated cleavage reaction is first employed for synergistic cuproptosis‐associated immunogenic cell death (ICD) induction with localized autophagy suppression to intensify immunotherapy effects. To endow homologous targeting capability, the Cu_14_ cluster is camouflaged with cancer cell membrane, obtaining Cu_14_@CM. Upon tumor accumulation, the pH‐sensitive Cu_14_@CM releases copper ions to trigger cuproptosis through lipoylated protein oligomerization and iron‐sulfur cluster protein disruption, further releasing damage‐associated molecular patterns (DAMPs) and generating robust ICD. Simultaneously, the cluster catalyzes bond cleavage reactions to produce autophagy inhibitors, blocking cytoprotective autophagy to amplify DAMPs exposure. This dual‐action strategy increases dendritic cell maturation, and elevates tumor‐infiltrating cytotoxic T lymphocytes, thereby reinforcing the antitumor immune response.

## Graphical abstract

An atomically precise Cu_14_ cluster‐mediated bond scission reaction pioneers the amplification of the antitumor immune response via synergetic cuproptosis‐associated immunogenic cell death induction and in situ autophagy blockade.

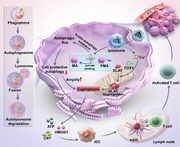

## 1 Introduction

Immunotherapy, as a promising clinical treatment tactic, has made revolutionary advances in the scope of defeating cancer [1, 2, 3]. Particularly, immunogenic cell death (ICD), characterized by the release of damage‐associated molecular patterns (DAMPs), is a prominent means to create an immunogenic tumor microenvironment and further activate immune response [4, 5, 6, 7]. Despite this, its efficacy is frequently impeded by protective autophagy [8, 9], which diminishes the expression of surface antigens on carcinoma cells, hinders immune cell recognition and facilitates tumor immune evasion [10, 11]. Although some autophagy inhibitors have been used to boost immunotherapy performance [12, 13, 14], their application is often compromised by non‐specific delivery and systemic toxicity [15, 16, 17]. Recently, transition metal‐mediated catalytic reactions, particularly bond‐scission processes, have emerged as powerful tools for localized drug synthesis across therapeutic contexts [18, 19, 20]. Their predominance stems from superior adaptability in prodrug reactivation and precise regulatory capacity. Nevertheless, contemporary catalytic platforms remain fundamentally constrained by the introduction of non‐essential metal elements and suboptimal catalytic efficiency, compromising therapeutic efficacy [21, 22]. Therefore, it is desired to develop a novel and highly efficient transition metal catalytic bond‐breaking reaction system for the in situ synthesis of autophagy inhibitors to enhance ICD‐related immunotherapy.

Atomically precise coinage metal clusters, an emerging class of materials, have recently garnered significant attention across the fields of devices, catalysis, and biomedicine [23, 24, 25]. They are typically composed of a specific number of metal atoms at the core, with surface‐organic ligands attached. The accurate structures and molecular purity make them ideal models for revealing the structure‐activity relationship and identifying catalytic active sites [26, 27]. More importantly, metal clusters can stabilize multiple metal ions even in unstable oxidation states and exhibit multiple active sites as well as high surface‐to‐volume ratios, offering enhanced catalytic performance and great advantages in organic reactions, including coupling and cleavage reactions [28]. Particularly for copper clusters, in addition to the aforementioned characteristics, they can function as an efficient reservoir of copper ions and possess the potential to induce cuproptosis in tumor cells. Cuproptosis is a newly identified Cu^+^‐dependent mechanism of programmed cell death characterized by aggregation of lipoylated mitochondrial proteins and the subsequent loss of iron‐sulfur (Fe‐S) cluster proteins, thereby causing protein toxicity stress and ultimately leading to cell death [29, 30]. Recent studies have proved that cuproptosis is associated with ICD and can activate the immune response [31, 32, 33, 34]. Building on these points, we envision that the targeted delivery of copper clusters with a high proportion of Cu^+^ to tumor sites for precise in situ activation of autophagy inhibitors may provide a safe and effective approach to promote cuproptosis‐related ICD and further reinforce immunotherapy.

**SCHEME 1:**
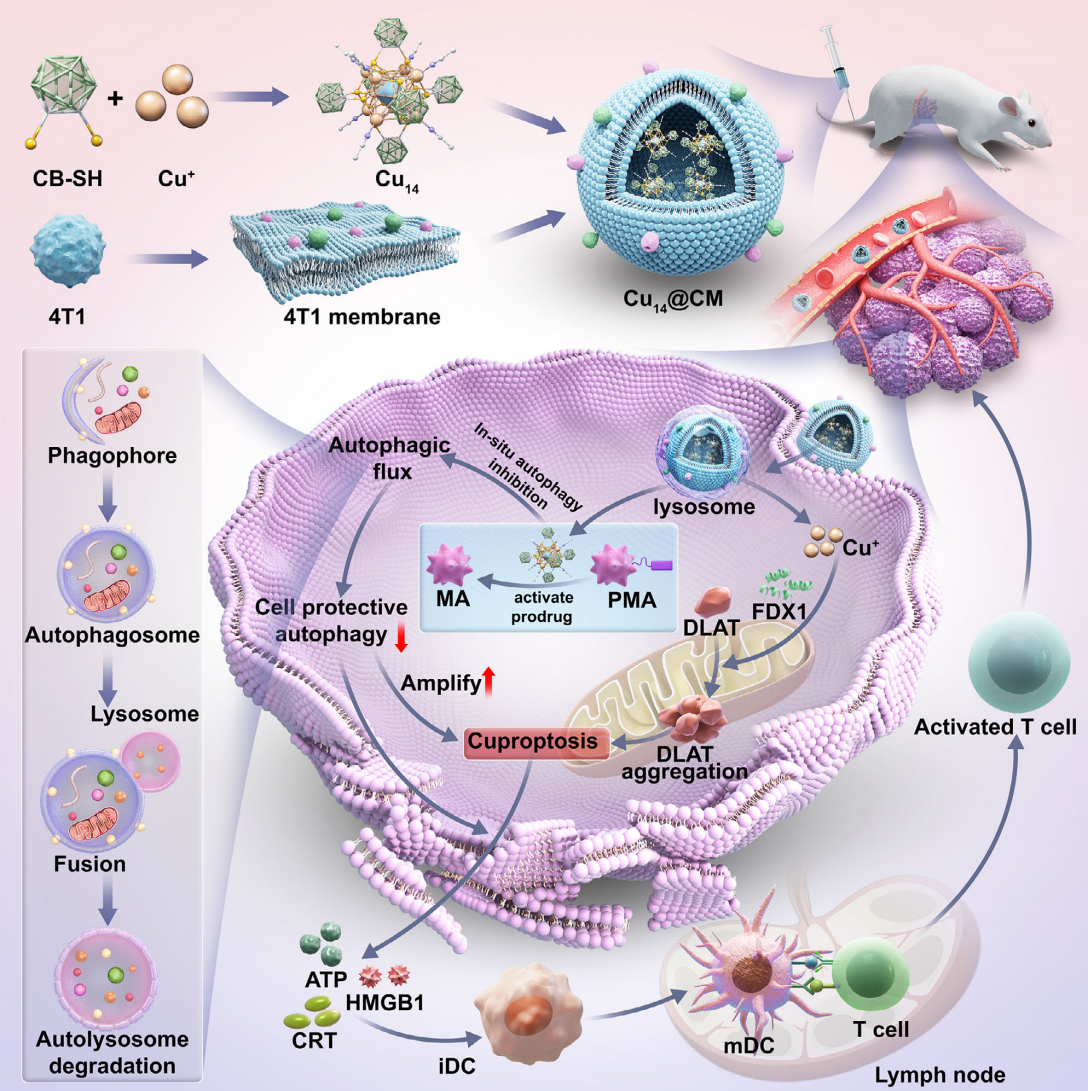
Schematic illustration of the synthesis of Cu_14_@CM and its application in the in situ activation of an autophagy inhibitor to amplify cuproptosis‐induced immunotherapy. CB‐SH, 1,2‐dithiol‐*o*‐carborane; MA, 3‐methyladenine; PMA, pro‐3‐methyladenine; DLAT, dihydrolipoamide *S*‐acetyltransferase; FDX1, ferredoxin 1; ATP, adenosine triphosphate; CRT, calreticulin; HMGB1, high mobility group box 1; iDC, immature dendritic cell; mDC, mature dendritic cell.

Herein, for the first time, a copper cluster‐mediated cleavage reaction was developed to in situ activate an autophagy inhibitor prodrug for intensive cuproptosis‐immunotherapy (Scheme 1). In detail, the Cu_14_(C_2_B_10_H_10_S_2_)_6_(CH_3_CN)_8_ (abbreviated as Cu_14_) cluster with a high proportion of Cu^+^ was first prepared and post‐camouflaged [35, 36] with cancer cell membranes to obtain Cu_14_@CM. The cell membrane coating endowed the homologous targeting capability to the Cu_14_ cluster, which made it effectively accumulate at the tumor sites. After being ingested by tumor cells, on one hand, Cu_14_@CM gradually released a large number of copper ions, leading to the oligomerization of lipoylated proteins, depletion of Fe‐S cluster proteins, and release of DAMPs, ultimately giving rise to cuproptosis‐mediated ICD. On the other hand, Cu_14_@CM with high catalytic activity could in situ precisely activate the prodrug of autophagy inhibitor pro‐3‐methyladenine (PMA), counteracting the self‐defense mechanisms of tumors. This process facilitated DAMPs exposure, enhanced tumor immunogenicity, and promoted dendritic cells (DCs) maturation and effector T cell activation, ultimately amplifying the antitumor immune response. In general, this work provides a new tactic for prodrug activation and will promote the application of copper clusters in drug activation and cuproptosis.

## 2 Results and Discussion

### 2.1 Structure and Characterization of Cu_14_ Cluster

**FIGURE 1:**
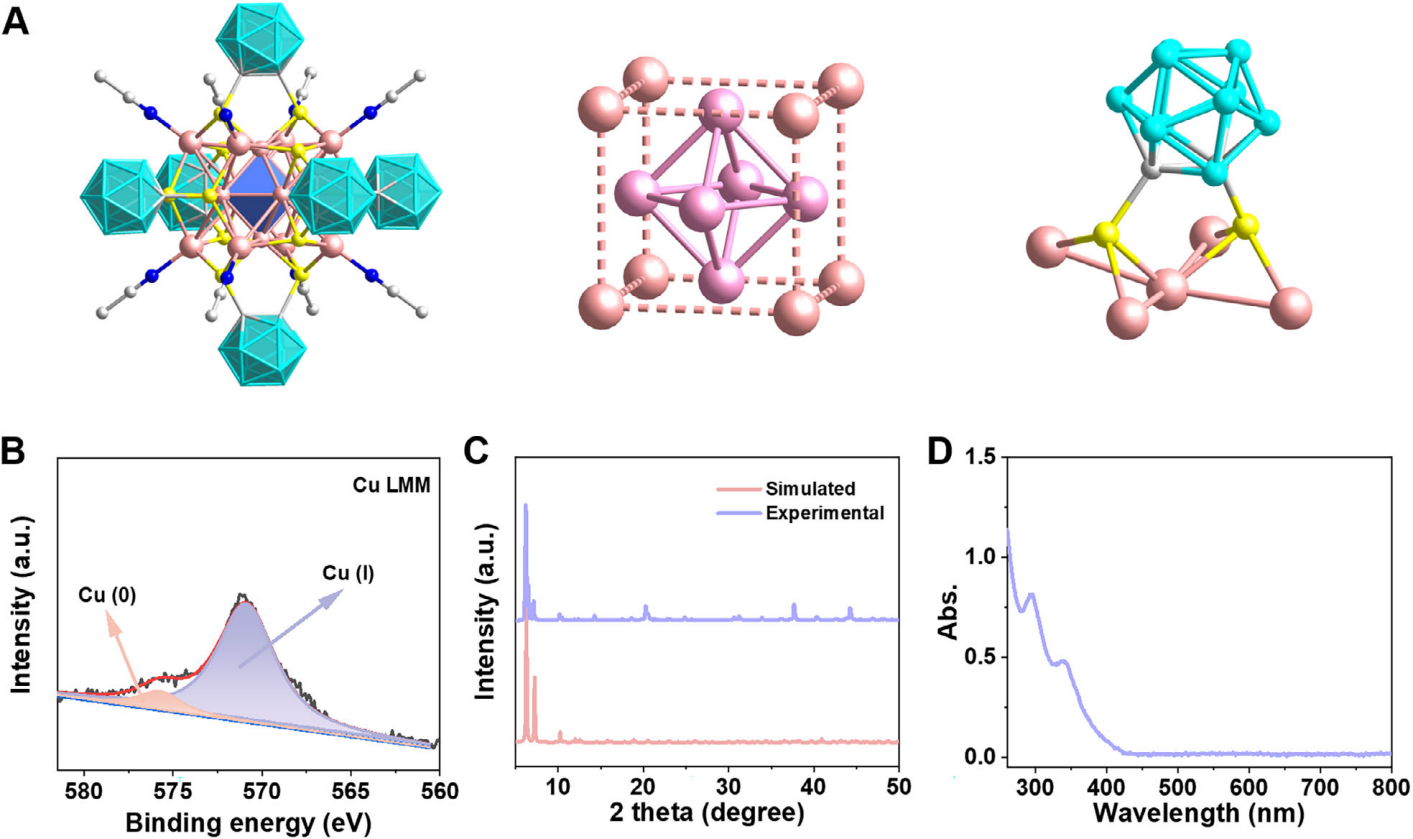
(A) The complete geometric configuration and hierarchical structural analysis of the Cu_14_ cluster. (B) Auger electron spectrum, (C) PXRD patterns and (D) UV–vis absorption spectrum of Cu_14_ cluster. Color code: pinkish‐orange or pink, Cu; yellow, S; blue, N; turquoise, B; gray, C. For clarity, all H atoms are omitted.

Primarily, the Cu_14_ cluster was synthesized according to the literature with slight modifications [37]. Briefly, Cu(CF_3_COO)_2_ was reacted with 1,2‐dithiol‐*o*‐carborane (CB‐SH) in a mixture of tetrahydrofuran and acetonitrile under continuous stirring. Then, the supernatant was allowed to slowly evaporate at room temperature, yielding light green block crystals. Single‐crystal X‐ray diffraction analysis demonstrates that the cluster features a Cu_14_ core, consisting of a regular octahedral (oct) Cu_6_ unit and a Cu_8_ cubic framework (cub) encapsulating Cu_6_ unit (Figure 1A). Six CB‐SH ligands and eight acetonitrile molecules are respectively arranged around the periphery of the Cu_14_ metal core. Each CB‐SH ligand connects one Cu_oct_ atom and four Cu_cub_ atoms via *µ_5_‐η^1^, η^1^, η^2^, η^1^, η^1^* bonding mode. Acetonitrile molecules are respectively coordinated to eight Cu_cub_ atoms. Overall, the molecular formula of the cluster was determined to Cu_14_(C_2_B_10_H_10_S_2_)_6_(CH_3_CN)_8_ (named as Cu_14_). The oxidation state of copper is predominantly Cu^+^, along with a portion of Cu^0^, which is consistent with the result of Figure 1B. Then, the phase purity was characterized by powder X‐ray diffraction (PXRD, Figure 1C). The transmission electron microscopy (TEM) image of the Cu_14_ cluster (Figure S1) reveals an average size of approximately 1.33 nm. The UV–Vis absorption spectrum showed characteristic absorption peaks at 295 and 340 nm (Figure 1D). The fluorescence spectrum revealed that Cu_14_ exhibited red emission with characteristic peaks at 606 and 646 nm (Figure S2).

### 2.2 The Prodrug‐Activating Ability of the Cu_14_ Cluster

**FIGURE 2:**
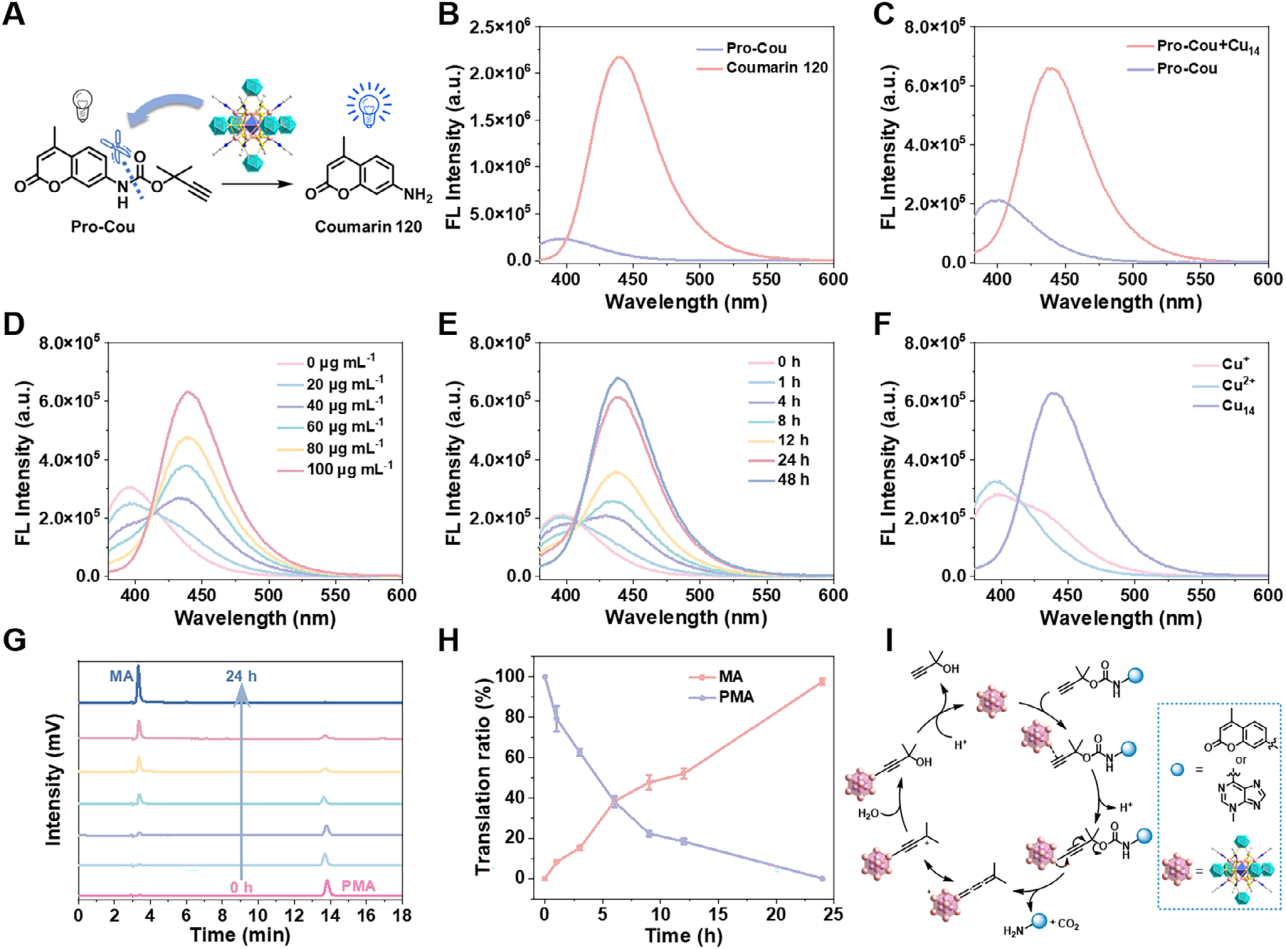
(A) The schematic illustration of the catalytic bond cleavage reaction mediated by Cu_14_. (B) The fluorescence spectra of Pro‐Cou and Coumarin 120. (C) The fluorescence spectra of Pro‐Cou and Pro‐Cou+Cu_14_ after incubating at 37°C for 48 h. The fluorescence spectra of Pro‐Cou at (D) different concentrations of Cu_14_ and (E) different reaction times. (F) The fluorescence spectra of the catalytic bond cleavage reaction mediated by CuBr, CuSO_4_ and Cu_14_. (G) The HPLC analysis of reaction process catalyzed by Cu_14_ at different time. (H) The translation ratio of PMA to MA, as determined by HPLC analysis. (I) The schematic illustration showed the mechanism of the catalytic bond cleavage reaction mediated by Cu_14_. Data are presented as mean ± s.d. from 3 independent biological replicates.

Subsequently, the ability of Cu_14_ cluster to catalyze the activation of prodrugs was assessed. Primarily, the cleavage of 2‐methylbut‐3‐yn‐2‐yl (4‐methyl‐2‐oxo‐2H‐chromen‐7‐yl)carbamate (Pro‐Cou) was first studied (Figure 2A), which can be transformed into strongly fluorescent coumarin 120 under copper‐based catalysts [38]. Figure 2B showed that coumarin 120 had a stronger‐intensity emission peak at 440 nm compared to Pro‐Cou. Leveraging this property, Pro‐Cou was synthesized according to the literature [38] (Figure S3) and further incubated with Cu_14_ cluster to investigate its bond cleavage capability. As evidenced in Figure 2C‐E, Cu_14_ displayed potent catalytic activity and revealed concentration‐ and time‐dependent characteristics. Notably, in contrast to catalytically inert CuSO_4_ and weakly active CuBr, Cu_14_ cluster demonstrated superior catalytic performance attributable to its multiple active sites and synergistic electron transfer within the metallic core (Figure 2F). Based on this results, the activation of Cu_14_ cluster toward prodrug, PMA, was evaluated. The high‐performance liquid chromatography (HPLC) was utilized to monitor the production of MA. The converted ratio was determined through the HPLC standard curves of PMA and MA (Figure S4). Figure 2G showed that Cu_14_ could gradually catalyze the transformation of PMA to MA over time. And the catalytic reaction could complete within 24 h (Figure 2H). A possible catalytic reaction mechanism was proposed in Figure 2I [39]. The C─O bond cleavage process was initiated by the coordination of the alkyne moiety to the core copper atom. Subsequently, a cluster acetylide species was formed. This was followed by an intramolecular electron transfer, leading to the cleavage of C─O bond. Meanwhile, the cluster‐stabilized propargylic cation intermediate generated and was further rapidly captured by water to form propargyl alcohol. Then, the propargyl alcohol dissociated to complete the catalytic cycle, while the cluster proceeded to the next catalytic round.

Furthermore, the ability of Cu_14_ to catalyze the generation of •OH from H_2_O_2_ was investigated as well, which may potentially assist in tumor treatment [40, 41]. The 3,3',5,5'‐tetramethylbenzidine (TMB) was selected as the indicator. As illustrated in Figure S5, the absorbance of the solution comprising TMB, H_2_O_2_ and Cu_14_ was markedly higher than that of the other groups, thereby signifying its notable catalytic capability. In addition, its catalytic activity was found to be closely associated with the concentrations of H_2_O_2_, TMB, and Cu_14_, and the pH level of the reaction medium. The oxidation reaction rate of TMB was progressively accelerated with the increasing concentrations of H_2_O_2_ (Figure S6A), TMB (Figure S6B) and cluster (Figure S6C). However, with the increase of pH, the catalytic activity of the cluster first increased and then decreased. Notably, the Cu_14_ cluster exhibited excellent catalytic activity within the pH range of 4–6, enabling it to effectively catalyze the generation of reactive oxygen species (ROS) in the tumor microenvironment and lysosomal acidic environments (Figure S6D). The remarkable catalytic capabilities of the Cu_14_ cluster in both prodrug activation and ROS generation highlight its potential as a promising therapeutic agent for cancer treatment.

### 2.3 Intracellular Cu_14_@CM‐Mediated Catalytic Bond Cleavage

**FIGURE 3:**
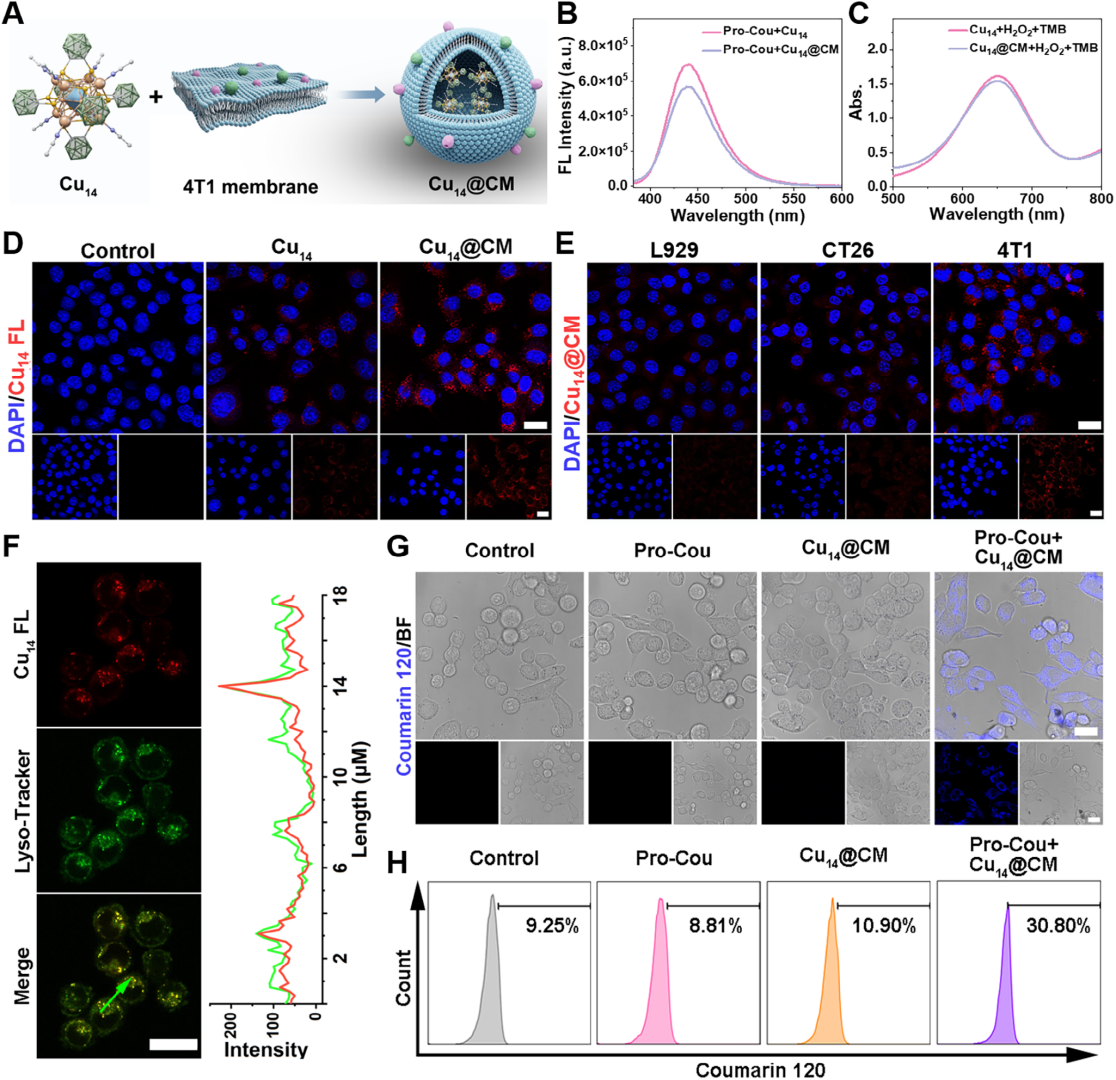
(A) Schematic illustration of the synthesis of Cu_14_@CM. (B) The fluorescence spectra of Pro‐Cou incubated with Cu_14_ or Cu_14_@CM for 48 h. (C) UV–vis absorbance spectra of TMB under different treatment conditions. (D) Comparative cellular uptake of Cu_14_ and Cu_14_@CM in 4T1 cells. Scale bars are 25 µm. (E) The CLSM images of different cells after treating with Cu_14_@CM. Scale bars, 25 µm. (F) Co‐localization analysis of Cu_14_@CM in 4T1 cells with corresponding light signal profiles (green arrows). Lysosomes were stained with Lyso‐Tracker Green. Scale bar is 20 µm. (G) The bond cleavage reaction of Pro‐Cou mediated by Cu_14_@CM in 4T1 cells. Scale bars are 25 µm. (H) Flow cytometric analysis of 4T1 cells incubated with distinct experimental groups.

Before applying in cancer therapy, the mouse breast cancer cells (4T1) membrane (CM) was collected and coated on the Cu_14_ cluster to improve the targeting ability toward the homologous tumor (Figure 3A). The TEM image (Figure S7), elemental mapping (Figure S8), and sodium dodecyl sulfate‐polyacrylamide gel electrophoresis (Figure S9) together confirmed the successful coating of Cu_14_ with the cancer cell membrane. Moreover, the appearance of stretching vibration of N‐H and P = O in Fourier Transform Infrared (Figure S10) spectrum and P 2p signal peak in X‐ray photoelectron spectroscopy (Figure S11A,B) spectra demonstrated the successful preparation of Cu_14_@CM as well. After coating with CM, the surface zeta potential of Cu_14_ decreased to −38.8 mV (Figure S12), which had negligible impact on its subsequent utilization. The hydrodynamic diameter of Cu_14_@CM was determined to be approximately 125.6 nm (Figure S13). Importantly, CM coating exerted no significant effect on the oxidation state of Cu, suggesting the maintaining of the catalytic activity of Cu_14_ cluster (Figure S11C,D). Figure 3B,C provided the direct evidence for this conjecture. Cu_14_@CM demonstrated catalytic efficacy comparable to the Cu_14_ cluster in both prodrug activation and ROS generation, thereby ensuring therapeutic effectiveness.

It is well recognized that the stability is critical for the in vivo application of nanomedicines. Therefore, the stability of Cu_14_@CM was investigated via UV–vis absorbance spectroscopy and the release of Cu ion. Figure S14 demonstrated that the CM camouflage did not obviously affected the UV–vis absorption characteristic peak of Cu_14_ cluster. Even more surprisingly, in comparison with Cu_14_, the UV–vis absorption spectra of Cu_14_@CM exhibited no apparent alteration in phosphate buffer saline (PBS, pH 7.4), indicating higher stability (Figures S15 and S16). Furthermore, no change in the copper valence state was observed within 24 h in PBS solution (Figures S17 and S18), further substantiating its structural and chemical stability. In contrast, the absorption spectra of Cu_14_@CM decreased continuously over a 24 h period in PBS at pH 4, indicating that Cu_14_@CM had insufficient stability under acidic conditions (Figure S19). Further quantitative analysis revealed that the cumulative Cu ion release reached 82.09% at pH 4 within 24 h (Figure S20). This acid response release behavior indicates its potential application in triggering cuproptosis [42]. Additionally, the catalytic performance of Cu_14_@CM in PBS at pH 4 was assessed as well. Figure S21 showed the enhanced fluorescence intensity at 440 nm with the extension of time, confirming the feasibility of intracellular prodrug activation by Cu_14_@CM after lysosomal escape.

Based on the excellent catalytic performance and acid‐responsive copper ion release behavior of Cu_14_@CM, its abilities to activate the prodrug and induce cell cuproptosis were further examined at the cellular level. First, its cellular uptake behavior was studied utilizing the red luminescence characteristic of Cu_14_ cluster. Figure S22 showed a time‐dependent increase in red fluorescence intensity, indicating effective accumulation of Cu_14_@CM in 4T1 cells. Compared with Cu_14_, cells treated with Cu_14_@CM exhibited enhanced fluorescence intensity (Figure 3D), validating the auxiliary role in intracellular delivery of biomimetic coating. Notably, the pronounced fluorescence selectivity of Cu_14_@CM in 4T1 cells over L929 (mouse fibroblast cells) and CT26 (mouse colorectal carcinoma cells) cells (Figure 3E) revealed its homologous targeting specificity, implying potential biocompatibility advantages for normal tissues. After being internalized, the subcellular distribution was further observed by CLSM. Figure 3F displayed that Cu_14_@CM had a high degree of co‐localization with lysosomes, with a corresponding Pearson's coefficient of 0.7931. The high overlap of the red curve with the green curve on the path of the arrow in the Figure 3F also demonstrated the precise localization of Cu_14_@CM in lysosomes. This subcellular distribution feature was highly favorable for the Cu ion release, thereby triggering cell cuproptosis.

Afterwards, the catalytic bond cleavage reaction of Pro‐Cou mediated by Cu_14_@CM in 4T1 cells was evaluated. Fluorescence spectroscopic analysis revealed that coumarin 120 exhibited intense fluorescence emission at 450 nm when excited at 405 nm, whereas Pro‐Cou demonstrated negligible emission under identical conditions (Figure S23). This marked fluorescence difference prompted the selection of 405 nm as the excitation wavelength for subsequent intracellular catalytic assays. As illustrated in Figure 3G, cells treated with Cu_14_@CM exhibited a significant increase in blue fluorescence intensity compared to the other groups, indicating that Cu_14_@CM effectively catalyzed bond cleavage in the intracellular environment. Furthermore, flow cytometry analysis likewise confirmed the occurrence of intracellular cleavage mediated by Cu_14_@CM (Figure 3H). These results proved the feasibility of intracellular prodrug activation induced by Cu_14_@CM, which would further inhibited cell autophagy and enhanced immunogenic cuproptosis.

### 2.4 The Anticancer Performance of Cu_14_@CM In Vitro

**FIGURE 4:**
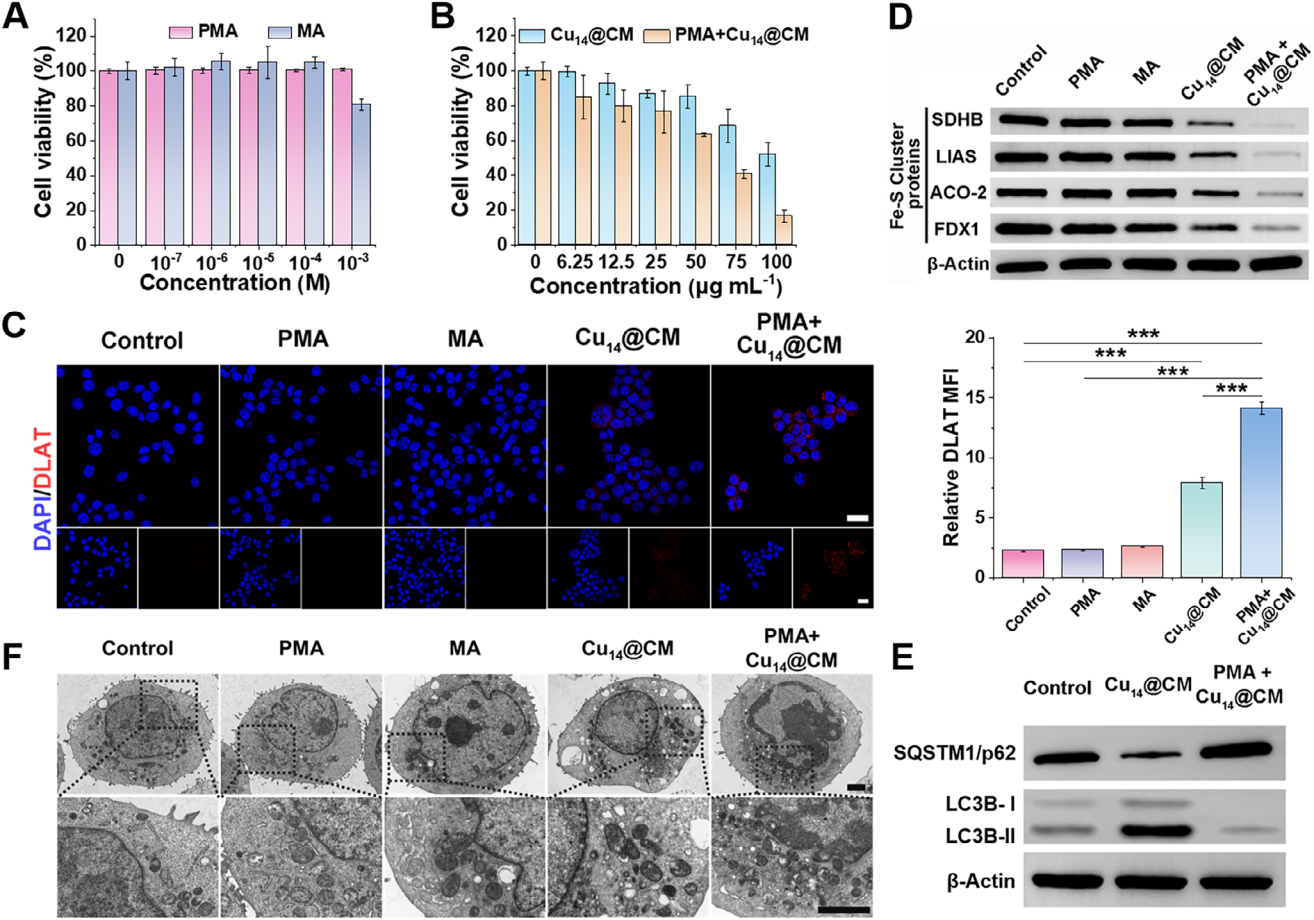
(A) Assessment of 4T1 cell viability following treatment with PMA and MA. (B) Evaluation of cytotoxic effects induced by Cu_14_@CM and Cu_14_@CM+PMA in 4T1 cells. (C) CLSM images of DLAT and corresponding quantitative analysis in 4T1 cells after different treatments. Scale bars, 25 µm. (D) Western blot analysis of Fe‐S cluster proteins in 4T1 cells under different treatment conditions. (E) Western blot results of the autophagy‐related proteins in 4T1 cells across treatment groups. (F) Bio‐TEM micrographs of 4T1 cells after different treatments. Scale bars, 2 µm. Data are presented as mean ± s.d. from 3 independent biological replicates. Two‐tailed Student's t‐test were used for comparisons between two independent groups (*p < 0.05, **p < 0.01, ***p < 0.001).

Then the cytotoxicity of Cu_14_@CM against 4T1 cells in both the presence and absence of PMA was evaluated. As depicted in Figure 4A, PMA alone exerted no significant effect on 4T1 cell viability, while MA showed slightly cytotoxicity with the increase of concentration. In contrast to the modest cytotoxicity induced by free Cu_14_ (Figure S24A), Cu_14_@CM treatment led to a dramatic decrease in cell viability (Figure 4B), and this cytotoxic effect was further potentiated when PMA was co‐administered. The cell live/dead staining (Figure S25) and flow cytometry analysis (Figure S26) provided the same results. The negligible cytotoxicity induced by CB‐SH (Figure S27) suggested that the observed cytotoxicity of Cu_14_@CM could be attributed to intracellular Cu ion release, which induced cellular cuproptosis. When combined with PMA treatment, Cu_14_@CM may trigger intracellular prodrug activation to generate MA, a classical autophagy inhibitor that disrupts autophagosome formation through inhibition of the Class III PI3K/Beclin‐1 complex [15, 43]. This suppression of cellular protective autophagy potentiates cuproptosis‐mediated therapeutic efficacy. Figure S24B also suggest that the superior tumor cell killing efficacy of PMA+Cu_14_@CM may not be a simple additive effect of individual components, but rather a synergistic reaction resulting from Cu_14_‐triggered PMA activation. To verify these hypotheses, we examined intracellular PMA activation and the expression patterns of cuproptosis‐related biomarkers and autophagy markers across different treatment groups. Figure S28 showed the appearance of the MA signal peak of in cellular extracts following treatment with PMA+Cu_14_@CM, confirming successful intracellular prodrug activation. Afterthat, the intracellular level of dihydrolipoamide *S*‐acetyltransferase (DLAT), one of the lipoylated mitochondrial proteins, and Fe‐S cluster related protein was evaluated by immunofluorescence staining and western blot analysis. The abnormal aggregation of lipoylated proteins within mitochondria and destabilization of Fe‐S cluster proteins will lead to proteotoxic stress and ultimately cause cuproptosis [44, 45]. The negligible fluorescence intensity in untreated, PMA‐treated, and MA‐treated cell groups indicated the minimal DLAT oligomerization (Figure 4C). In contrast, Cu_14_@CM or PMA+Cu_14_@CM treated cell groups displayed the high fluorescence intensity, suggesting the substantial oligomer formation. Furthermore, the expression levels of Fe‐S cluster proteins exhibited a marked reduction in Cu_14_@CM and PMA+Cu_14_@CM groups (Figure 4D). Notably, the PMA+Cu_14_@CM group exhibited significantly enhanced DLAT oligomerization and depletion of Fe‐S cluster proteins compared to Cu_14_@CM alone, a phenomenon likely linked to suppressed autophagy. Then autophagy‐related proteins, such as autophagy substrate sequestosome 1 (SQSTM1, referred to as p62) and microtubule‐associated protein 1 light chain 3 (LC3), were measured. The conversion of LC3 from the cytosolic form LC3‐I to the activated membrane‐bound form LC3‐II represented the formation of autophagosomes and the initiation of autophagy. Furthermore, the p62 facilitated the recruitment of ubiquitinated cargo to autolysosomes for degradation, a process that was critical for the assessment of autophagic flux [43, 46]. Figure S29 showed that MA exhibited an autophagy‐inhibitory effect, as evidenced by decreased LC3‐II expression and overexpression of p62. However, PMA displayed levels of LC3‐II and p62 similar to those of the control group, indicating that PMA failed to effectively inhibit autophagy. These results also demonstrated the suppression of MA's efficacy after modifying by alkyne. After treating with Cu_14_@CM, the expression of LC3‐II increased while that of p62 declined (Figure 4E), indicating the induction of autophagy. Furthermore, Bio‐TEM results also proved that Cu_14_@CM‐treated cells exhibited a significant increase in autophagic vacuoles, validating enhanced autophagic flux within the cells (Figure 4F). Meanwhile, influenced by protective autophagy, the mitochondrial damage caused by Cu_14_@CM is limited, which in turn affects its cuproptosis therapeutic effect. PMA coadministration induced autophagic vacuoles depletion coupled with pathological mitochondrial accumulation. This pharmacodynamic synergy originates from Cu_14_@CM‐mediated catalysis of PMA into MA, effectively suppressing copper‐ion‐activated cytoprotective autophagy to potentiate cuproptosis efficacy. In addition, the Cu_14_@CM could mediate the generation of intracellular ROS (Figures S30 and S31), further intensifying the mitochondrial dysfunction through reducing mitochondrial membrane potential (Figure S32). To further elucidate cytotoxic mechanism of PMA+Cu_14_@CM, the copper chelator ammonium tetrathiomolybdate (TTM) was employed. As shown in Figure S33, TTM treatment significantly attenuated PMA+Cu_14_@CM‐induced cytotoxicity, leading to a marked recovery in cell viability. The results indicate that the observed cytotoxicity of Cu_14_@CM is primarily mediated by the release of intracellular copper ions and subsequent cuproptosis activation. It is worth noting that Cu_14_@CM shows low toxicity to CT26 and L929 cells (Figures S34 and S35), which might be attributed to the fact that the homologous targeting of the cell membrane leads to less Cu_14_@CM uptake by these two types of cells.

### 2.5 Cuproptosis‐Mediated ICD

**FIGURE 5:**
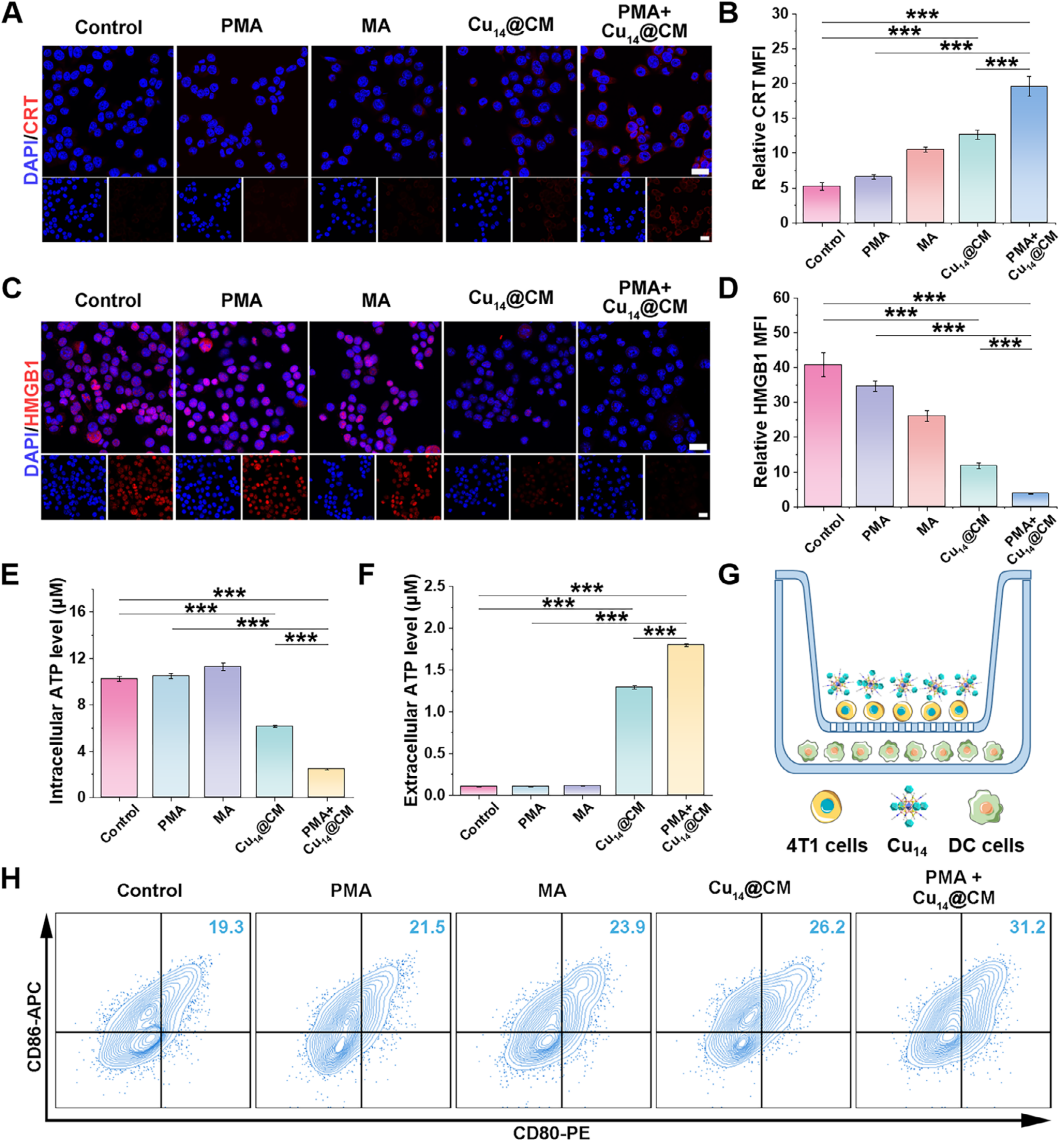
(A) The immunofluorescent staining and (B) quantitative analysis of CRT in 4T1 cells after different treatments. Scale bars, 25 µm. (C) The CLSM images and (D) quantitative analysis of HMGB1 in 4T1 cells after dealing with different groups. Scale bars, 25 µm. (E) Intracellular and (F) extracellular ATP levels of 4T1 cells after different treatments. (G) Schematic illustration and (H) flow cytometric analysis of ICD effect triggered DCs maturation. Data are presented as mean ± s.d. from 3 independent biological replicates. Two‐tailed Student's t‐test were used for comparisons between two independent groups (*p < 0.05, **p < 0.01, ***p < 0.001).

Experimental evidences have demonstrated that cuproptosis could trigger ICD, subsequently eliciting tumor‐specific immune responses. However, the cytoprotective autophagy mechanism maintains cellular homeostasis through selective degradation of damaged macromolecules, a compensatory process that may counteract the antitumor efficacy of cuproptosis induction [14]. Building upon the experimental findings that Cu_14_@CM+PMA enhances cuproptosis efficacy by inhibiting cellular autophagy through prodrug activation, we subsequently investigated the impact of suppressing protective autophagy on Cu_14_@CM‐induced ICD. The DAMPs involving calreticulin (CRT), high mobility group box 1 (HMGB1), and adenosine triphosphate (ATP) were evaluated. As shown in Figure 5A,B, enhanced red fluorescence intensity was observed in the Cu_14_@CM and PMA+Cu_14_@CM groups compared to Control, PMA, and MA treated groups, indicating enhanced CRT release. Notably, autophagy inhibition was found to potentiate CRT release, with the PMA+Cu_14_@CM group exhibiting the most significant effect. Consistent HMGB1 results were validated by CLSM imaging, where nearly undetectable nuclear fluorescence was recorded in the PMA+Cu_14_@CM group, confirming substantial HMGB1 secretion (Figure 5C,D). ATP levels were quantitatively assessed using an ATP Assay Kit, revealing significantly increased extracellular ATP concentration and correspondingly decreased intracellular ATP content in both Cu_14_@CM and PMA+Cu_14_@CM treated groups (Figure 5E,F). Collectively, these findings demonstrated that Cu_14_@CM‐mediated cuproptosis effectively triggered DAMPs release in tumor cells. The co‐treatment of Cu_14_@CM and PMA increases the exposure of DAMPs, which can be attributed to the catalytic generation of MA from PMA by Cu_14_@CM, thereby inhibiting cytoprotective autophagy. Then, the immunomodulatory potential of Cu_14_@CM‐induced ICD was delineated through evaluating the maturation of DCs. The experiments were performed by utilizing mice bone marrow‐derived DCs in a Transwell co‐culture model (Figure 5G). As depicted in Figure 5H, the expression of CD80 and CD86 was observed to be upregulated in DCs treated with Cu_14_@CM and PMA+Cu_14_@CM compared to other groups, with a marked increase in maturation being demonstrated. Notably, the highest level of maturity among all experimental groups was exhibited by the PMA+Cu_14_@CM group. It was demonstrated by the aforementioned results that ICD mediated by Cu_14_@CM‐induced cellular cuproptosis effectively triggered a robust immune response through the promotion of DCs maturation, which could be further intensified by the inhibition of autophagy.

Encouraged by the high anticancer efficacy of Cu_14_@CM‐mediated cuproptosis at the cellular level and its satisfactory immune activation, the in vivo antitumor effects of Cu_14_@CM were further investigated using a subcutaneous 4T1 tumor‐bearing mouse model. Initially, the biocompatibility of Cu_14_@CM was evaluated through cytotoxicity test, hemolysis assay, blood biochemical analysis and histopathological examination. Figure S36 illustrated that Cu_14_@CM at a concentration of 200 µg mL^−1^ exhibited a negligible hemolysis rate confirming its injection safety. Following 14 days of administration in mice, no adverse effects on liver and kidney function were observed in biochemical analyses. And hematological parameters were found to exhibit almost no significant difference compared to the control group (Figures S37 and S38). In addition, no apparent tissue damage in major organs was detected during histopathological examination (Figure S39). All these results confirmed the biosafety of PMA+Cu_14_@CM. Next, the pharmacokinetics and biodistribution of Cu_14_@CM following intravenous administration were analyzed. The copper content in plasma, tumors, and various organs at different time points were quantitatively determined through the use of inductively coupled plasma mass spectrometry. Based on the pharmacokinetic curve, the half‐life of Cu_14_@CM was calculated to be approximately 1.27 h (Figure S40). The gradual accumulation of Cu_14_@CM in tumor tissues following intravenous injection was revealed by the biodistribution results, with a peak concentration of approximately 22.9% ID/g being reached at 12 h (Figure S41).

### 2.6 Therapeutic Effect of Cu_14_@CM In Vivo

**FIGURE 6:**
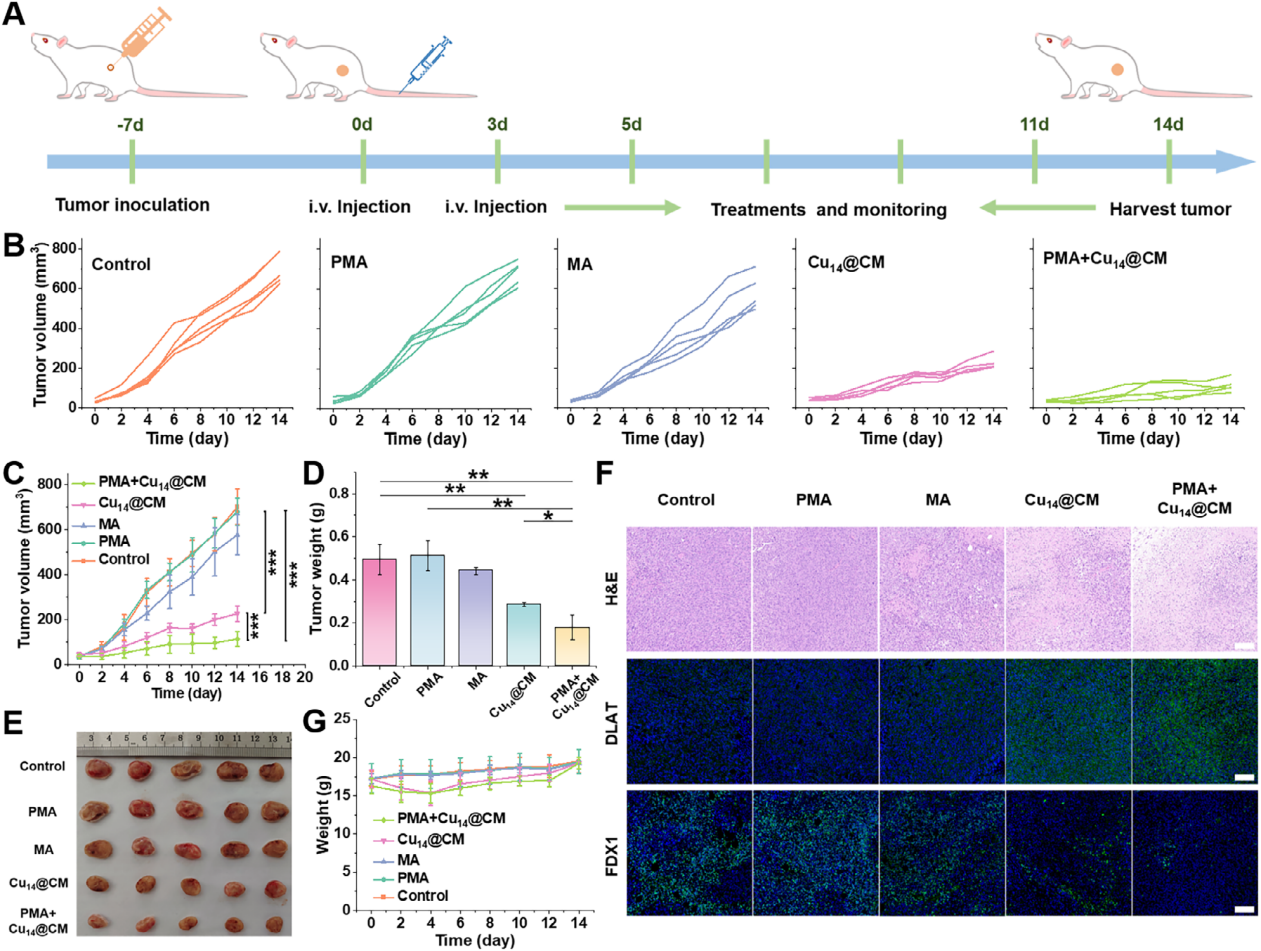
(A) Therapeutic schedule for 4T1 tumor‐bearing mice. (B, C) Tumor growth curves from 1 to 14 days. (D) Mean weight of isolated tumors. (E) Image of the isolated tumor at the end of treatment. (F) H&E, DLAT and FDX1‐stained images of tumor sections from each group of mice. Scale bars, 100 µm. (G) Change of body weight of the tumor‐bearing mice with different treatments. Data are presented as mean ± s.d. from 5 independent biological replicates. Two‐tailed Student's t‐test were used for comparisons between two independent groups (*p < 0.05, **p < 0.01, ***p < 0.001).

The favorable biocompatibility of Cu_14_@CM prompted us to investigate its antitumor efficacy in vivo. The subcutaneous 4T1 tumor‐bearing mouse model was established according to the timeline in Figure 6A. When the tumor volume reached 50 mm^3^, the mice were randomly divided into five groups: Control, PMA, MA, Cu_14_@CM, and PMA+Cu_14_@CM. The tumor volume and body weight were recorded during the treatment regimen. The tumor growth curves showed that tumor volume was significantly suppressed in the Cu_14_@CM treated group, while the PMA+Cu_14_@CM group demonstrated the most effective tumor inhibitory effect due to the synergetic autophagy inhibition mechanism (Figure 6B,C). Ex vivo tumor weights (Figure 6D) and photographs (Figure 6E) corroborated the antitumor efficacy of Cu_14_@CM and PMA+Cu_14_@CM as well. Furthermore, severe cellular damage in tumors of the PMA+Cu_14_@CM group was revealed by hematoxylin and eosin (H&E) staining, characterized by pronounced chromatin condensation, nuclear fragmentation, and disruption of the extracellular matrix (Figure 6F). The terminal deoxynucleotidyl transferase mediated dUTP nick‐end labeling (TUNEL) staining provided the congruent results (Figure S42). Subsequently, the anti‐tumor mechanisms of Cu_14_@CM were analyzed using immunofluorescence staining. Marked aggregation of DLAT and a concomitant decrease in FDX1 were observed in the results, indicating that these effects had been induced by Cu_14_@CM treatment. Notably, enhanced therapeutic efficacy was demonstrated by the PMA+Cu_14_@CM group, which was attributed to autophagy‐inhibitory effects (Figure 6F). Additionally, no significant changes in body weight were observed in any group during treatment (Figure 6G). And the H&E staining of major organs revealed no obvious tissue damage (Figure S43), indicating the favorable biosafety.

### 2.7 Cu_14_@CM‐Induced Immune Response In Vivo

**FIGURE 7:**
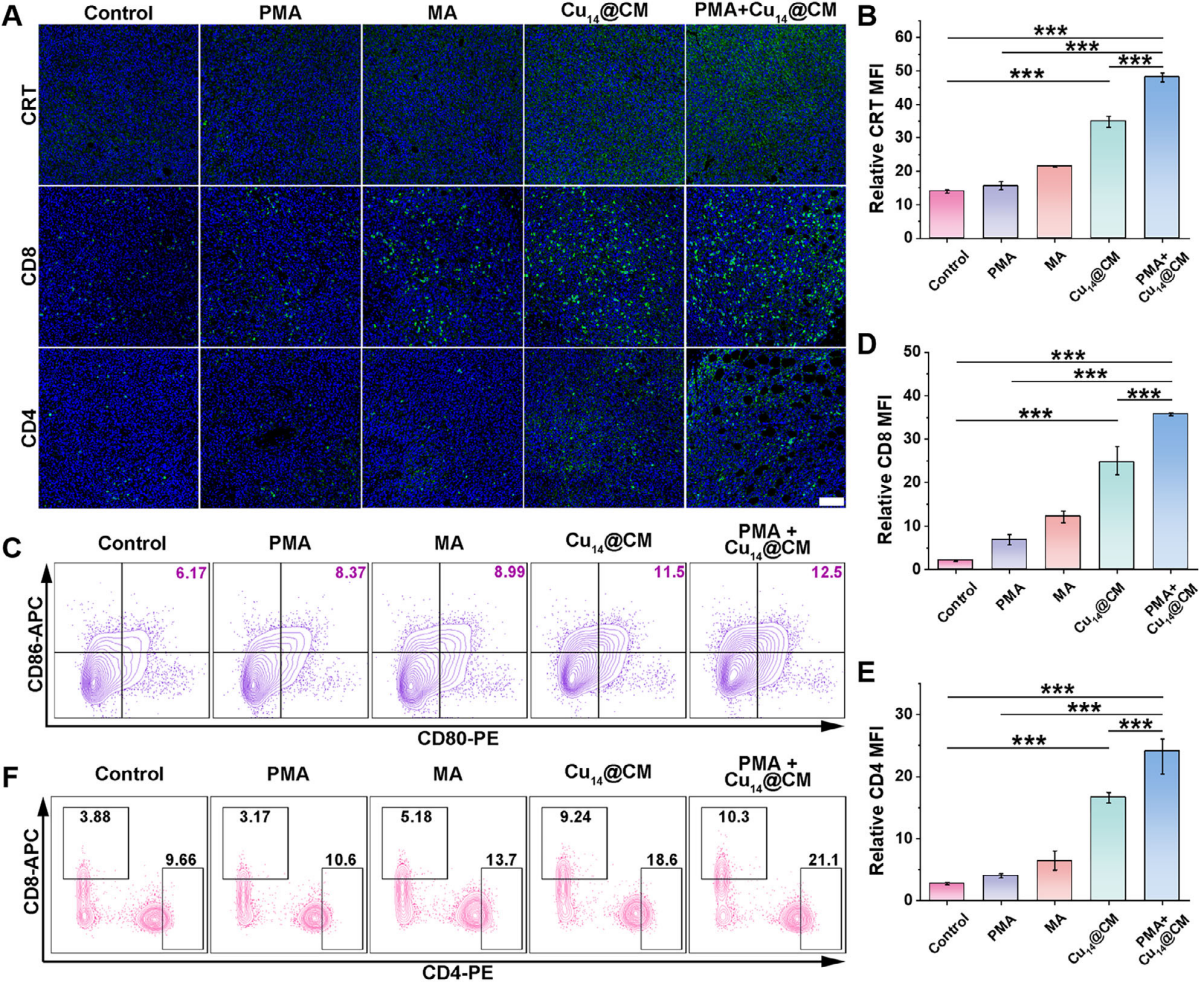
(A) Immunofluorescence staining for CRT, CD8^+^ and CD4^+^. Scale bars, 100 µm. (B) The mean fluorescence intensity of CRT in Figure 7A. (C) Flow cytometric analysis of mature DCs in tumor tissues. The mean fluorescence intensity of (D) CD8^+^ and (E) CD4^+^ in Figure 7A. (F) Flow cytometric analysis of CD4^+^ and CD8^+^ T cells in spleen tissues. Data are presented as mean ± s.d. from 3 independent biological replicates. Two‐tailed Student's t‐test were used for comparisons between two independent groups (*p < 0.05, **p < 0.01, ***p < 0.001).

After that, the cuproptosis‐mediated anticancer immune response was further evaluated in vivo. A significantly higher expression of CRT in tumor tissues treated with PMA+Cu_14_@CM compared to other groups was demonstrated by immunofluorescence staining results, indicating the massive release of DAMPs from tumor cells (Figure 7A,B). Then the tumor‐infiltrating immune cells were analyzed by flow cytometry. A DCs maturation rate of 12.5% was observed in PMA+Cu_14_@CM‐treated tumor tissues, which was approximately 2.03‐fold higher than that in the control group, demonstrating effective promotion of DCs maturation (Figure 7C). Moreover, tumor‐associated macrophage (TAM) in PMA+Cu_14_@CM‐treated tissues exhibited a phenotypic shift from the M2 to the M1 phenotype (Figure S44). The mature DCs and the reprogrammed TAM are capable of mediating downstream immune responses through the modulation of T cell proliferation [47, 48]. Therefore, the levels of cytotoxic T lymphocytes (CD8^+^ T cells), helper T lymphocytes (CD4^+^ T cells) and regulatory T cells (Tregs, CD4^+^FOXP3^+^ T cells) were measured. Increases in the proportions of CD3^+^CD4^+^ and CD3^+^CD8^+^ T cells in tumors were observed after Cu_14_@CM and PMA+Cu_14_@CM treatments (Figure S45). The immunofluorescence staining of tumors exhibited the similar results (Figure 7A,D,E). In contrast, treatment with Cu_14_@CM reduced the proportion of Tregs in tumor tissues, with the PMA+Cu_14_@CM group exhibiting an even lower level of Tregs infiltration (Figure S46). Moreover, the proportions of CD3^+^CD4^+^ and CD3^+^CD8^+^ T cells in mouse spleens were also raised after Cu_14_@CM and PMA+Cu_14_@CM treatments (Figure 7F). These collective findings demonstrated the robust antitumor immunocompetence of Cu_14_@CM and PMA+Cu_14_@CM. The stronger immune response induced by PMA+Cu_14_@CM can be attributed to its catalysis of PMA to produce MA, which inhibits protective autophagy and enables the release of more DAMPs from cuproptosis‐induced ICD.

### 2.8 The Long‐Term Therapeutic Effect of Cu_14_@CM

**FIGURE 8:**
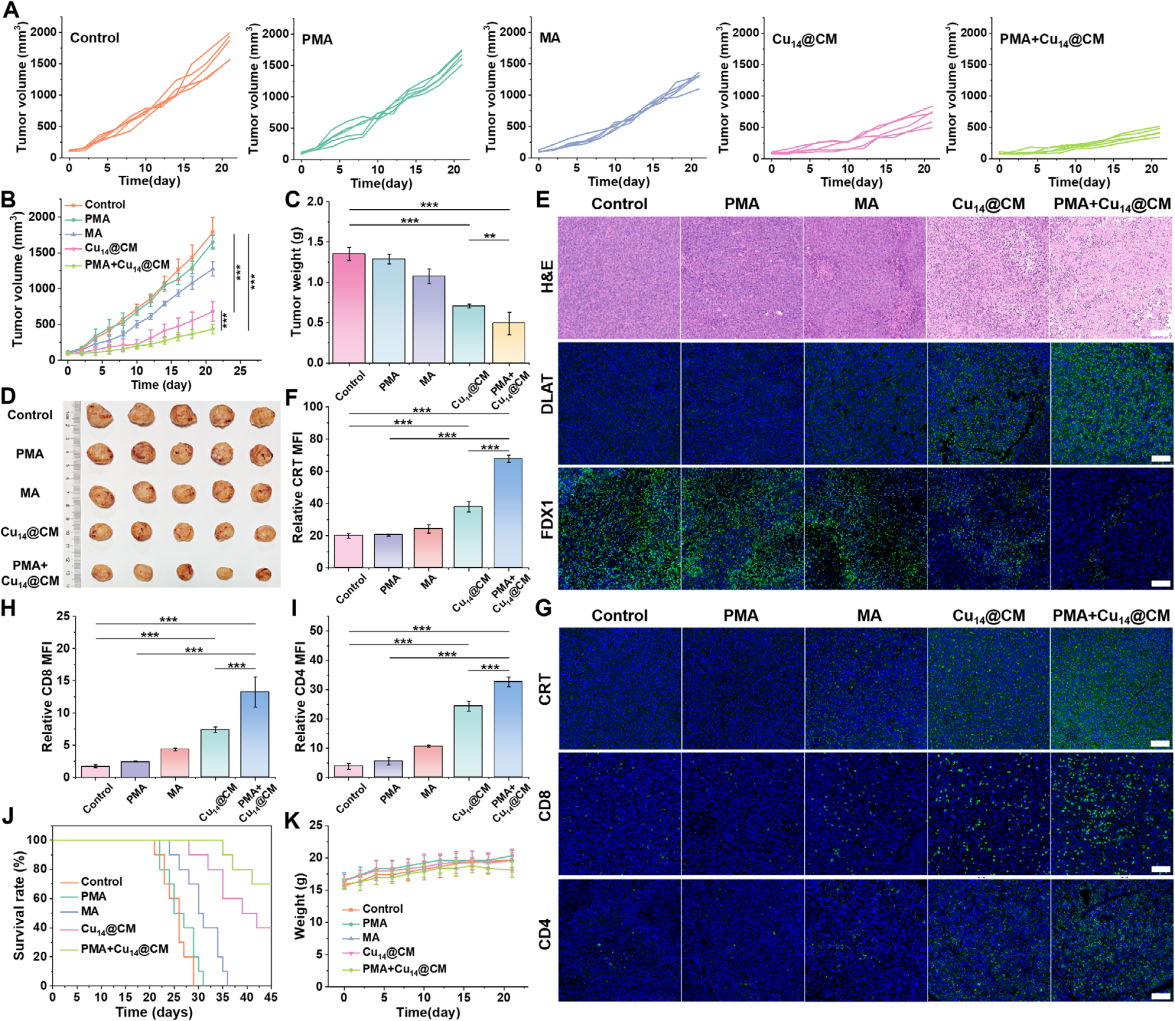
(A, B) Tumor growth curves from 1 to 21 days. (C) Mean weight of isolated tumors. (D) Image of the isolated tumor at the end of the 21‐day treatment regimen. (E) H&E, DLAT and FDX1‐stained images of tumor sections from each group of mice. Scale bars, 100 µm. (F) The mean fluorescence intensity of CRT in Figure 8G. (G) Immunofluorescence staining for CRT, CD8^+^ and CD4^+^. Scale bars, 100 µm. (H, I) The mean fluorescence intensity of CD8^+^ and CD4^+^ in Figure 8G. Fluorescence intensity data are presented as mean ± s.d. from 3 independent biological replicates. (J) The survival curve of mouse model bearing 4T1 tumors after different treatments. (K) Body weight changes in 4T1 tumor‐bearing mice across different treatment groups during the 21‐day treatment regimen. Mouse data are presented as mean ± s.d. from 5 independent biological replicates. Two‐tailed Student's t‐test were used for comparisons between two independent groups (*p < 0.05, **p < 0.01, ***p < 0.001).

Based on the aforementioned effective inhibition of tumor growth by Cu_14_@CM, its long‐term efficacy was further investigated. A subcutaneous mouse model bearing 4T1 tumors was re‐established. When tumor volumes reached approximately 100 mm^3^, the mice were randomly divided into five groups: Control, PMA, MA, Cu_14_@CM, and PMA+Cu_14_@CM. Tumor volume and body weight changes in each group were continuously monitored throughout a 21‐day treatment period. The results showed significantly suppressed tumor growth in the Cu_14_@CM group, whereas the PMA+Cu_14_@CM group exhibited the most potent antitumor effect, attributable to synergistic autophagy inhibition (Figure 8A,B). The superior and sustained antitumor activity of PMA+Cu_14_@CM was further confirmed by ex vivo measurements of tumor weight (Figure 8C) and gross morphological observation (Figure 8D). Moreover, H&E staining revealed severe tumor cell damage in the PMA+Cu_14_@CM group, characterized by chromatin condensation, nuclear fragmentation, and disruption of the extracellular matrix structure (Figure 8E). Subsequent immunofluorescence staining confirmed DLAT protein aggregation and a corresponding decrease in FDX1 protein levels, indicating that the long‐term antitumor effect of Cu_14_@CM is mediated via the cuproptosis pathway. Furthermore, Figure 8F,G demonstrated an increase in CRT expression in PMA+Cu_14_@CM‐treated tumors compared to other groups, indicating substantial release of DAMPs. In addition, elevated proportions of CD3^+^CD4^+^ T cells and CD3^+^CD8^+^ T cells within tumor tissues were observed in tumor tissues following treatment with either Cu_14_@CM or PMA+Cu_14_@CM (Figure 8G‐I), demonstrating the immune responses regulated by cuproptosis. Survival analysis demonstrated that mice treated with Cu_14_@CM exhibited significantly prolonged survival. After 45 days of treatment, the PMA+Cu_14_@CM group showed the highest survival rate (Figure 8J). Collectively, these results confirm the potent antitumor and immune‐activating effects of PMA+Cu_14_@CM, supporting its long‐term effectiveness.

Thereafter, long‐term safety of the drugs in vivo was evaluated. Body weight measurements, blood biochemical analysis and histopathological examination were performed on treated mice. No significant changes in body weight were observed in any group during the entire treatment period (Figure 8K). Figure S47 confirmed the absence of adverse effects on liver and kidney functions after the treatment period, with all indices remaining within normal ranges. Meanwhile, no obvious differences in hematological parameters were observed in PMA+Cu_14_@CM group. Additionally, H&E staining of major organs revealed no apparent signs of tissue damage, indicating good biocompatibility of Cu_14_@CM and PMA during long‐term treatment.

With respect to translational considerations, we further discussed the long‐term fate of Cu_14_@CM in vivo. It is well established that copper is an essential trace element required for maintaining normal physiological functions in the human body. Previous studies have confirmed that the liver and spleen are the primary organs for copper storage, with its metabolism predominantly relying on the liver. Excess copper is typically excreted via bile (the primary route for endogenous copper elimination) into the intestine and ultimately eliminated through feces [49, 50]. Furthermore, extensive research has demonstrated that the mononuclear phagocyte system (MPS), including macrophages in the liver, spleen, and lymph nodes, constitutes the primary route for the clearance of exogenous nanomaterials [51, 52]. Therefore, the copper accumulation observed in the liver and spleen (Figure S41) is likely attributable to this inherent clearance mechanism for exogenous materials. This accumulation pattern aligns with the natural metabolic pathway of copper and appears to be transient. The temporarily retained copper in these organs holds significant potential to be gradually degraded and subsequently reutilized or excreted in systemic metabolism through the aforementioned pathways.

### 2.9 In Vivo Treatment of Orthotopic Tumors

**FIGURE 9:**
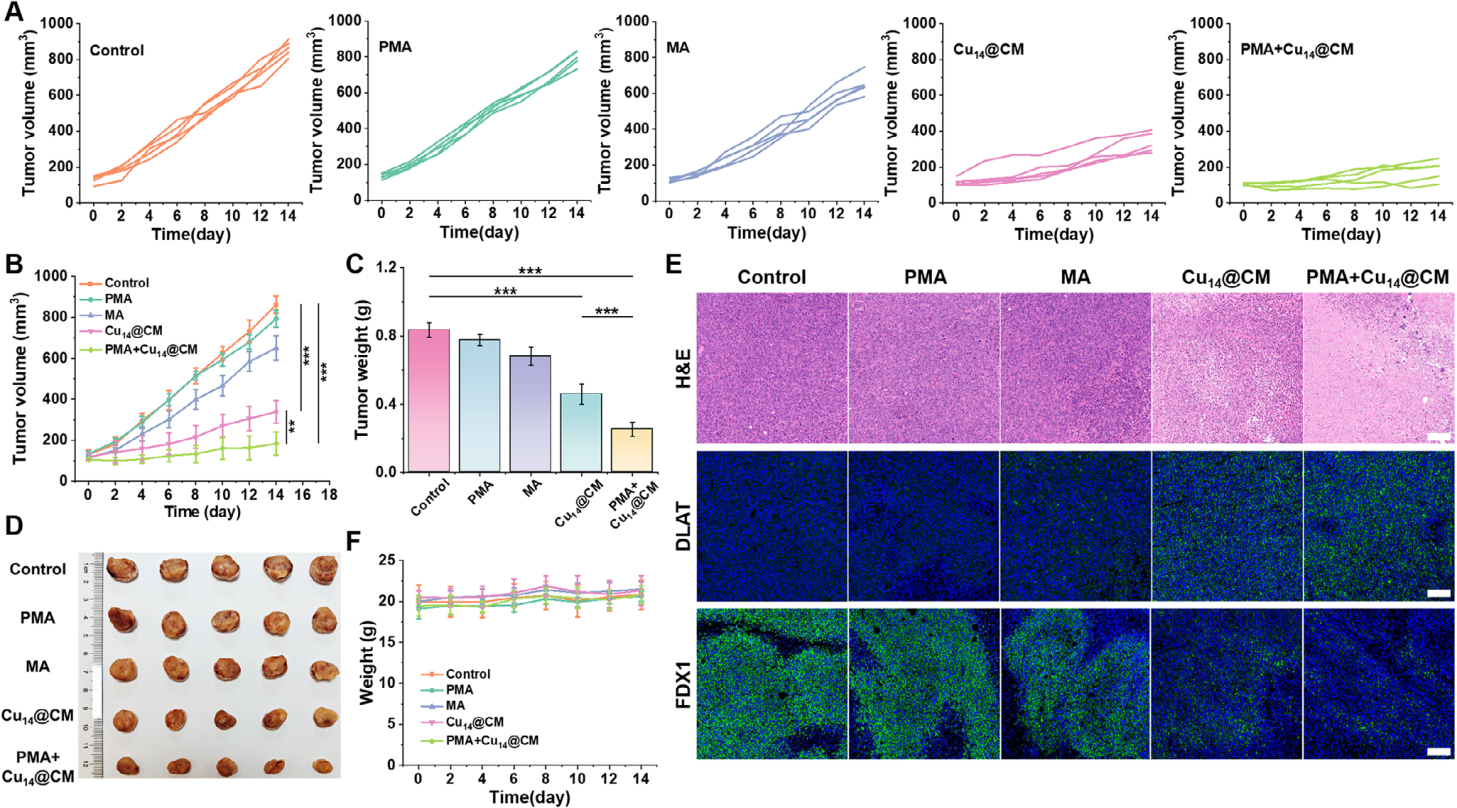
(A, B) Orthotopic breast tumors growth curves during the 14‐day treatment period. (C) Mean weight of isolated orthotopic breast tumors. (D) Image of the isolated tumors at the end of treatment. (E) H&E, DLAT and FDX1‐stained images of orthotopic tumors sections from mice in different treatment groups. Scale bars, 100 µm. (F) Body weight changes of orthotopic 4T1 tumor‐bearing mice in the various treatment groups over the course of treatment. Data are presented as mean ± s.d. from 5 independent biological replicates. Two‐tailed Student's t‐test were used for comparisons between two independent groups (*p < 0.05, **p < 0.01, ***p < 0.001).

To more directly evaluate the efficacy of the Cu_14_@CM‐activated autophagy inhibitor prodrug in enhancing cuproptosis‐based immunotherapy, orthotopic tumor models were established to better recapitulate the microenvironment of primary tumors and their stromal interactions. Orthotopic tumors were established by implanting 4T1 cells into the mammary fat pads of female BALB/c mice. Primarily, the biodistribution of Cu_14_@CM in orthotopic tumor‐bearing mice was studied. Figure S48 showed that the concentration of Cu_14_@CM reached a peak of approximately 16.96 % ID/g at 24 h post intravenous injection, indicating effective accumulation of Cu_14_@CM in the orthotopic tumor tissue and providing a basis for subsequent experiments. Subsequently, the tumor‐bearing mice were administered PBS, PMA, MA, Cu_14_@CM, and PMA+Cu_14_@CM, respectively. While maintaining the same treatment regimen as used in the subcutaneous model, tumor volume and body weight changes were monitored continuously across all groups. Tumor growth curves demonstrated that Cu_14_@CM effectively suppressed orthotopic tumor growth. Owing to the synergistic effect of cuproptosis and autophagy inhibition, the PMA+Cu_14_@CM group exhibited the most potent tumor‑suppressive activity. Moreover, ex vivo tumor weight measurements and corresponding photographs further confirmed the pronounced inhibitory effect of PMA+Cu_14_@CM on orthotopic tumors. Apart from these, H&E staining revealed obvious nuclear fragmentation and severe disruption of the extracellular matrix structure in orthotopic tumor tissues treated with PMA+Cu_14_@CM, further supporting its antitumor efficacy. Simultaneously, immunofluorescence staining images indicated that Cu_14_@CM treatment induced intracellular DLAT protein oligomerization and significantly downregulated FDX1 expression, confirming that its antitumor mechanism is mediated through the cuproptosis pathway. Additionally, the enhanced therapeutic outcome in the PMA+Cu_14_@CM group was attributed to the autophagy‑inhibitory effect. Notably, no significant changes in body weight were observed in any group throughout the treatment period (Figure 9F). Collectively, these results demonstrate that PMA+Cu_14_@CM possesses strong antitumor activity against orthotopic breast cancer.

## 3 Conclusions

In summary, to mitigate the impact of defensive autophagy on ICD and reduce systemic drug toxicity, this study pioneers a Cu_14_ cluster‐mediated bond cleavage reaction for synergistically inducing cuproptosis‐associated ICD while suppressing autophagy, thereby enhancing the immune response. The engineered Cu_14_@CM with the ability of homologous targeting demonstrated dual functionality, triggering cuproptosis cascades via lipoylated protein aggregation and Fe‐S cluster destabilization through pH‐responsive copper ion liberation, while simultaneously executing site‐specific bond cleavage to generate autophagy inhibitors, collectively amplifying DAMPs exposure. This coordinated strategy enhanced antitumor immunity via DCs activation and tumor‐infiltrating lymphocyte proliferation, establishing a new frontier in metal cluster therapeutics.

## Conflicts of Interest

The authors declare no conflict of interest.

## Supporting information




**Supporting File**: advs75025‐sup‐0001‐SuppMat.docx.

## Data Availability

The data that support the findings of this study are available from the corresponding author upon reasonable request.
