# Supplementary material for: Highly Active Cu14 Cluster Precisely Activates Autophagy Inhibitor to Amplify Cuproptosis Immunotherapy
Source: Adv Sci (Weinh). 2026 Mar 26;13(33):e17021. doi: 10.1002/advs.202517021 (PMC13271629; doi:10.1002/advs.202517021)
Supplement: Supplementary file 1 — Supporting File: advs75025‐sup‐0001‐SuppMat.docx. [file ADVS-13-e17021-s001.docx]

Supporting Information

HIGHLY ACTIVE CU_14_ CLUSTER PRECISELY ACTIVATES AUTOPHAGY INHIBITOR TO AMPLIFY CUPROPTOSIS IMMUNOTHERAPY

Qiu-Xu Zang, Wei-Tong Chen, Yu-Ying Shen, Zhao-Yang Wang, Kai Li, Xueli Zhao, Yanjuan Sang,* Xiaoyuan Chen,* Shuang-Quan Zang*

Q.-X. Zang, W.-T. Chen, Y.-Y. Shen, Z.-Y. Wang, K. Li, X. Zhao, Y. Sang, S.-Q. Zang

State Key Laboratory of Metabolic Dysregulation & Prevention and Treatment of Esophageal Cancer, Henan International Joint Laboratory of Tumor Theranostic Cluster Materials, College of Chemistry, Zhengzhou University, Zhengzhou, Henan 450001, P. R. China

Q.-X. Zang, W.-T. Chen, Y.-Y. Shen, Z.-Y. Wang, K. Li, X. Zhao, Y. Sang, S.-Q. Zang

Tianjian Laboratory of Advanced Biomedical Sciences, Institute of Advanced Biomedical Sciences, Zhengzhou University, Zhengzhou, Henan 450001, P. R. China

X. Chen

Shandong Provincial Key Laboratory of Precision Oncology, Shandong Cancer Hospital and Institute, Shandong First Medical University and Shandong Academy of Medical Sciences, Jinan, Shandong 250117, P. R. China.

**Experimental section**

**Materials and reagents:** All solvents used are commercially available reagent grade and require no further purification. 1,2-dithiol-o-carborane were prepared by a literature method.^[1]^ Copper(II)trifluoroacetate (Cu(CF_3_COO)_2_) and 3,3',5,5'-tetramethylbenzidine (TMB) were obtained from Energy Chemical Co., Ltd. 2-methylbut-3-yn-2-yl (3-methyl-3H-purin-6-yl)carbamate (PMA) was purchased from Shanghai Bide Pharmatech Co., Ltd. RPMI-1640 and Dulbecco's phosphate-buffered saline (DPBS) were purchased from Corning inc. Trypsin-EDTA solution was obtained from Beijing Solarbio Science & Technology Co., Ltd. 4',6-diamidino-2-phenylindole (DAPI) was purchased from Sigma Aldrich. Cell Counting Kit-8 (CCK-8) and ATP Assay Kit were acquired from Beyotime Biotechnology Co., Ltd. JC-1 Mitochondrial Potential Sensor, LIVE/DEAD™ Cell Imaging Kit, CellROX™ Deep Red Reagent and Highly Cross-Adsorbed Secondary Antibody Alexa Fluor™ 647 goat anti-rabbit IgG (H+L) were received from Thermo Fisher Scientific. Triton®X-100 was purchased from Guangzhou Saiguo Biotech Co., Ltd. Annexin V-FITC/PI double staining apoptosis detection Kit was obtained from Bestbio Biotechnology Co., Ltd. DLAT Polyclonal antibody (Catalog No.: 13426-1-AP), LIAS Polyclonal antibody (Catalog No.: 11577-1-AP), FDX1 rabbit polyclonal antibody (Catalog No.: 12592-1-AP), HMGB1 Polyclonal antibody (Catalog No.: 10829-1-AP) and Calreticulin Polyclonal antibody (Catalog No.: 10292-1-AP) were received from Proteintech. SDHB rabbit polyclonal antibody (Catalog No.: GB111344), Aconitase 2 rabbit polyclonal antibody (Catalog No.: GB113832) and recombination Anti-beta Actin antibody (Catalog No.: GB15003) were acquired from Servicebio (Wuhan, P.R. China). FITC labeled anti-Mo CD11c, APC labeled anti-Mo CD86 (B7-1), PE labeled anti-Mo CD80 (B7-2), FITC labeled anti-Mo CD3, APC labeled anti-Mo CD8, and PE labeled anti-Mo CD4, PE labeled anti-Mo FOXP3, APC labeled anti-Mo CD4, PE labeled anti-Mo CD163 and FITC labeled anti-Mo F4/80 were purchased from BioLegend, Inc. 4T1, L929 and CT26 cells were obtained from the China Center for Type Culture Collection (Wuhan, P.R. China). BALB/c SPF mice (5-week-old females) were purchased from Beijing Vital River Laboratory Animal Technology Co., Ltd.

**Instrumentation:** Powder X-ray diffraction (PXRD) patterns of the samples were recorded on a D/MAX-3D diffractometer (Cu Kα, *λ* = 1.54178 Å). Mass spectra were available on an X500R QTOF spectrometer. Transmission electron microscopy (TEM) images were obtained by using a Tecnai G2 F20 STWIN transmission electron microscope (performed at an accelerating voltage of 200 kV). Fourier transform infrared (FT-IR) spectroscopy was measured on a Bruker TENSOR 27 Fourier transform infrared spectrometer. Zeta potential measurement was performed on a Malvern Panalytical Zetasizer Pro. UV-Vis absorption spectra were recorded on TU-1901 double-beam UV-Vis spectrophotometer. The emission spectra were measured on a HORIBA FluoroLog-3 fluorescence spectrometer. The HPLC data was recorded on the Shimadzu Nexera LC-40D RX high-performance liquid chromatography system. The HPLC analysis was performed on a Shimadzu Nexera LC-40D RX system. An Ultimate XB-C18 column (250 mm × 4.6 mm, 5 μm) from Welch Materials, Inc. was used. The column temperature was set at 40 °C. A mixture of methanol and water in a ratio of 1:4 (v/v) was employed as the mobile phase, with a flow rate of 1 mL/min. The fluorescence imaging of cells was monitored by a Leica TCS SP8 confocal laser scanning microscope (CLSM). The X-ray photoelectron spectroscopy (XPS) measurements were performed with a Thermo Scientific K-Alpha instrument with an Al K*α* X-ray resource. Flow cytometry experiment was carried out on a BD FACSymphony™ A5.

**Synthesis of** **Cu_14_ cluster:** Cu_14_ was prepared according to the literature method with slight modifications.^[1]^ A solution of 1,2-dithiol-o-carborane (0.05 mmol) in 40 mL of THF was added dropwise to an acetonitrile (40 mL) solution of Cu(CF_3_COO)_2_ (0.05 mmol) under stirring. The resultant light yellow solution was evaporated slowly at room temperature for 5 days to afford light green block crystals suitable for SCXRD analysis.

**Preparation of cell membrane:** The 4T1 cells were suspended in a hypotonic lysis buffer containing membrane protein extraction reagent and phenylmethylsulfonyl fluoride (PMSF) and incubated on ice bath for 10-15 minutes. Afterwards, the cells in the above solution were broken repeatedly by freeze-thawing and then centrifuged at 700 g for 10 min at 4 ^o^C. Then, the supernatant was centrifuged at 14,000 g for 30 minutes to collect cell membrane fragments. Afterwards, cell membrane fragments were washed with PBS. Membrane products were lyophilized overnight, weighed and stored at -80 °C. The membrane material was rehydrated in ultrapure water before use.

**Preparations of Cu_14_@CM:** Cu_14_@CM was fabricated by coating Cu_14_ with cancer cell membrane fragments by an extrusion method. Specifically, cell membrane fragments from 4T1 cells were mixed with Cu_14_ and then sonicated for 2 minutes. Then the mixture sequentially extruded through 200 nm polycarbonate porous membranes using a miniextruder for at least 20 cycles. The obtained Cu_14_@CM was centrifuged and washed several times with PBS.

**Stability measurement:** The Cu_14_@CM was dispersed into PBS, and UV-Vis absorption spectra were recorded at different time intervals.

**Detection of ·OH:** The capability of the Cu_14_ cluster to catalyze •OH generation from H_2_O_2_ was systematically investigated via the TMB oxidation assay. Solution absorption spectra under varying conditions, including different pH values, concentrations of the Cu_14_ cluster, TMB, and H_2_O_2_, were recorded using UV-Vis spectroscopy over the wavelength range of 500 to 800 nm.

**Cell culture:** The 4T1 and CT26 cells were cultured in RPMI-1640 and L929 cells were cultured in Minimum Essential Medium, which was supplemented with 10% fetal bovine serum and 1% Penicillin-Streptomycin at 37 °C in a humidified atmosphere of 5% CO_2_.

**Cytotoxicity analysis:** The 4T1 cells were inoculated in 96-well plate at a density of approximately 10^4^ cells per well. After attachment, the cells were treated with PBS, PMA , 3-MA , Cu_14_@CM, and PMA+Cu_14_@CM, respectively. 24 h later, 10 μL of CCK-8 reagent was added to the wells for 1 h. A multimode plate reader was utilized to record its absorbance at 450 nm. The relative cell viability (%) was calculated as follows: (As-Ab)/(Ac-Ab) × 100% (As: Experiment; Ab: Blank; Ac: Control).

**Cellular uptake behaviors of Cu_14_@CM:** The 4T1 cells were seeded in 24-well plate with an initial density of 10^5^ cells/well for overnight incubation. After that, Cu_14_@CM was added and incubated for different times (0, 3 and 6 h). Subsequently, the nucleus was stained with DAPI. The confocal imaging was performed with the Leica TCS SP8 CLSM.

**Prodrug activation model reaction inside living cells:** The 4T1 cells were randomly cultured in 6-well plates at an initial density of 5×10⁵ cells per well overnight. Subsequently, Cu_14_@CM (100 μg mL^-1^) was incubated with the attached cells. After 6 h, the old medium was removed, and cells were washed three times with PBS to remove extracellular nanoparticles. Pro-Cou was added to a final concentration of 1 mM and incubated for 24 h. Thereafter, the treated cells were washed twice with PBS and analyzed by flow cytometry and CLSM.

**Intracellular reactive oxygen species (ROS) detection:** The 4T1 cells were randomly cultured in 24-well plate with an initial density of 10^5^ cells per well for overnight. Then, the cells were processed using different methods. After that, the cells were stained with the CellROX Deep Red Reagent. Subsequently, the nucleus was stained with DAPI. The confocal imaging was performed with the Leica TCS SP8 CLSM (excited at 405 nm and 552 nm).

**Live/dead imaging****:** The cell culture and treatment were same to the experiment of intracellular ROS detection. After that, LIVE/DEAD cell imaging working solution was added and confocal imaging was performed with the Leica TCS SP8 CLSM (excited at 488 nm and 552 nm).

**Flow cytometry experiment:** The cell culture and treatment were same to the experiment of intracellular ROS detection. After that, the cells were harvested and stained with Annexin V-FITC/PI double staining Kit, and analyzed by flow cytometry.

**Mitochondrial membrane potential:** The 4T1 cells were randomly cultured in 24-well plate with an initial density of 10^5^ cells per well for overnight. Then, the cells were processed using different methods. After that, the cells were stained with the MitoProbe JC-1. The confocal imaging was performed with the Leica TCS SP8 CLSM (excited at 488 nm and 552 nm).

**Western blot analysis:** The cell culture and treatment were same to the experiment of intracellular ROS detection. After that, the cells were washed thoroughly with ice-cold DPBS and then lysed in RIPA Lysis Buffer. The lysates were centrifuged at 4 °C at 10000 rpm for 10 min. The protein concentration was measured by using the protein concentration determination kit according to the manufacturer's protocol. The sample was separated using 10% SDS-PAGE. The isolated proteins were transferred to polyvinylidene fluoride (PVDF) membranes and closed with 5% skim milk powder for 2 h at room temperature. Subsequently, the membrane was incubated overnight with primary antibodies as follows: anti-DLAT, anti-FDX1, anti-SDHB, anti-LISA and anti-ACO-2. Then, the membrane was further incubated with goat anti-rabbit secondary antibody with the horseradish peroxide (HRP)-labeled for 2 h at 4 °C. At last, the protein bands were visualized using an enhanced chemiluminescence system.

**Adenosine triphosphate (ATP) release detections:** 4T1 cells were seeded randomly in 24-well plates at an initial density of 10^5^ cells per well and incubated overnight. Subsequently, the cells were treated using different methods. ATP release was then measured using an ATP assay kit according to the manufacturer's protocol.

**DLAT, CRT and HMGB1 expression:** The 4T1 cells were randomly cultured in 24-well plate with an initial density of 10^5^ cells per well for overnight. Then, the cells were processed using different methods. After that, the cells were fixed with 4% paraformaldehyde and permeabilized by 1% FBS + 0.25% Triton-X in DPBS at room temperature for 30 min. The cells were blocked with 1% BSA at room temperature for 1 h. The treated 4T1 cells were further incubated with anti-DLAT antibody, anti-CRT antibody or anti-HMGB-1 antibody in 1% BSA containing PBS solution at 4 °C for 12 h. Then, 4T1 cells were further incubated with secondary antibody (Alexa Fluor® 647-cojugated goat anti-rabbit IgG H&L) at 4 °C for another 1 h. Finally, the DLAT, CRT and HMGB1 expression was measured by Leica TCS SP8 CLSM.

**In vitro DC stimulation:** Bone marrow-derived DCs (BMDCs) were generated from BALB/c mice femurs/tibiae per established protocols. After red blood cells lysis, cells were cultured in RPMI 1640 (10% FBS, 1% penicillin-streptomycin, 20 ng·mL^-1^ GM-CSF, 20 ng·mL^-1^ IL-4). Medium was replenished (5 mL fresh) on day 3. On day 5, half the supernatant was replaced with fresh medium. BMDCs were harvested on day 7 from non-adherent/loosely adherent fractions. 4T1 cell residues after different treatments were introduced to DC cultures via transwell. Treated DCs were stained with FITC anti-mouse CD11c, PE anti-mouse CD80, APC anti-mouse CD86 and analyzed by flow cytometry.

**Animal study:** BALB/c SPF female mice (5 weeks old) were purchased from Vital River. The study was approved by the Institutional Animal Care and Use Committee of the Zhengzhou University.

**Hemolysis measurement:** 1 mL blood was collected in tube containing EDTA from the orbital venous of BALB/c mice and mixed with appropriate amount of 10 mM PBS (pH = 7.4). After centrifuging and washing with PBS for 3~4 times, the precipitated erythrocytes were then dispersed in PBS. The Cu_14_@CM was added to the erythrocyte suspension. Negative control was placed with erythrocyte suspension diluted with 10 mM PBS (pH = 7.4). And positive control was placed with erythrocyte suspension diluted with ultrapure water. The mixtures were incubated for 8 h at room temperature. Then the specific 540 nm spectrophotometric absorptions of hemoglobin were analyzed. Calculation of the hemolysis rate (HR%) was by the following equation: HR% = (A_S_-A_NC_) × 100%/(A_PC_-A_NC_). Where A_S_, A_PC_ and A_NC_ are the absorbance of the sample, the positive control and the negative control, respectively.

**Biocompatibility:** Ten healthy female BALB/c mice were selected and randomly divided into two groups, with one group receiving PBS injections and the other intravenously administered PMA+Cu_14_@CM. At the indicated time, the mice were euthanized and their blood samples were collected to perform blood serum biochemistry assay. Furthermore, the main organs (heart, liver, spleen, lung and kidney) and tumor tissues of the mice were isolated and fixed in 10% paraformaldehyde solution. Then the slices were prepared routinely in process and stained with hematoxylin and eosin (H&E), which was observed and captured by a Leica Microsystems CMS GmbH.

**Pharmacokinetic:** Female BALB/c mice were i.v. injected with Cu_14_@CM (100 μL, 10 mg kg^-1^). At 0.25, 0.5, 1, 2, 4, 8, 12, and 24 h post-injection, Minimal blood were collected by puncturing the tail vein. Afterwards, the content of Cu in the samples was determined by ICP-MS.

**Biodistribution:** The 4T1 tumor-bearing mice were randomly divided into 3 groups followed by intravenously injection of 100 μL of Cu_14_@CM (10 mg kg^-1^). The mice were euthanized at 2, 4, 8, 12, 24, and 48 h after injection of materials. Then the tumors and major organs were harvested and ablated. The content of Cu in the samples was determined by ICP-MS.

**In vivo antitumor efficacy:** The 4T1 tumor-bearing mice were weighed and randomly divided into 6 groups: (1) Control, (2) PMA, (3) MA, (4) Cu_14_@CM, (5) PMA+Cu_14_@CM. Then, Group 1 received an intravenous injection of PBS (100 μL); Group 2 was administered PMA (100 μL, 100 μmol kg^-1^) via intraperitoneal injection; Group 3 received MA (100 μL, 100 μmol kg^-1^) through intraperitoneal injection; Group 4 was intravenously injected with Cu_14_@CM (10 mg kg^-1^); Group 5 received a combination of intravenous Cu_14_@CM (10 mg kg^-1^) and intraperitoneal PMA (100 μL, 100 μmol kg^-1^). The mice were treated and weighted every other day for 14 days. Also, the length (L) and width (W) of the tumors were measured to calculate the tumor volume (V) according to the following equation: V = L × W^2^/2.

**H&E and TUNEL staining:** For H&E assay, the main organs (heart, liver, spleen, lung, and kidney) and tumor tissues of the mice were isolated at the end of the treatment and fixed in 10% paraformaldehyde solution. Then the slices were prepared routinely in process and stained with H&E, which was observed and captured by a Leica Microsystems CMS GmbH. TUNEL staining was performed using an in situ apoptosis detection kit. DAPI was used to stain the nucleus, followed by the addition of an anti-fluorescence quenching solution. Images were captured using the CLSM.

**Immunofluorescence experiment:** For immunoflourescent staining, tumor tissues of the mice were isolated at the end of the treatment and fixed in 10% paraformaldehyde solution. Then the slices were prepared routinely in process and blocked with serum (5%) for 30 min. After sections’ fixation in acetone at -20 °C, the sections were incubated with primary antibodies including anti-DLAT, anti-CRT, anti-CD4 and anti-CD8 at 4 °C overnight. And then incubated with secondary antibodies for 2 h at room temperature. Immunofluorescence images were acquired with CLSM.

**Statistical analysis:** Concentrations of PMA and MA were calculated by interpolating the corresponding peak areas into standard curves. The data on copper ion release, cell viability, hemolysis and the survival curve of mice were normalized to enable quantitative comparison across experimental conditions. The data on tissue distribution and blood circulation curve of Cu_14_@CM were transformed. All the experimental data were repeated three or more times, and each experiment was independent of the others. Experimental results are presented as the means ± standard deviations (means ± SD).Statistical evaluation was performed using two-tailed Student’s t-test analysis. Asterisks indicate significant difference (**p* < 0.05, ***p* < 0.01, ****p* < 0.001). All statistical analyses were conducted using Microsoft Excel.


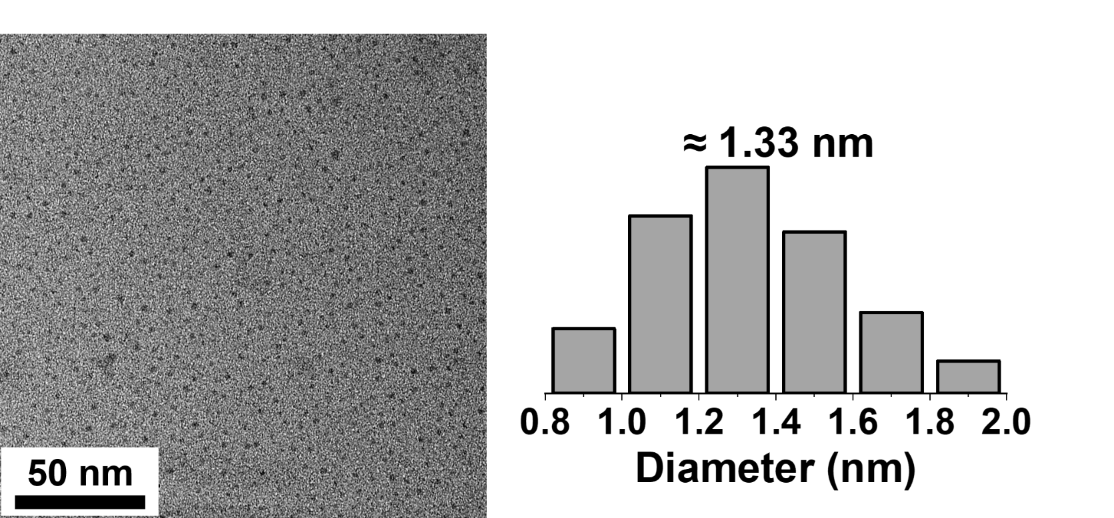


**Figure S1.** The TEM image and size distribution histogram of Cu_14_ cluster.


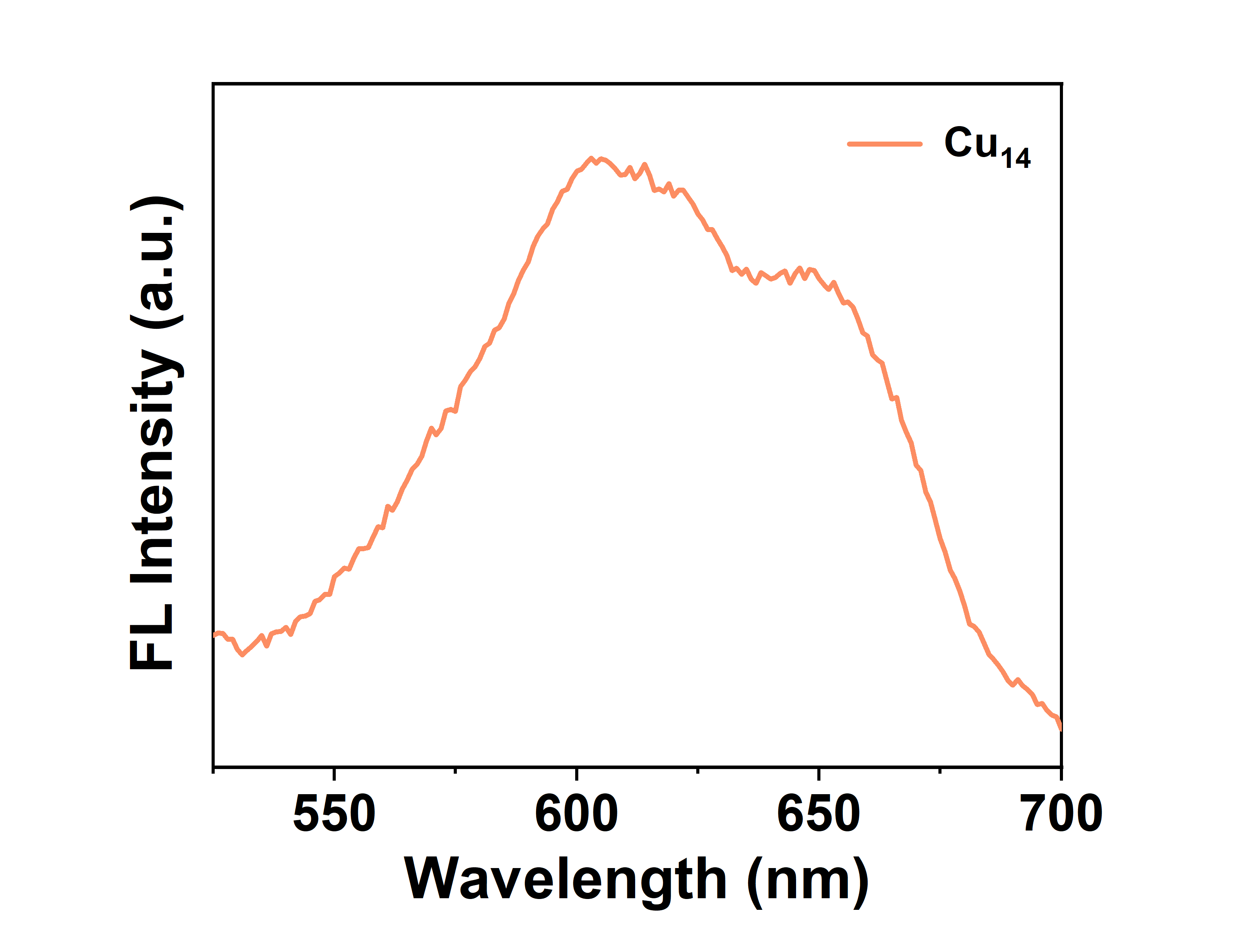


**Figure S2.** The fluorescence spectrum of Cu_14_ cluster.


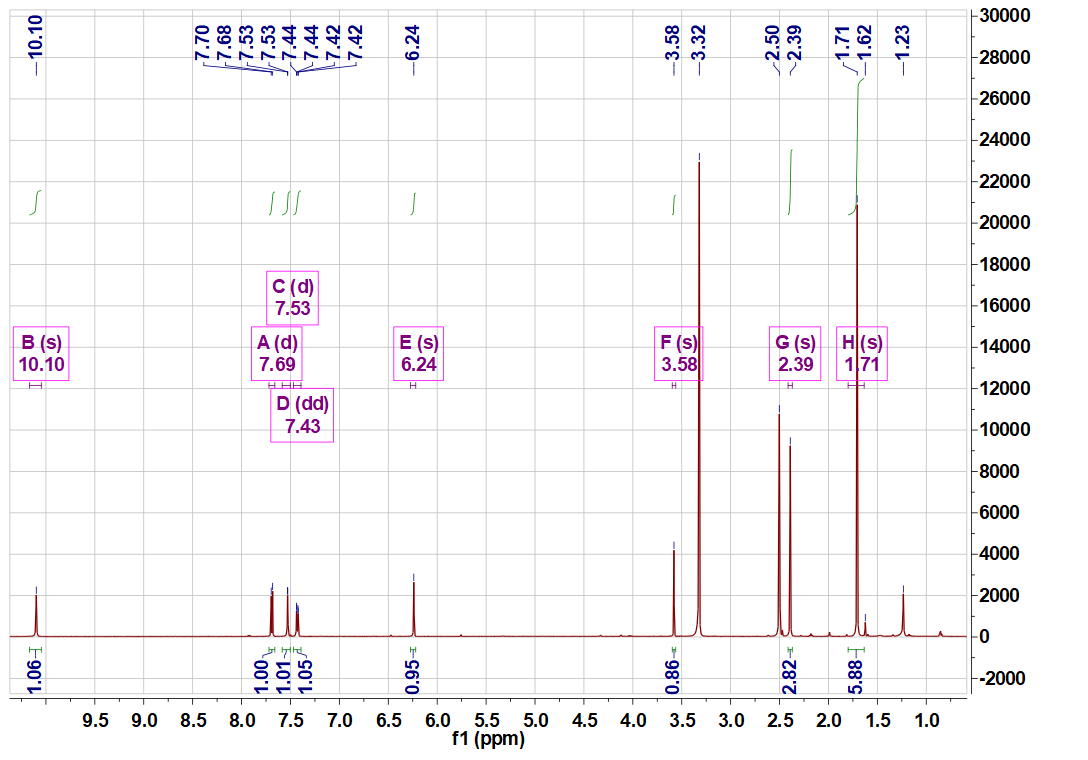


**Figure S3.** Nuclear magnetic resonance hydrogen spectrum of Pro-Cou.

**
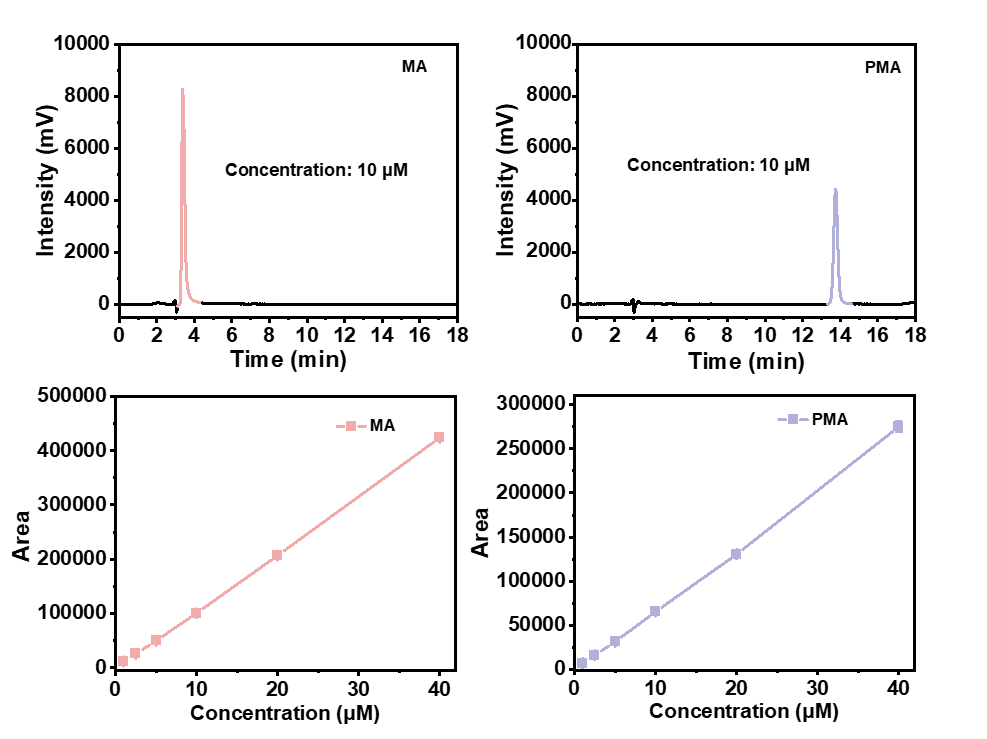
**

**Figure S4.** The HPLC standard curves of PMA and MA. Data are presented as mean ± s.d. from 3 independent biological replicates.


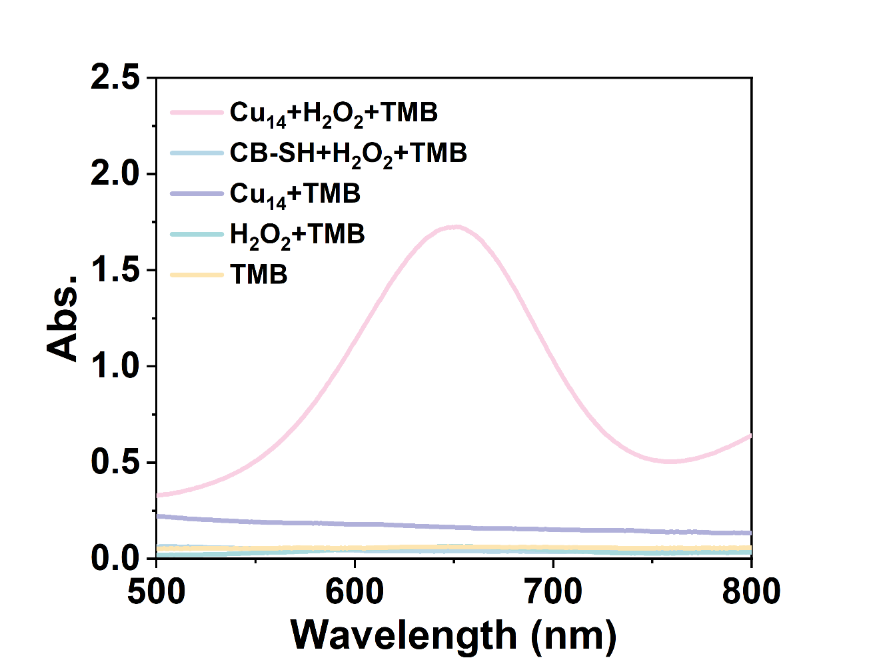


**Figure S5.** The generation of •OH from H_2_O_2_.


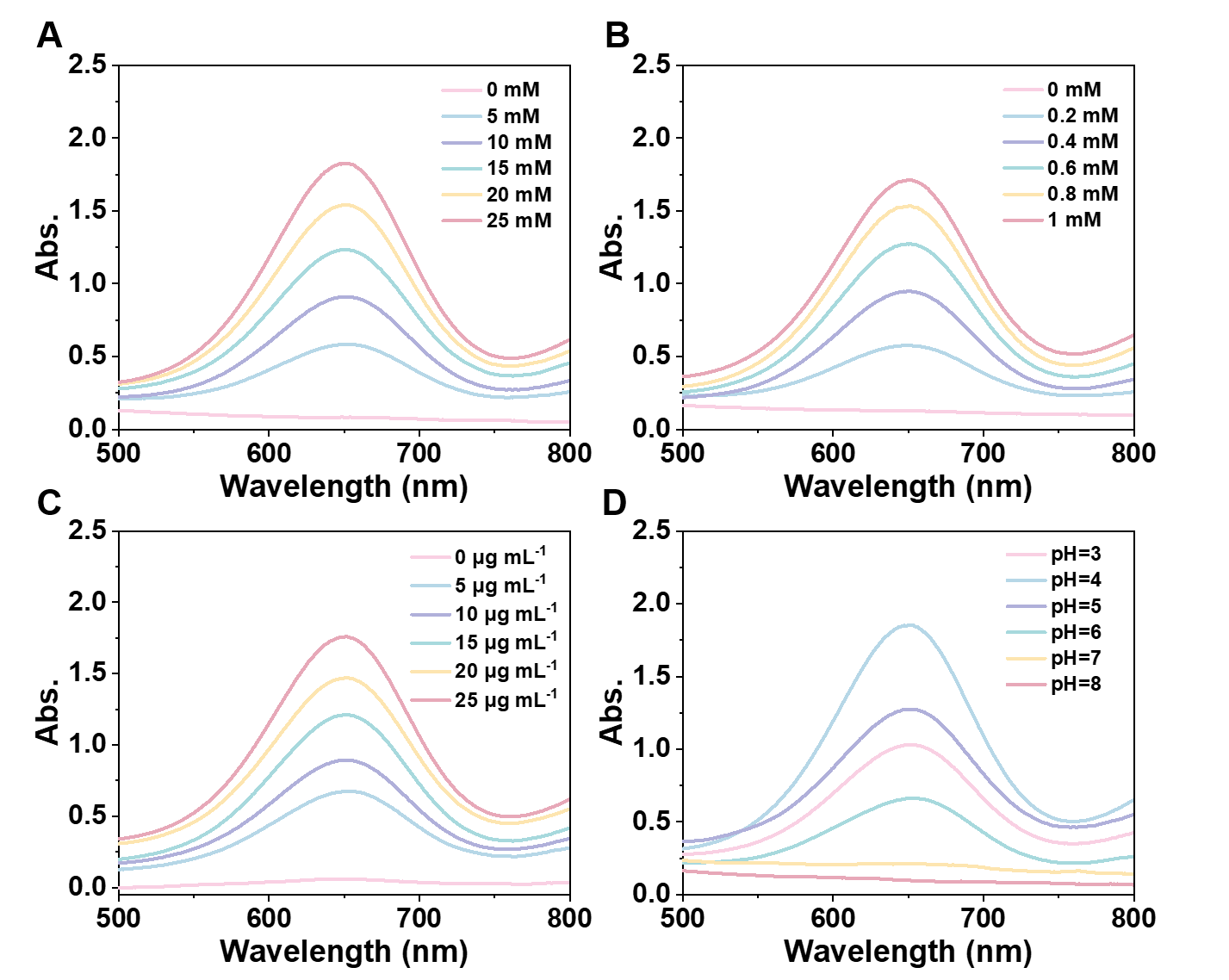


**Figure S6.** The capability of Cu_14_ to catalyze •OH generation from H_2_O_2_ under varying conditions, including (A) H_2_O_2_ concentration, (B) TMB concentration, (C) Cu_14_ concentration, and (D) different pH.


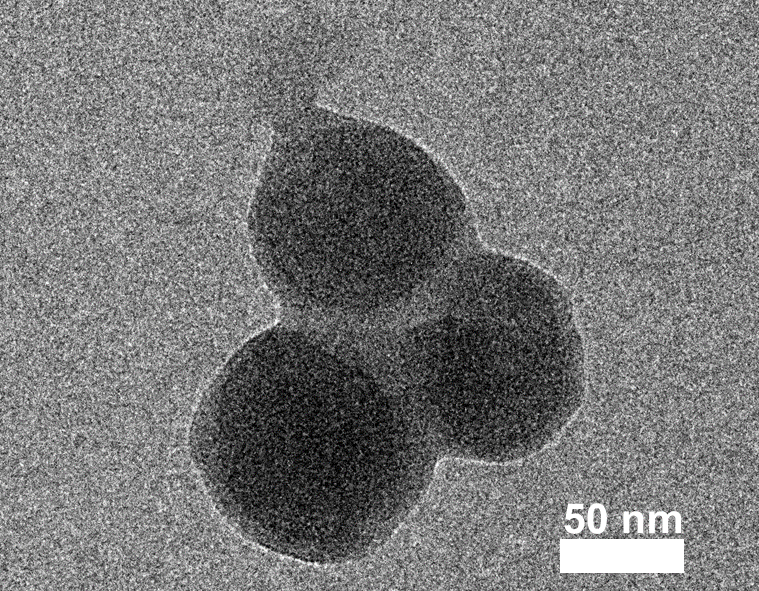


**Figure S7.** The TEM image of Cu_14_@CM.

**
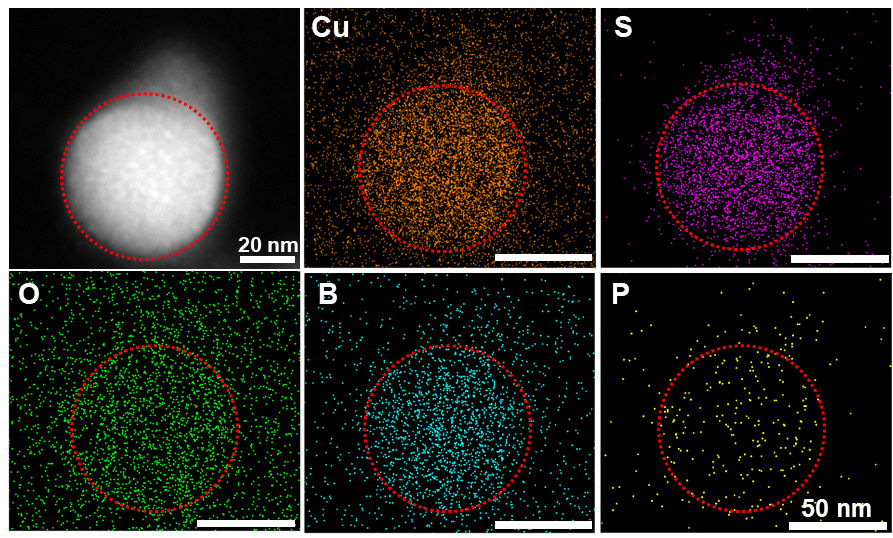
**

**Figure S8.** Dark-field TEM image and the elemental mappings of Cu_14_@CM.

**
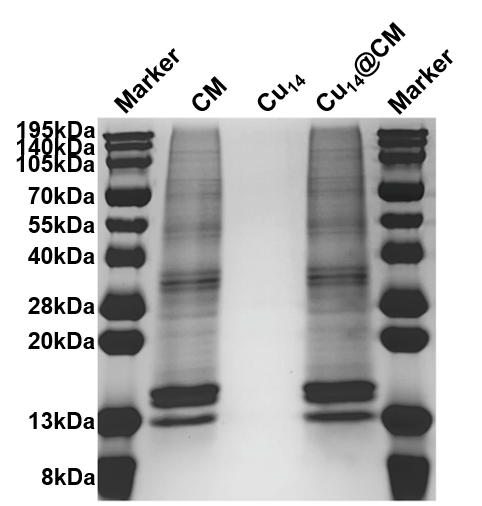
**

**Figure S9.** Protein analysis of 4T1 cells membrane, Cu_14_, and Cu_14_@CM by using SDS-PAGE.


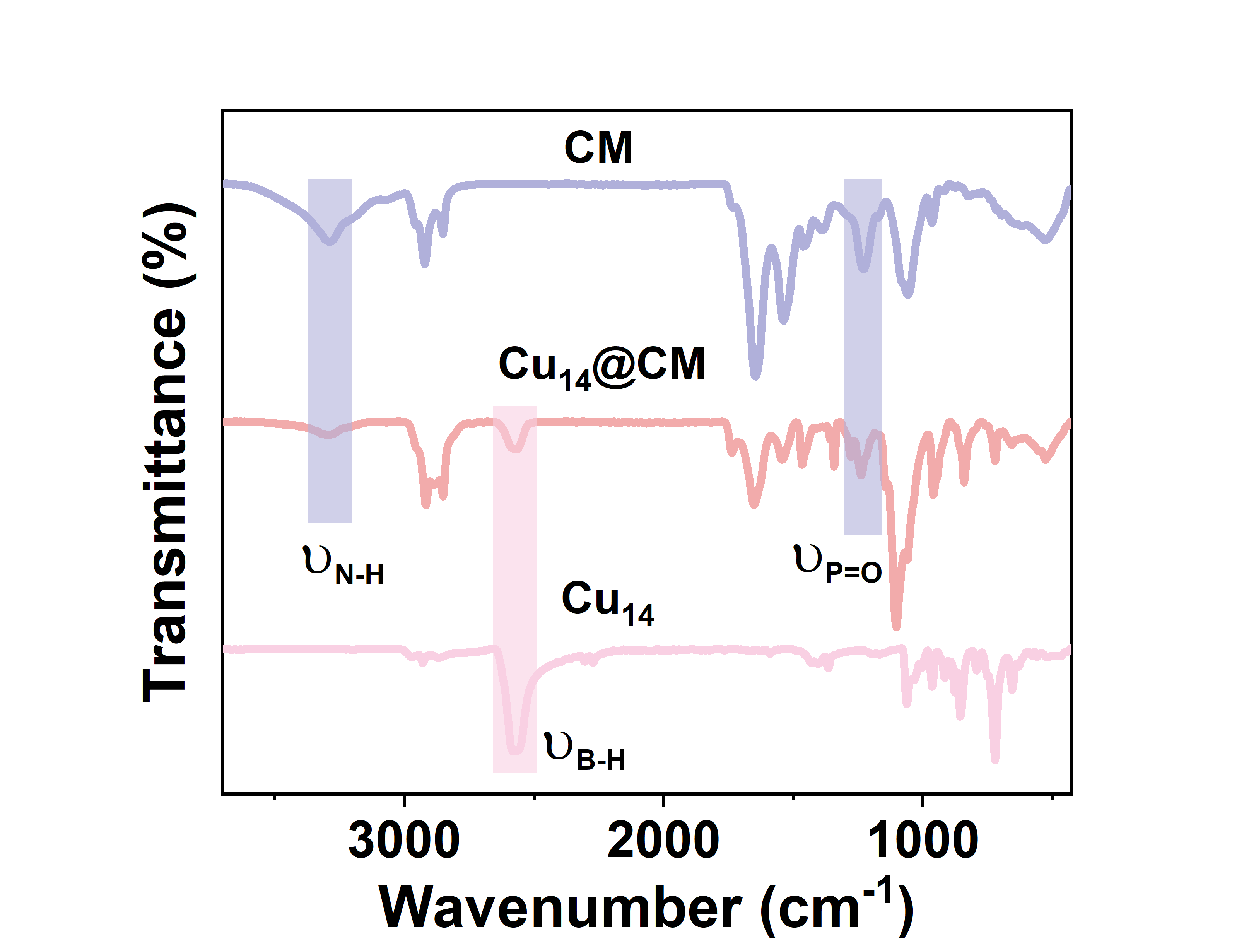


**Figure S10.** FTIR spectra of cell membrane, Cu_14_ cluster and Cu_14_@CM.


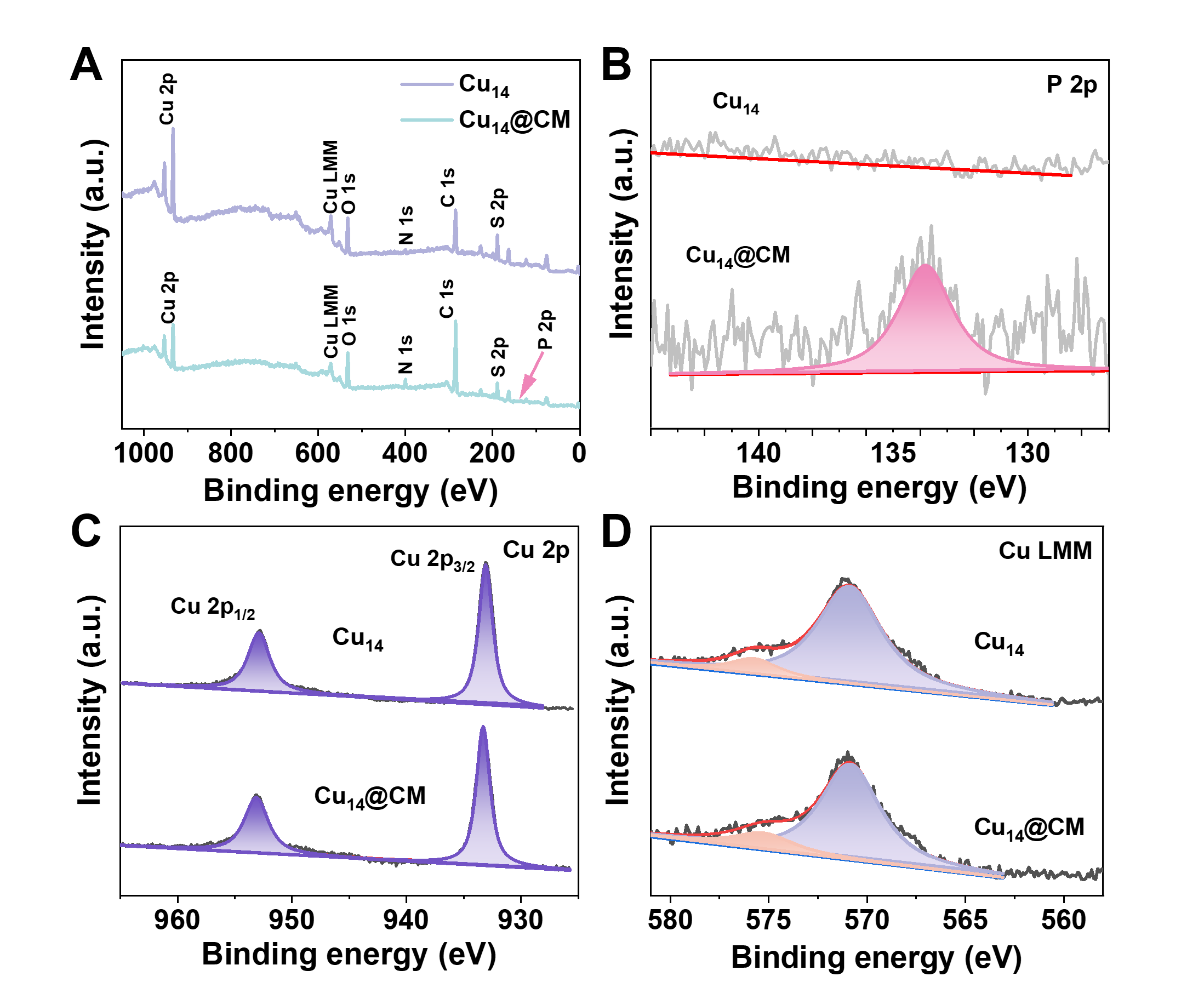


**Figure S11.** (A) XPS survey, (B) P 2p, (C) Cu 2p, and (D) Cu LMM spectra of Cu_14_ and Cu_14_@CM.


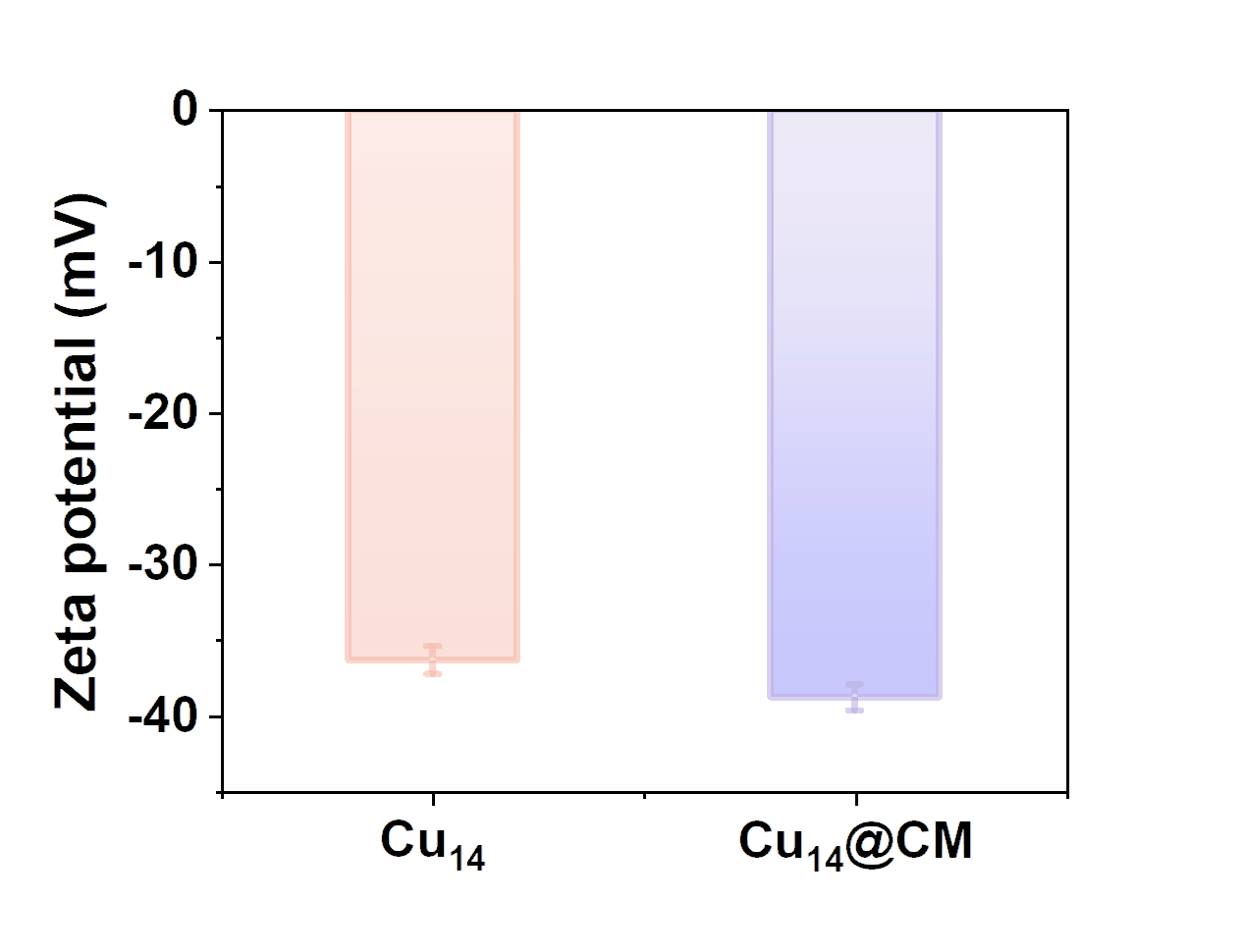


**Figure S12.** Zeta potential of Cu_14_ and Cu_14_@CM. Data are presented as mean ± s.d. from 3 independent biological replicates.


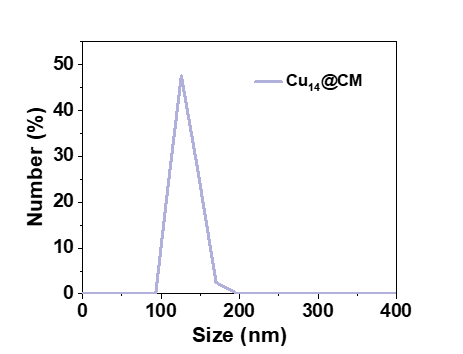


**Figure S13.** The hydrodynamic diameter of Cu_14_@CM.


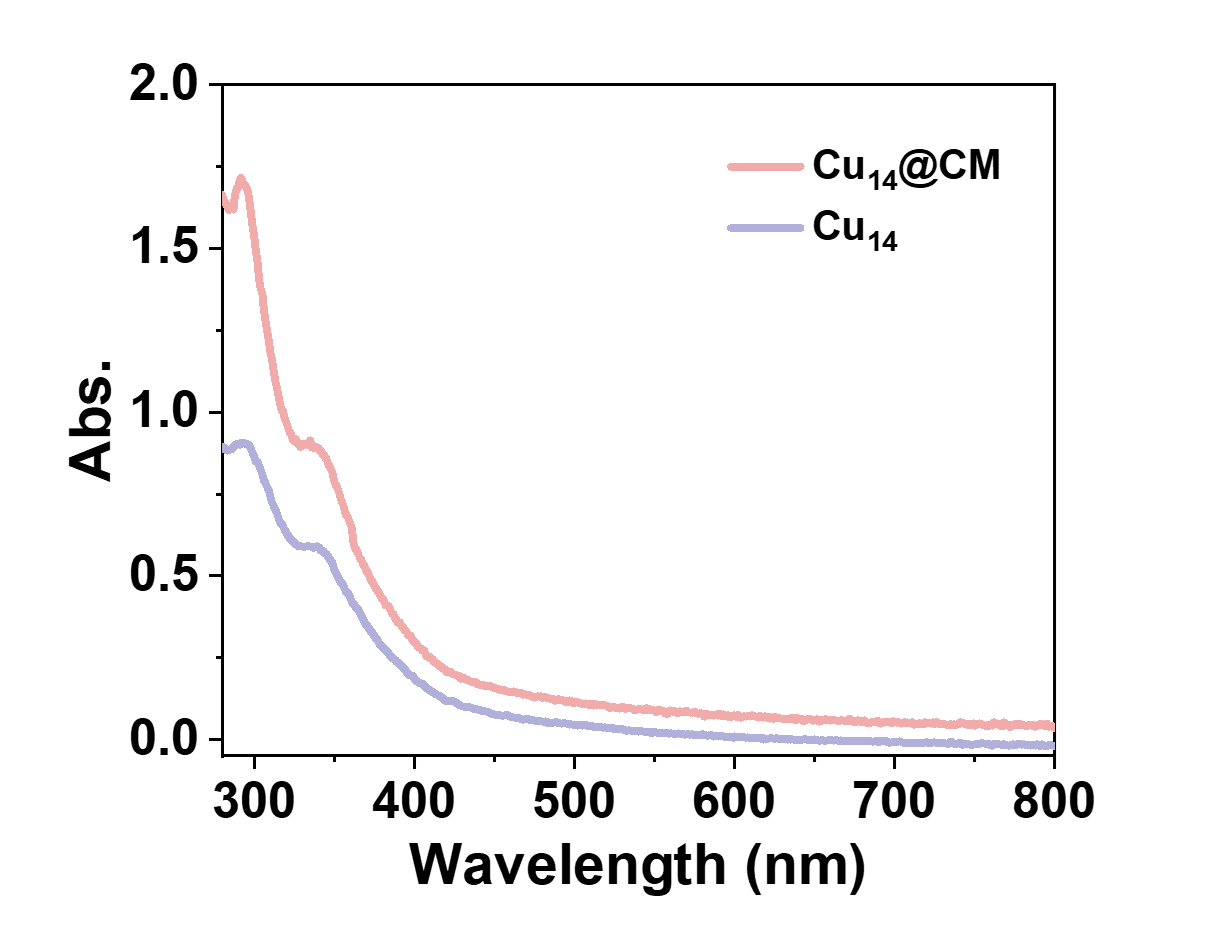


**Figure S14.** UV−Vis absorption spectra of Cu_14_ and Cu_14_@CM.


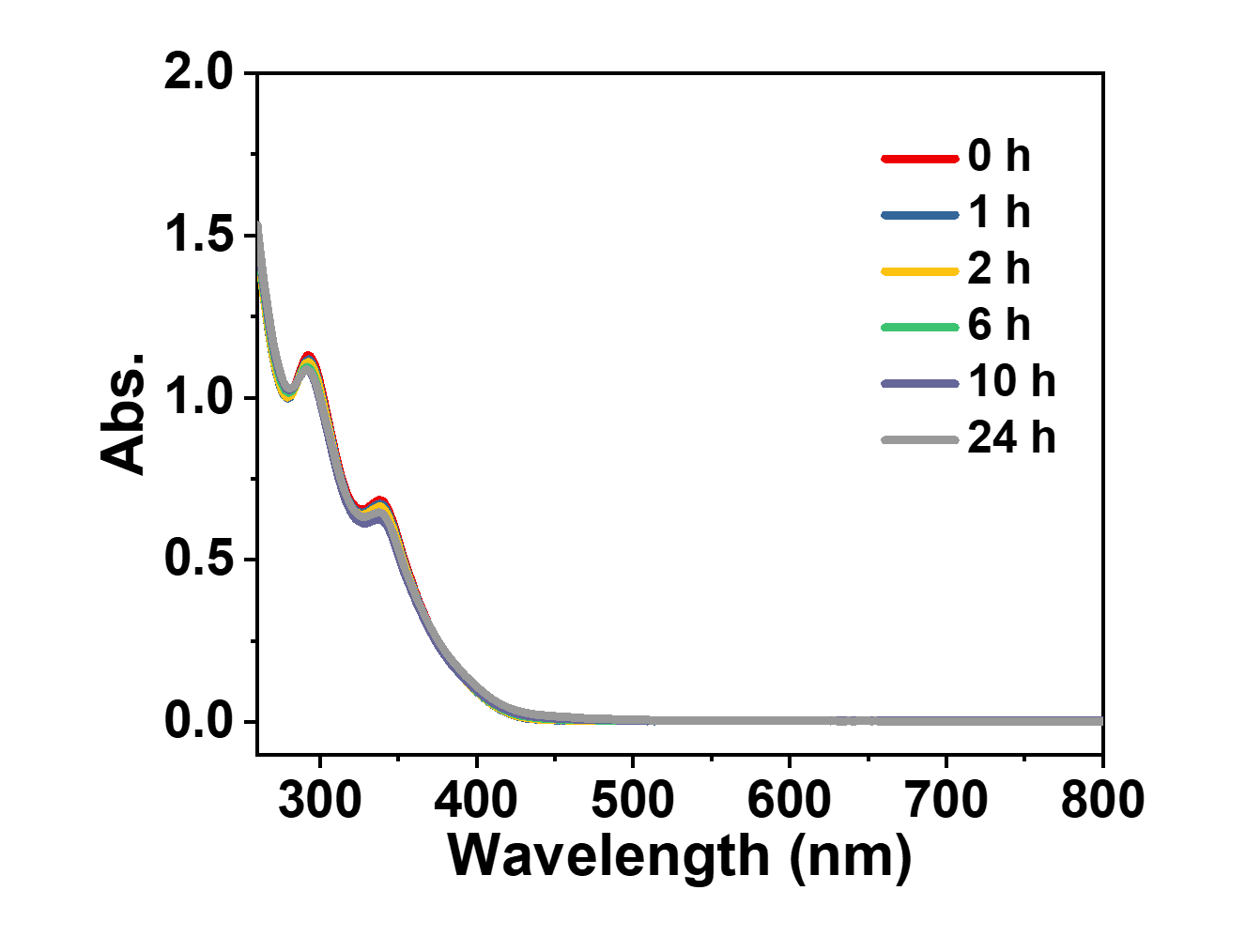


**Figure S15.** The UV-Vis absorption spectra of Cu_14_ after incubating in PBS for different time.


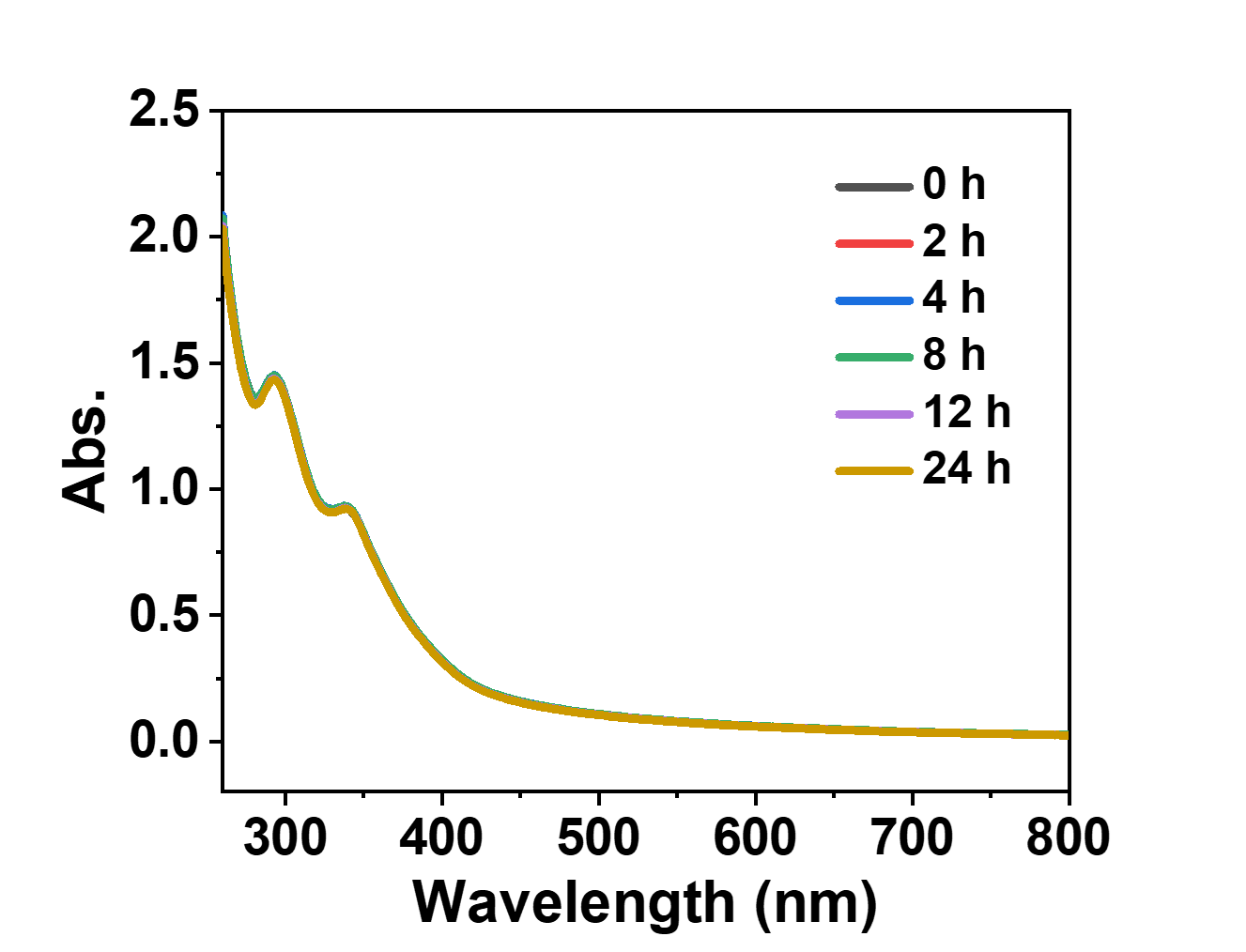


**Figure S16.** The UV-Vis absorption spectra of Cu_14_@CM after incubating in PBS for different time.


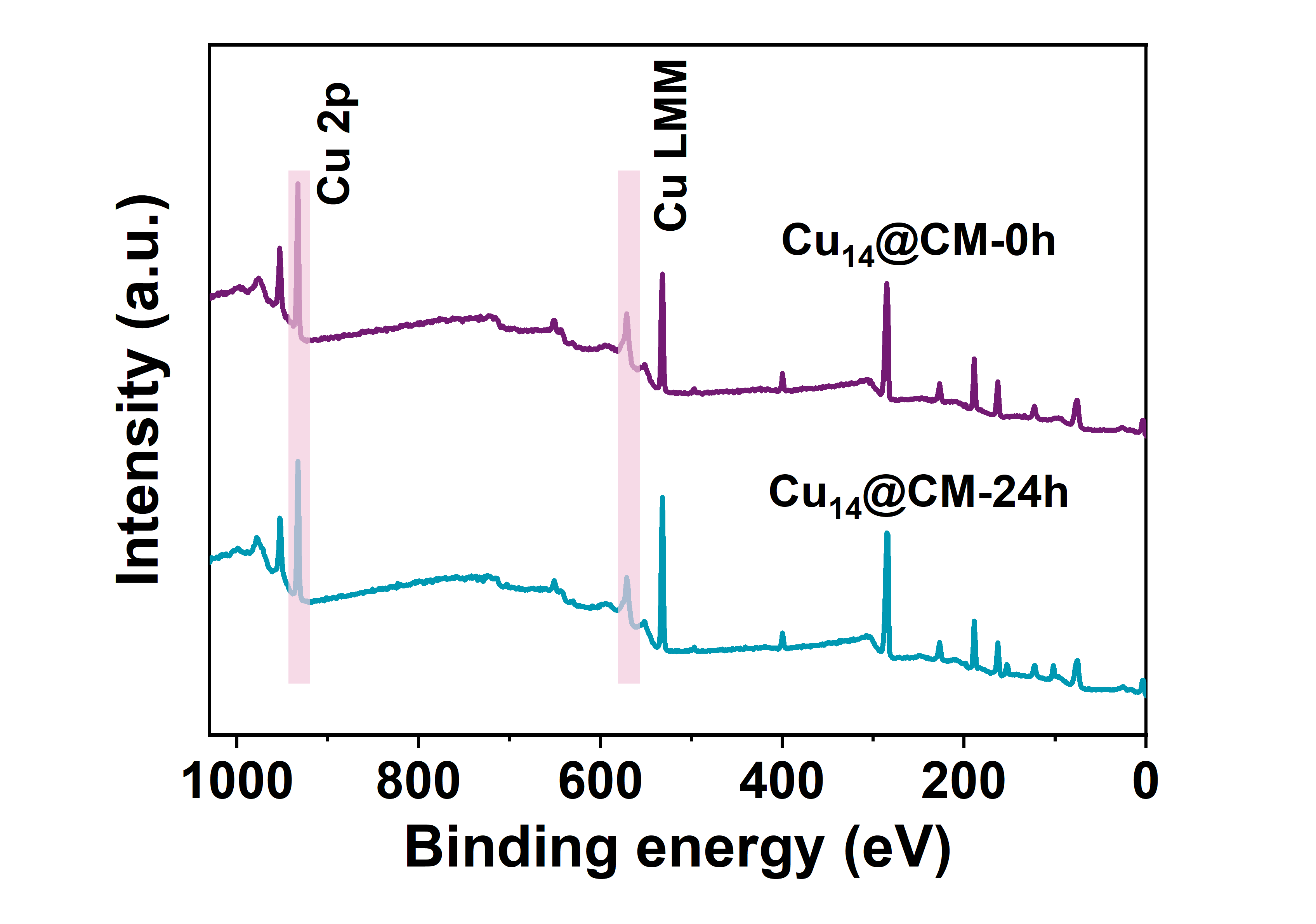


**Figure S17**. The XPS survey spectra of Cu_14_@CM before and after incubating in PBS for 24 h.


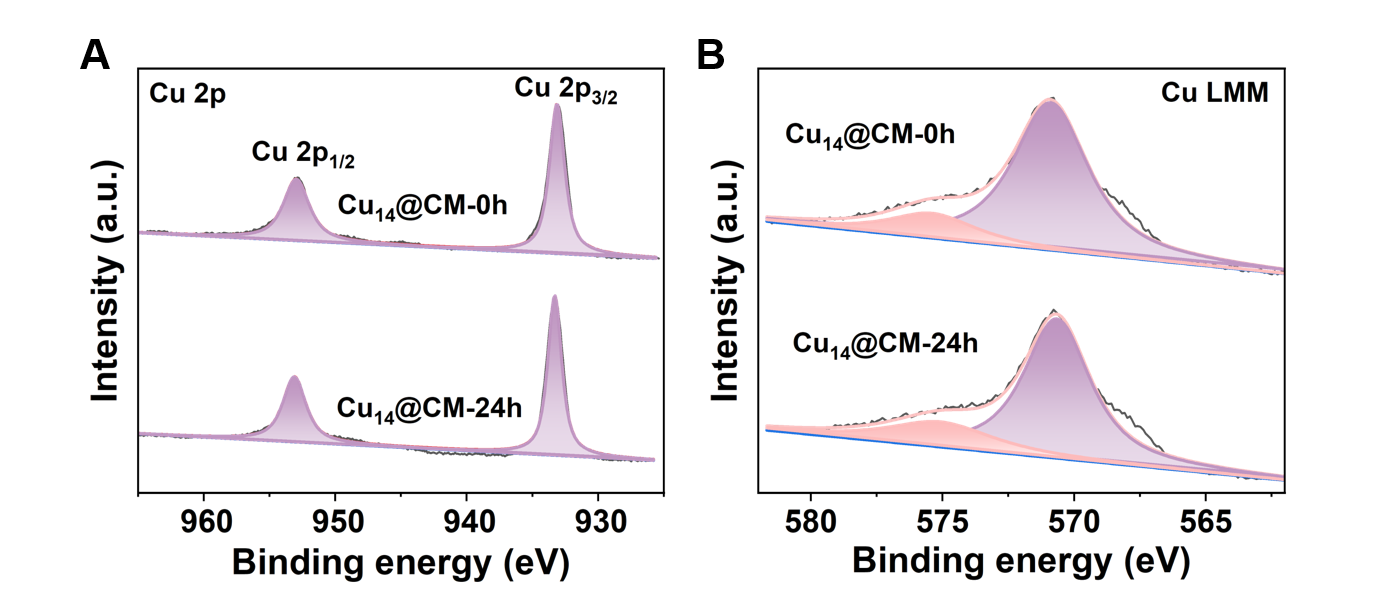


**Figure S18**. (A) The Cu 2p and (B) Cu LMM spectra of Cu_14_@CM before and after incubating in PBS for 24 h.


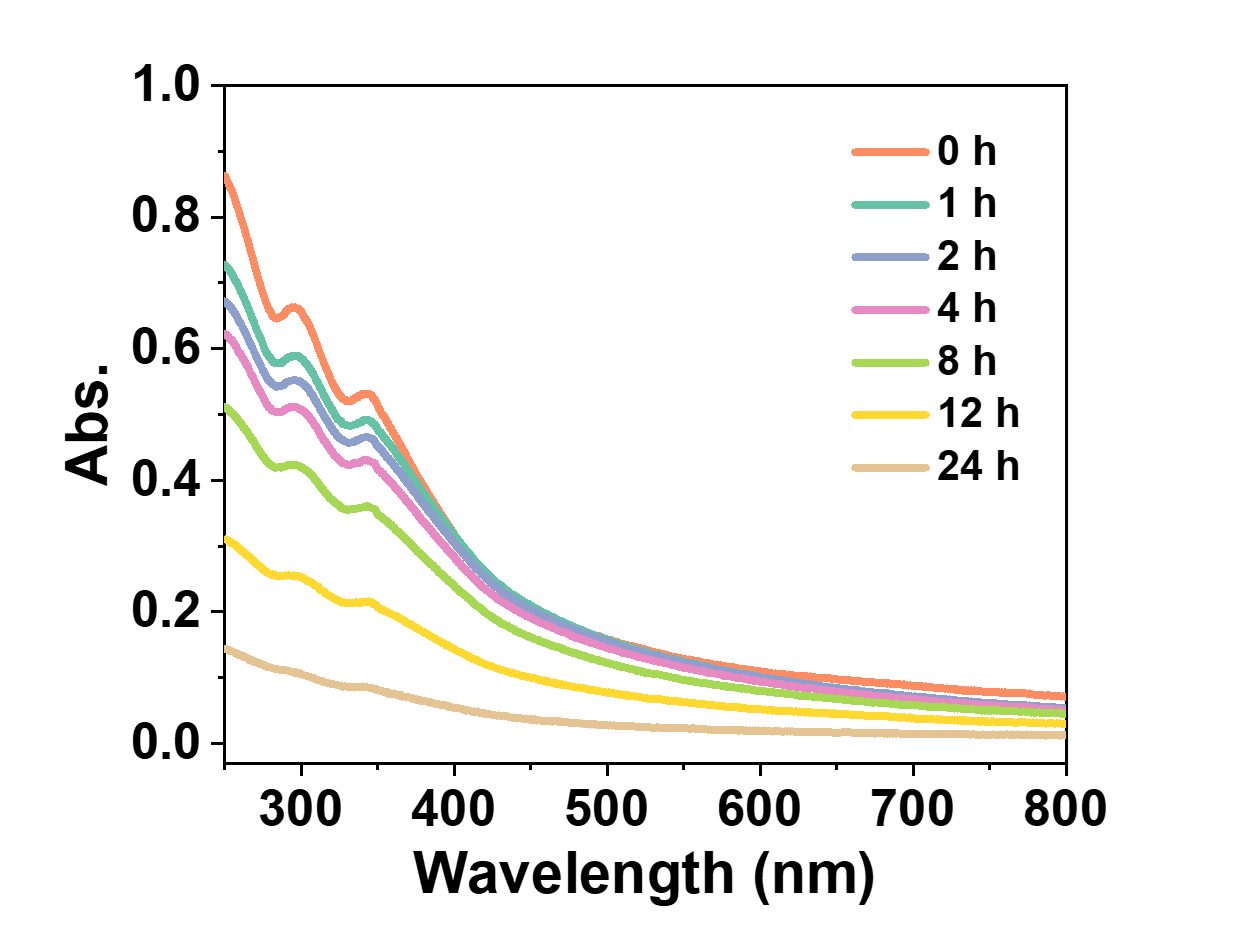


**Figure S19.** The UV-Vis absorption spectra of Cu_14_@CM in PBS at pH 4.


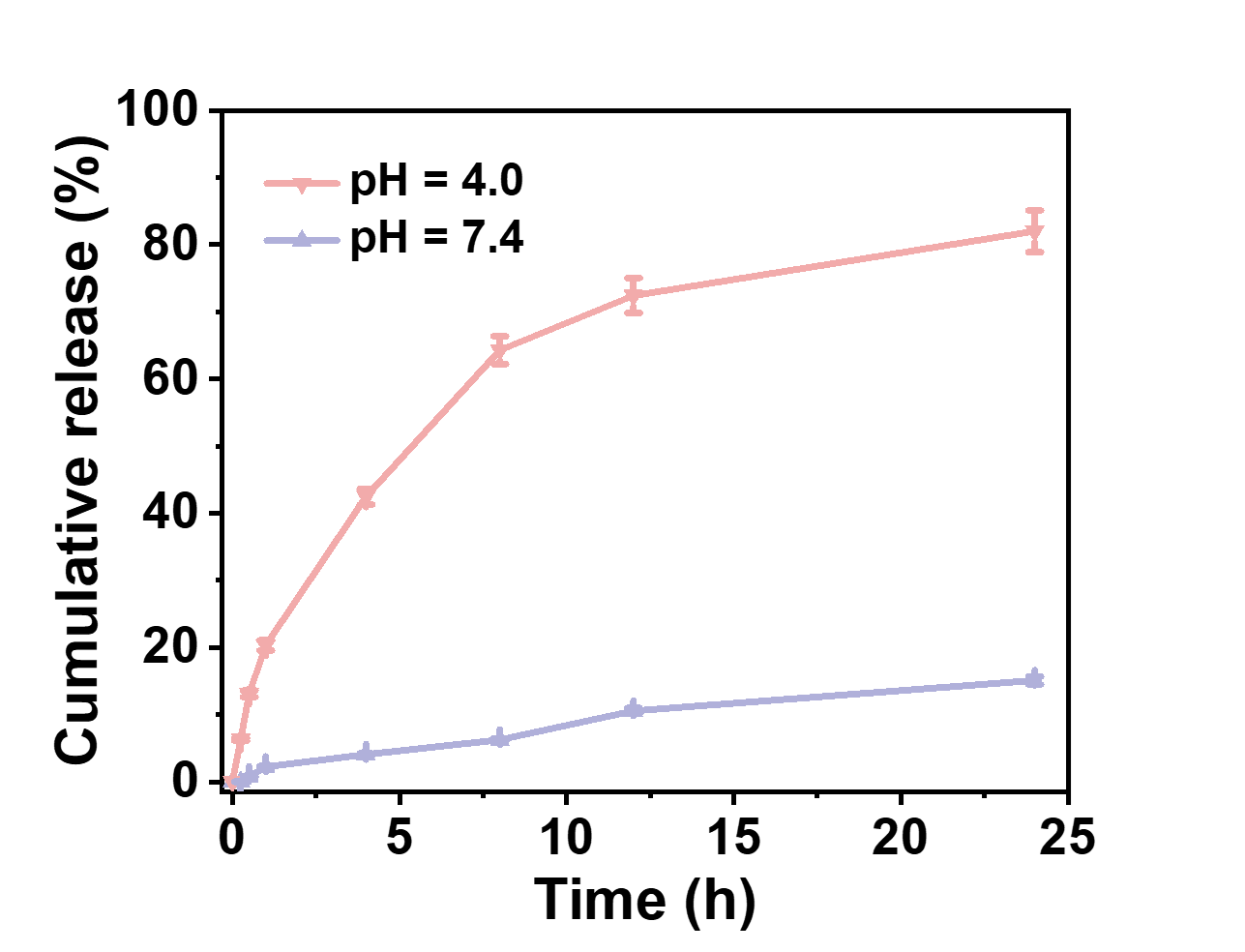


**Figure S20.** The cumulative release of Cu ion under different pH conditions. Data are presented as mean ± s.d. from 3 independent biological replicates.


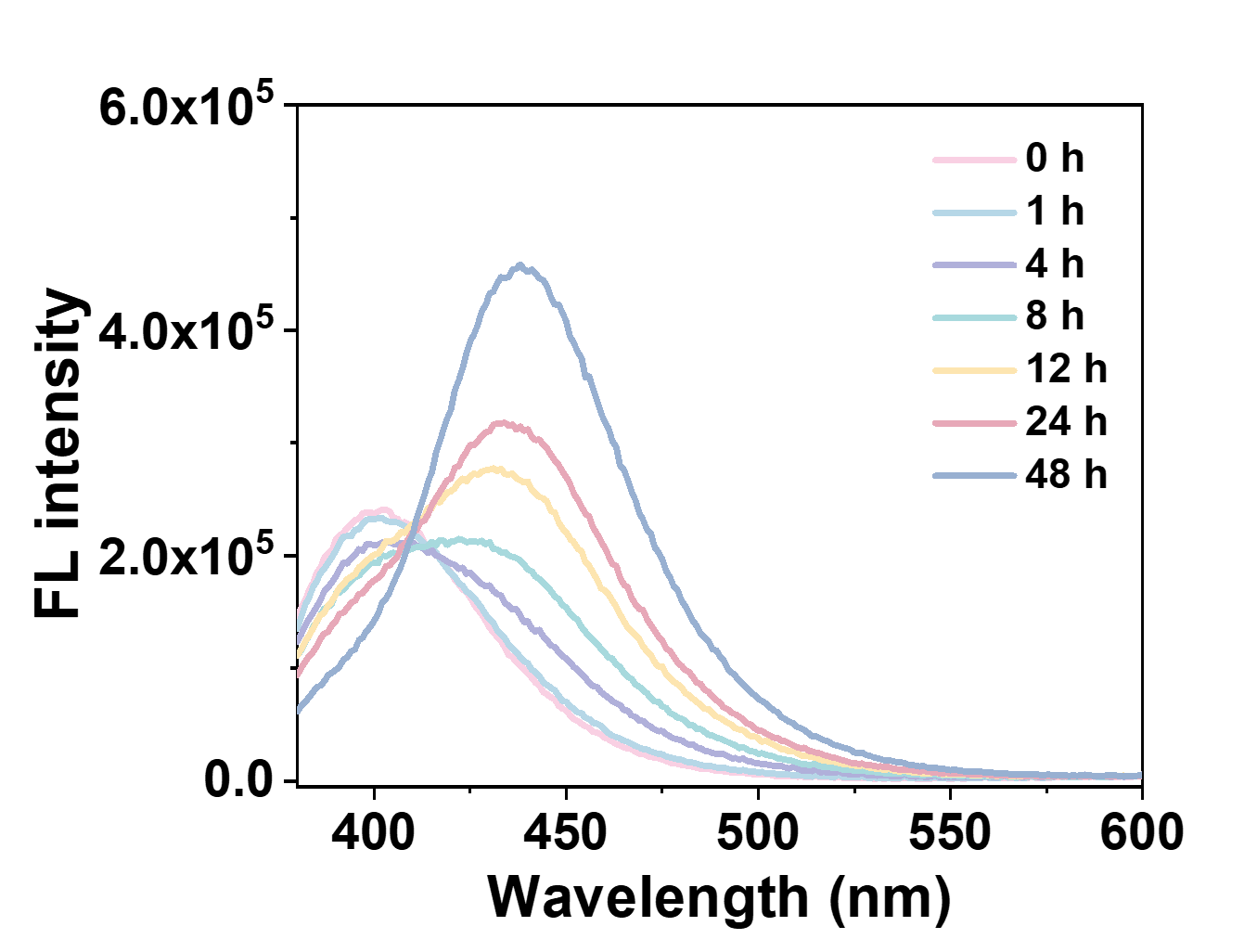


**Figure S21.** The catalytic bond cleavage reaction mediated by Cu_14_@CM in PBS with a pH of 4.


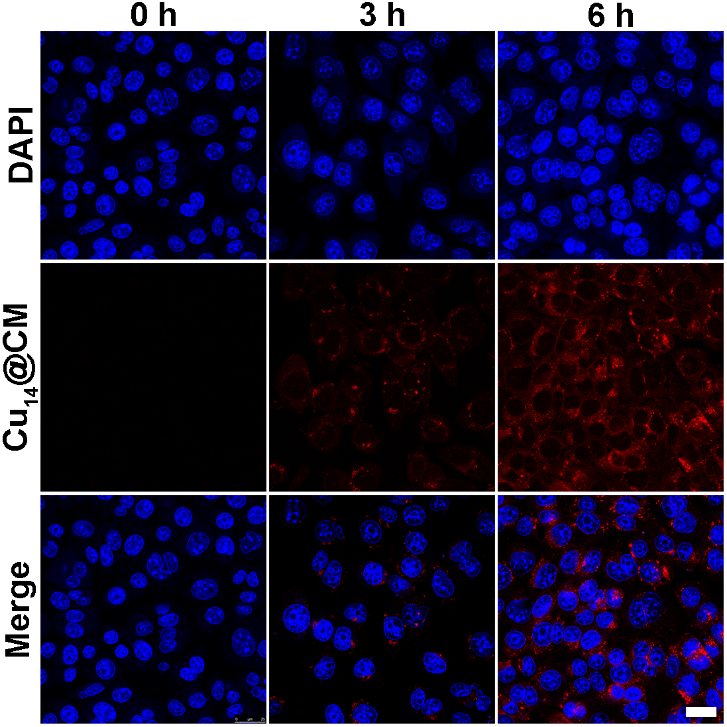


**Figure S22.** The CLSM images of Cu_14_@CM incubated with 4T1 cells for different time. Scale bar is 25 μm.


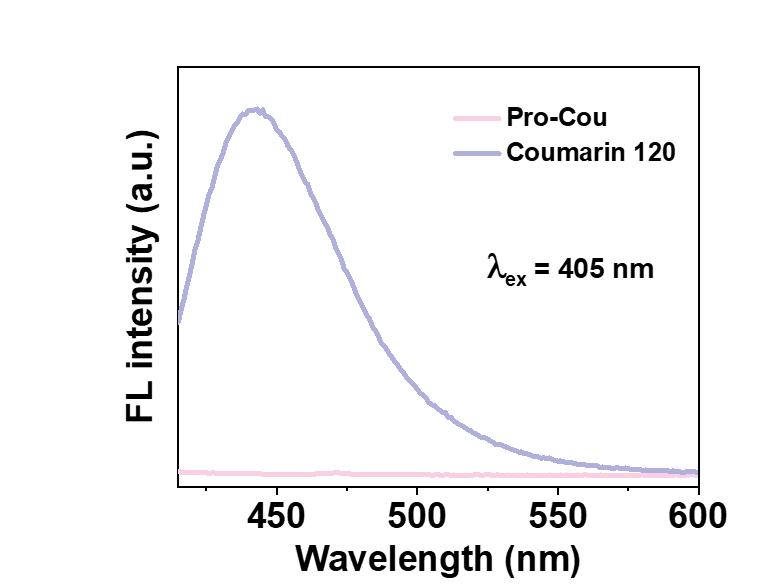


**Figure S23.** The emission spectra of Pro-Cou and Coumarin 120 under a 405 nm excitation wavelength.


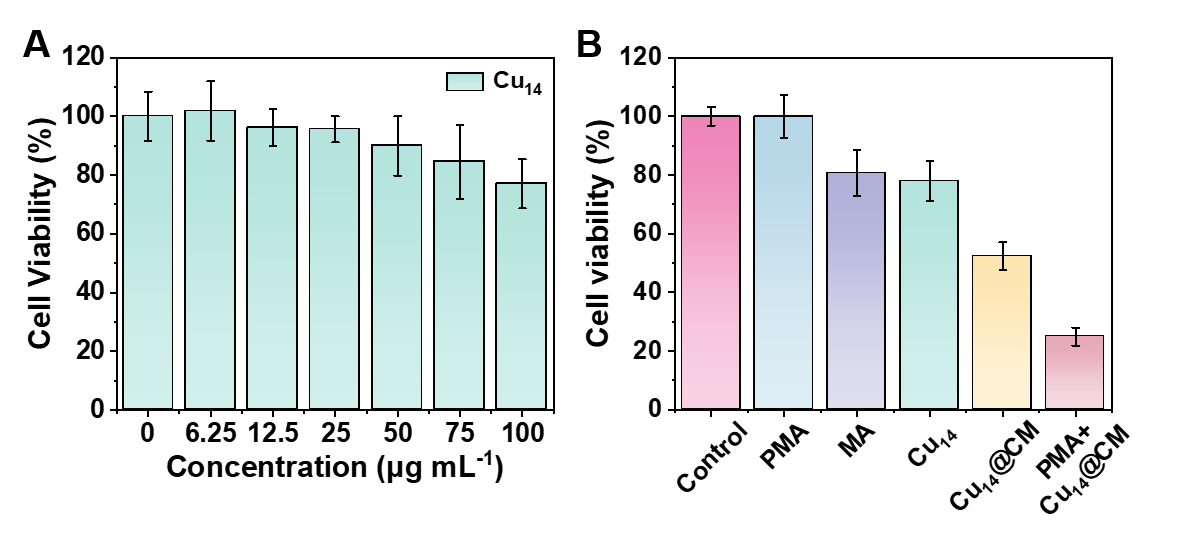


**Figure S24.** Cytotoxic effects of (A) free Cu_14_ without membrane coating and (B) different treatments in 4T1 cells. Data are presented as mean ± s.d. from 5 independent biological replicates.


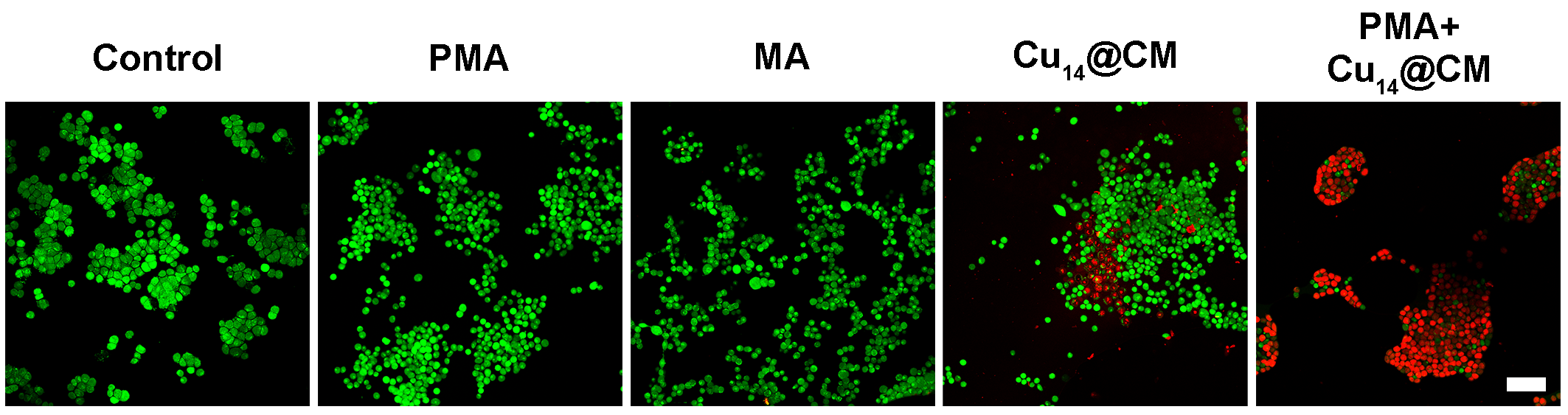


**Figure S25.** The live/dead staining of 4T1 cells after different treatments. Scale bar, 75 μm.


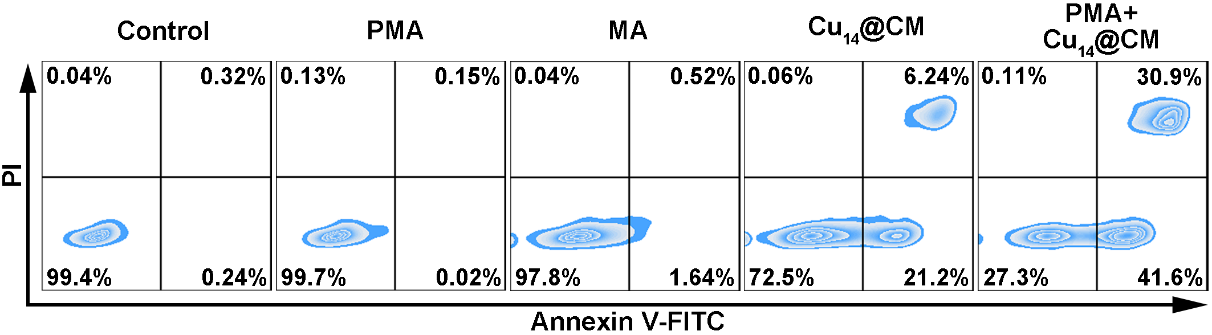


**Figure S26.** The flow cytometry analysis of 4T1 cells after different treatments.


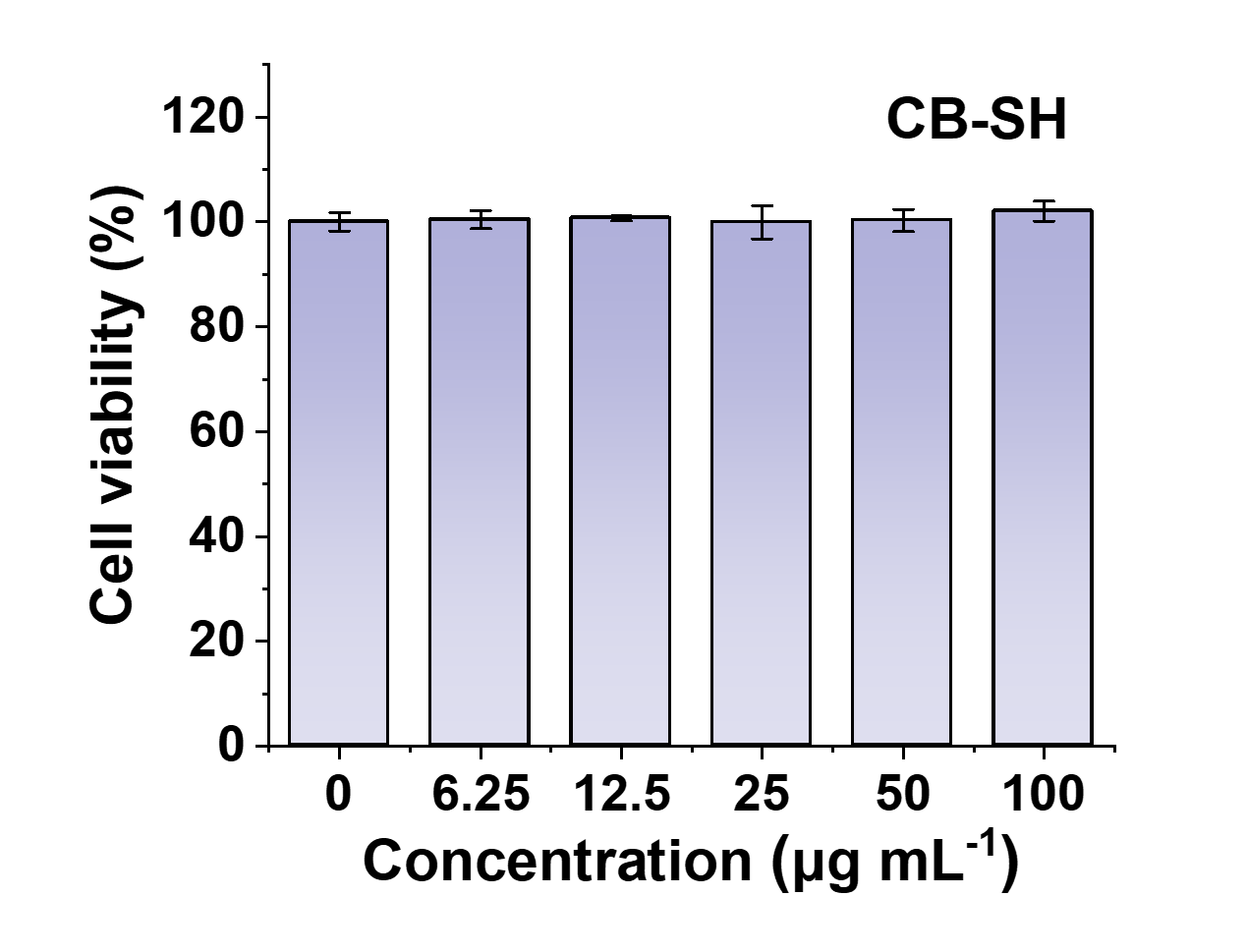


**Figure S27.** Evaluation of cytotoxic effects induced by CB-SH in 4T1 cells. Data are presented as mean ± s.d. from 3 independent biological replicates.


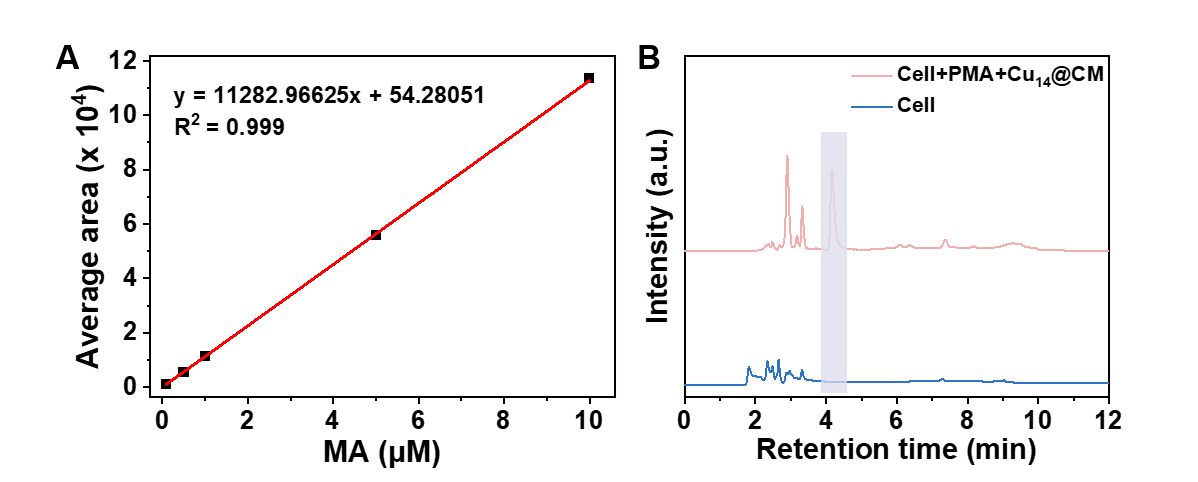


**Figure S28**. (A) The HPLC standard curves of MA. Data are presented as mean ± s.d. from 3 independent biological replicates. (B) HPLC analysis of cellular extracts after treatment with PMA+Cu_14_@CM.


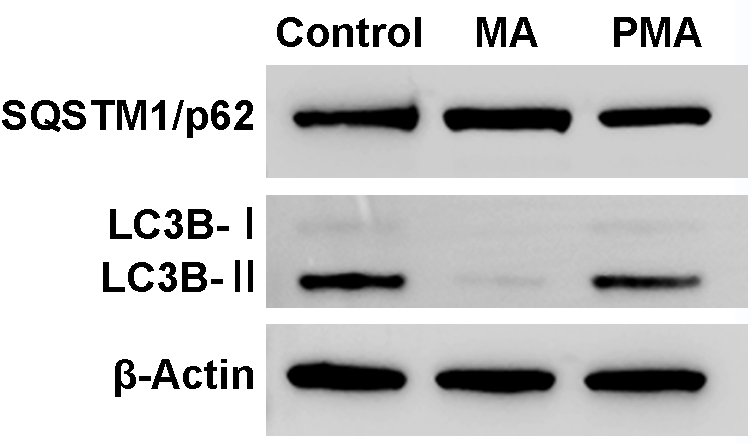


**Figure S29.** Western blot results of the autophagy-related proteins LC3 and p62 in 4T1 cells after treating with MA and PMA.


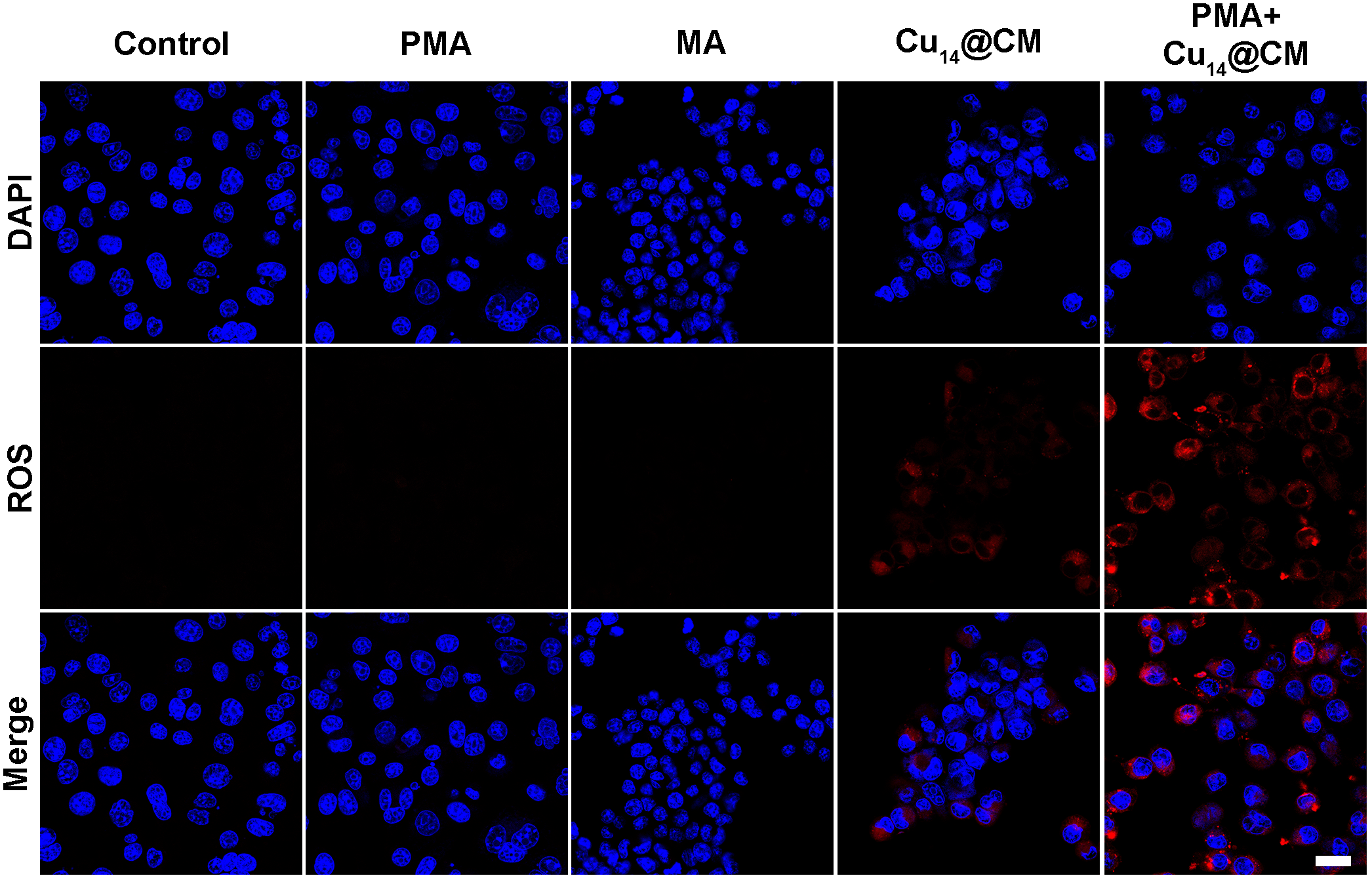


**Figure S30.** The CLSM images of intracellular ROS after different treatments. Scale bars, 25 μm.


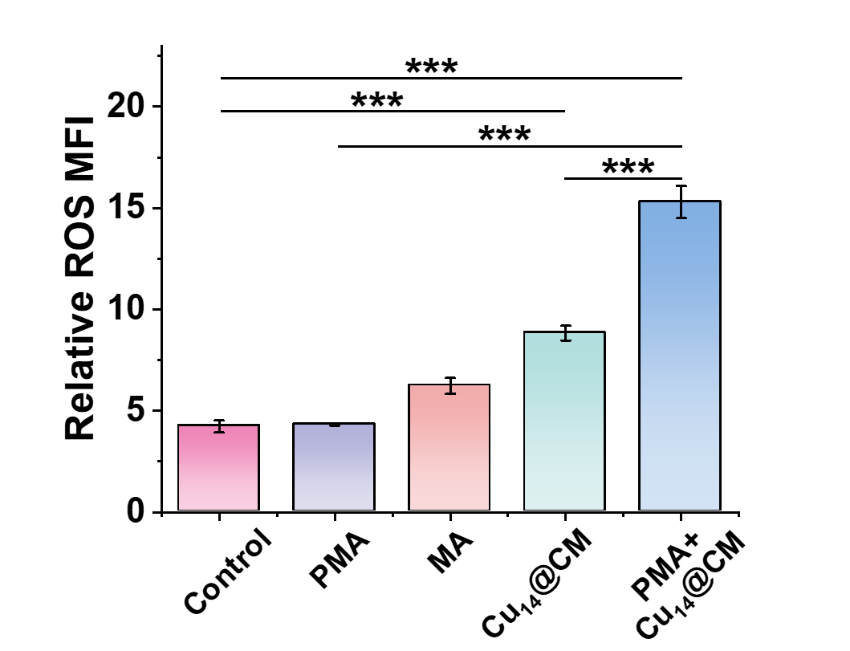


**Figure S31.** Quantitative analysis of intracellular ROS as shown in Figure S31. Data are presented as mean ± s.d. from 3 independent biological replicates. Two-tailed Student's t-test were used for comparisons between two independent groups (**p* < 0.05, ***p* < 0.01, ****p* < 0.001).


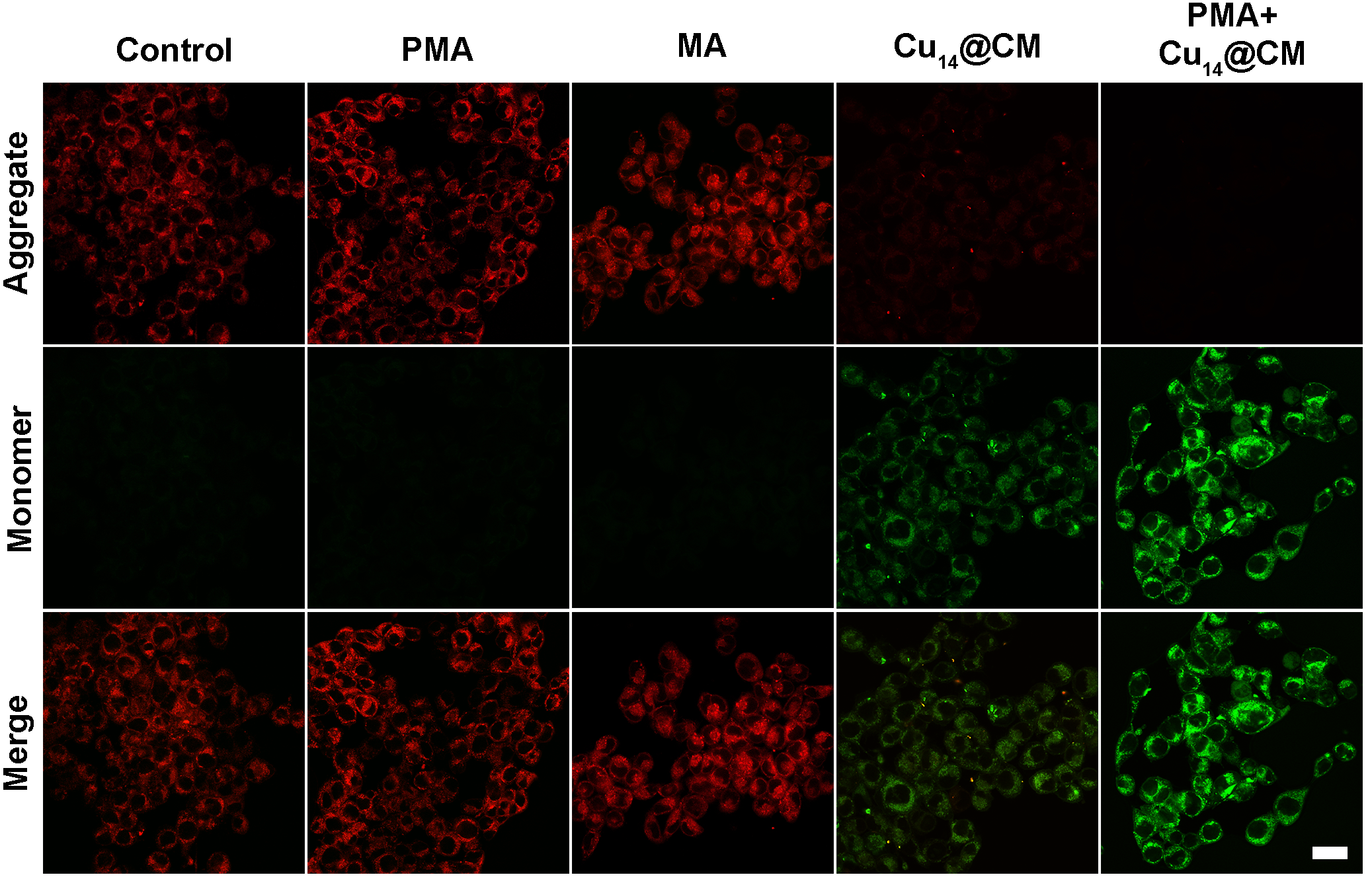


**Figure S32.** JC-1 staining of 4T1 cells in different treatments. Scale bar, 25 μm.


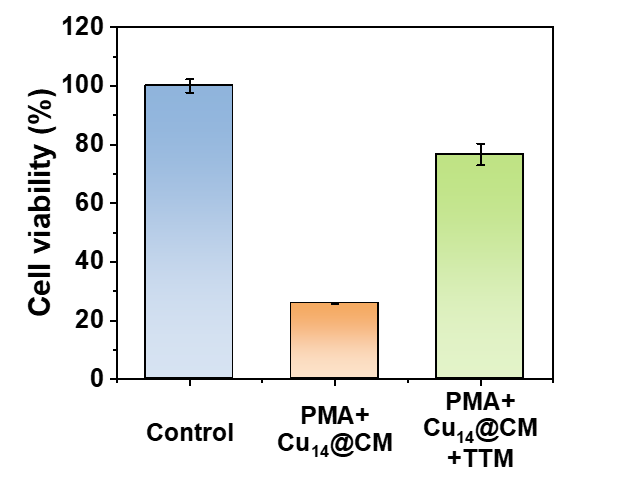


**Figure S33.** The cell viability of PMA+Cu_14_@CM treatment in the presence and absence of TTM. Data are presented as mean ± s.d. from 6 independent biological replicates.


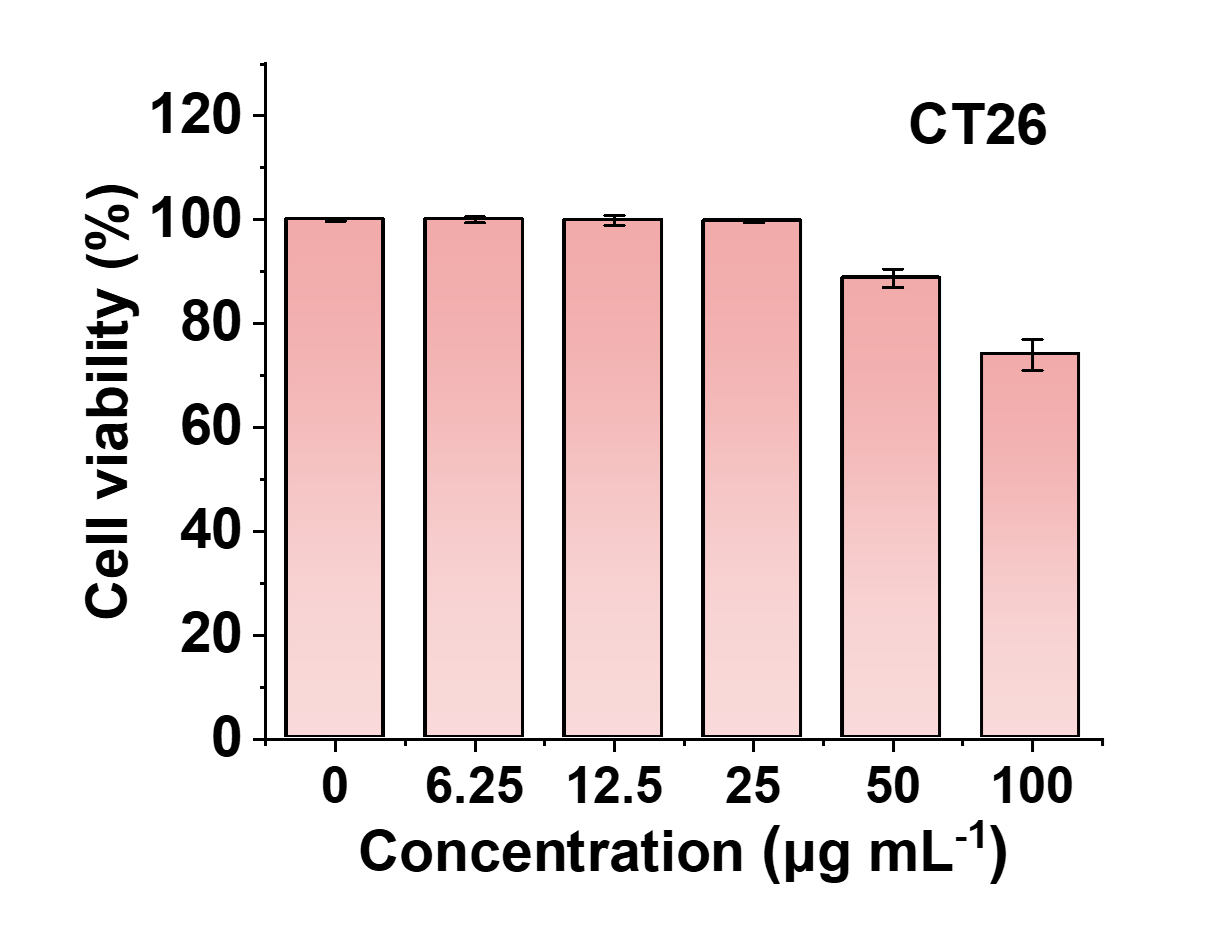


**Figure S34.** Evaluation of cytotoxic effects induced by Cu_14_@CM in CT26 cells. Data are presented as mean ± s.d. from 3 independent biological replicates.


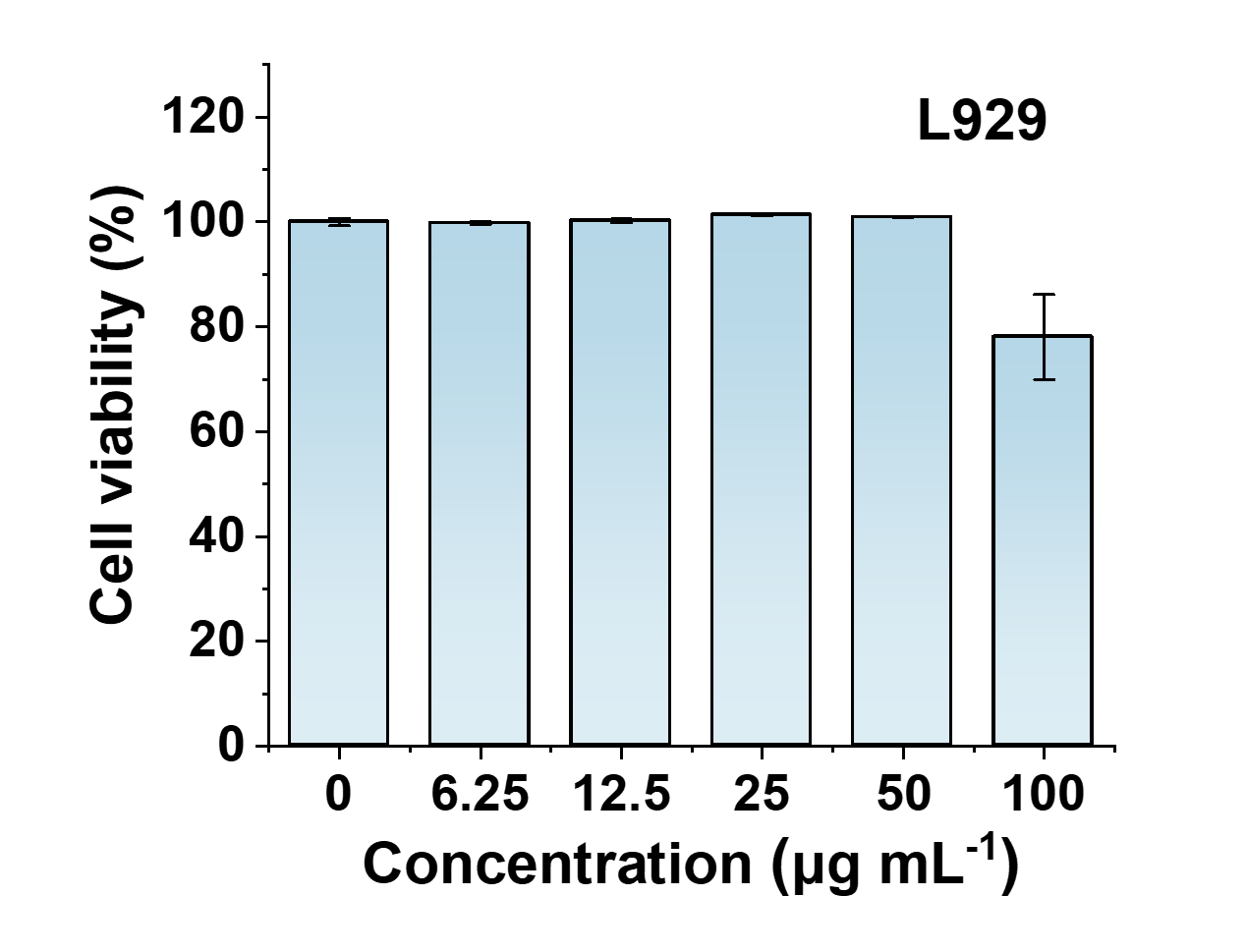


**Figure S35.** Evaluation of cytotoxic effects induced by Cu_14_@CM in L929 cells. Data are presented as mean ± s.d. from 3 independent biological replicates.


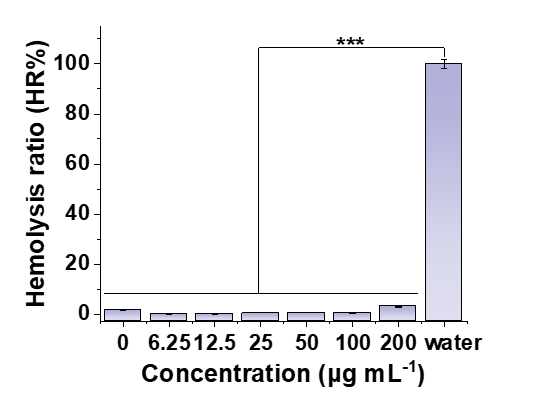


**Figure S36.** The hemolysis rate (HR%) of Cu_14_@CM. Data are presented as mean ± s.d. from 3 independent biological replicates. Two-tailed Student's t-test were used for comparisons between two independent groups (**p* < 0.05, ***p* < 0.01, ****p* < 0.001).


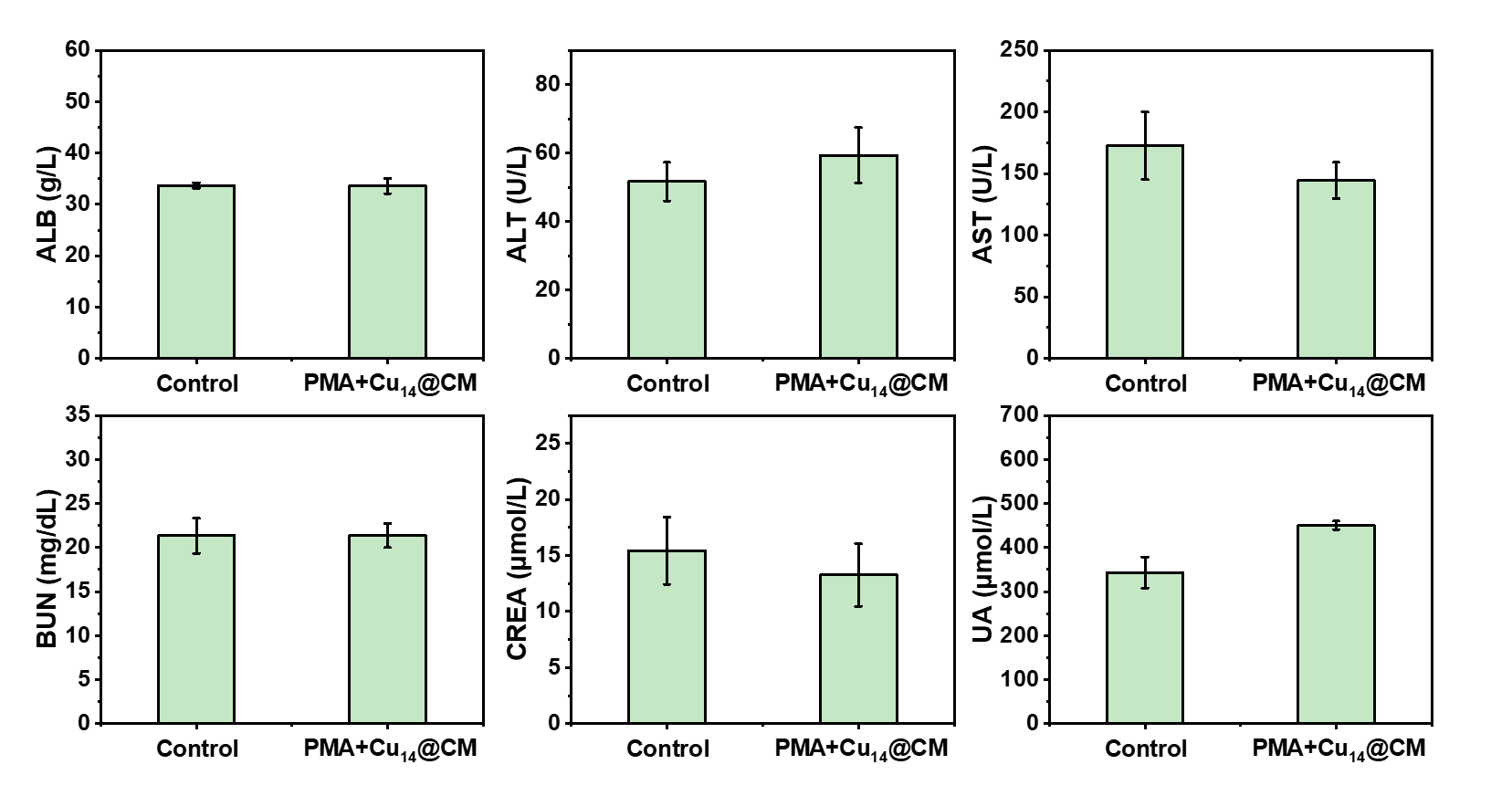


**Figure S37.** Blood biochemical analysis in mice following 14 days of various treatments. ALB, alburnin; ALT, alanine transferase; AST, aspartate transferase; BUN, blood urea nitrogen; CREA, creatinine; UA, urea. Data are presented as mean ± s.d. from 3 independent biological replicates.


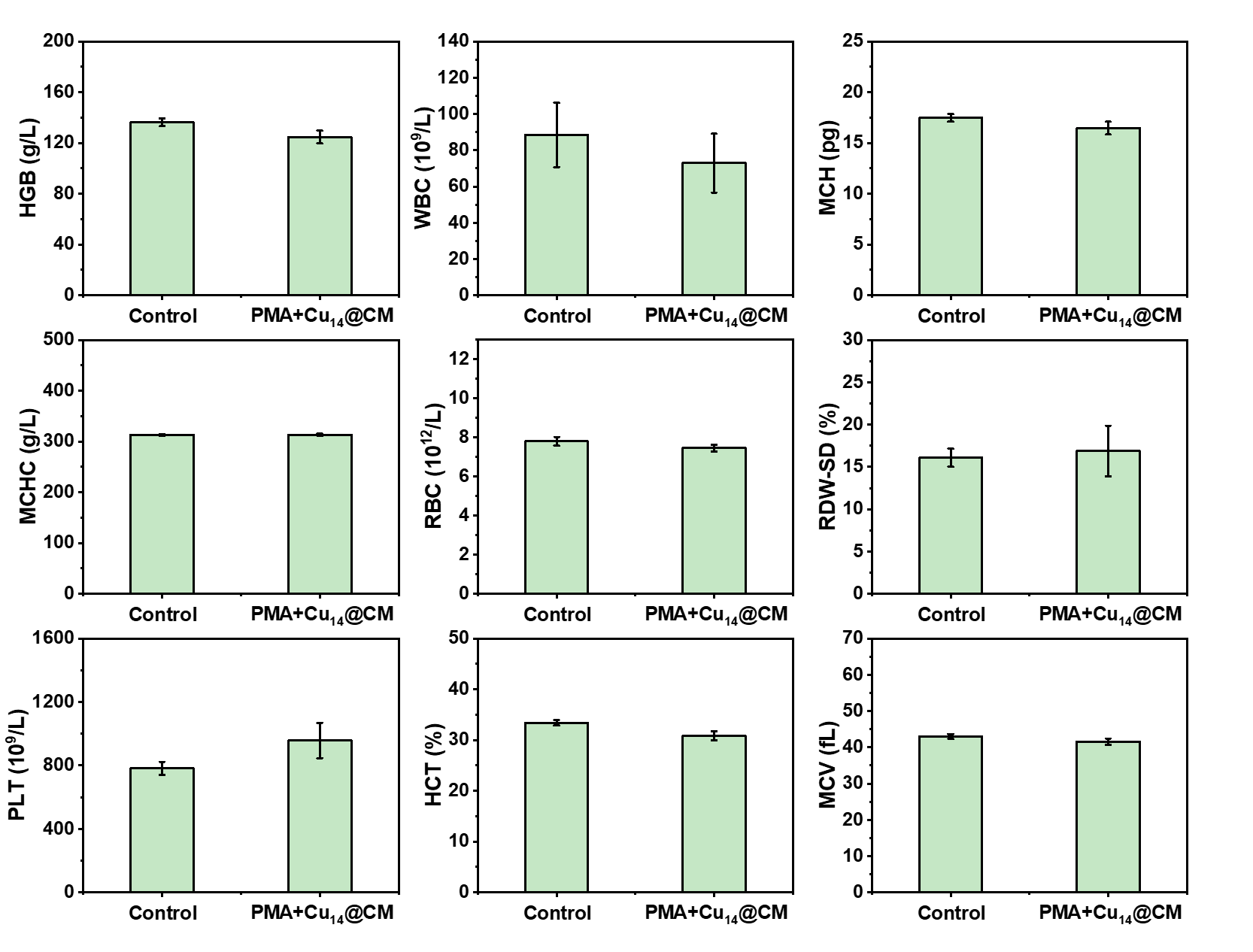


**Figure S38.** Hematological analysis in mice following 14 days of various treatments. HGB, hemoglobin; WBC, white blood cell count; MCH, mean corpuscular hemoglobin content; MCHC, mean corpuscular hemoglobin concentration; RBC, red blood cell count; RDW-SD, red blood cell distribution width; PLT, platelet count; HCT, hematocrit; MCV, mean corpuscular volume. Data are presented as mean ± s.d. from 3 independent biological replicates.


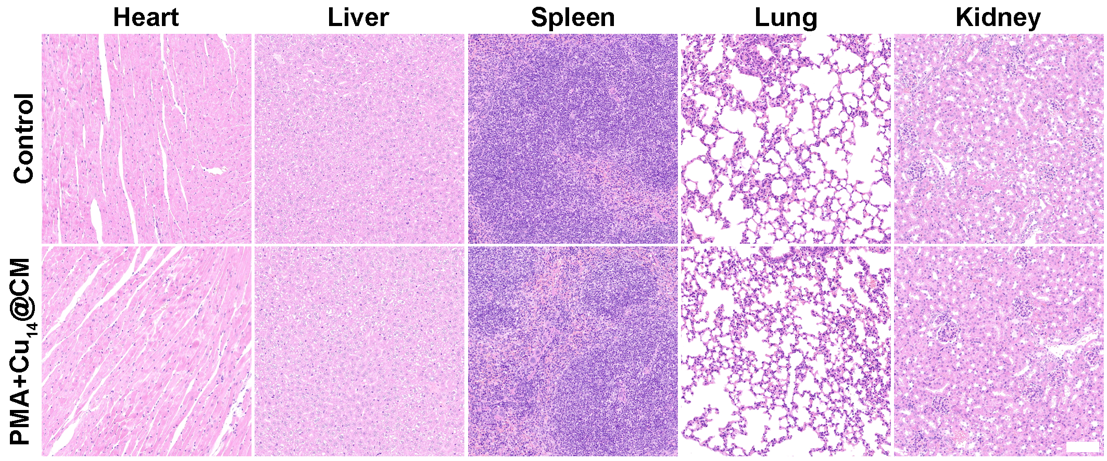


**Figure S39.** H&E images obtained from the major organs of the PBS- or PMA+Cu_14_@CM-treated mice. Scale bar, 100 μm.


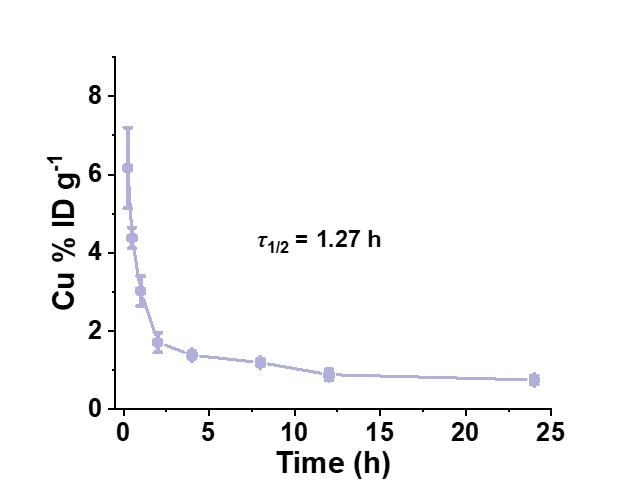


**Figure S40.** Blood circulation curve of Cu_14_@CM determined by measuring the Au concentration at different time points after injection. Data are presented as mean ± s.d. from 3 independent biological replicates.

**
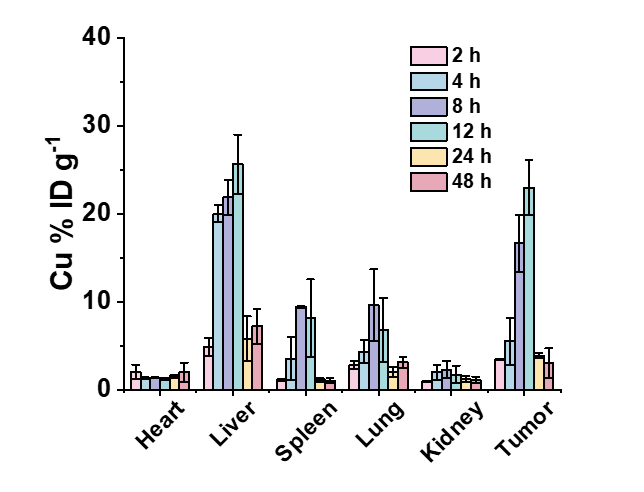
**

**Figure S41.** Biodistribution of Cu_14_@CM at different time after post-injection. Data are presented as mean ± s.d. from 3 independent biological replicates.

**
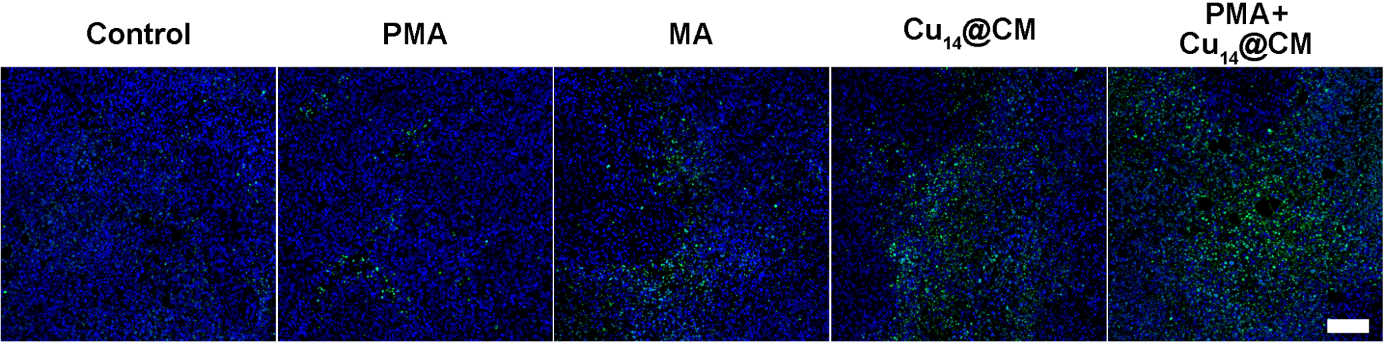
**

**Figure S42.** TUNEL staining of tumor tissue of 4T1 mice with different treatments after the treatment ended. Scale bar, 100 μm.

**
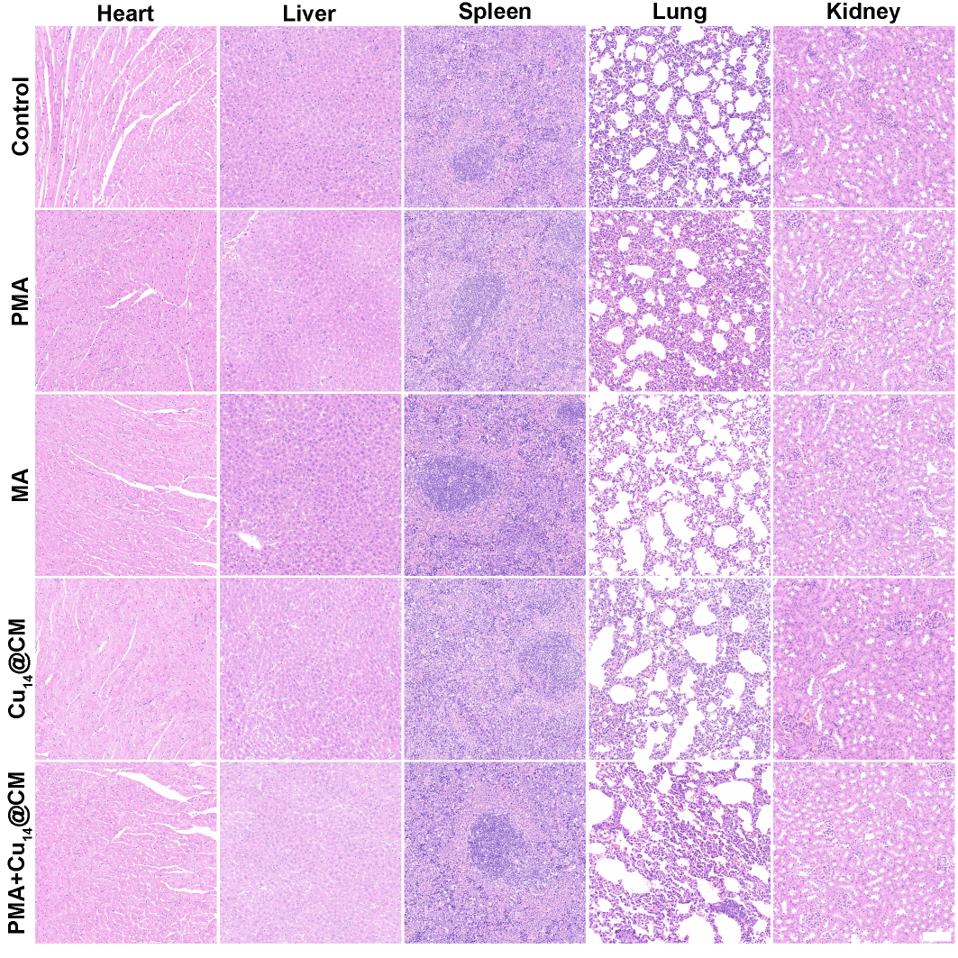
**

**Figure S43.** H&E images of obtained from the major organs of 4T1 mice with different treatments after the treatment ended. Scale bar, 100 μm.


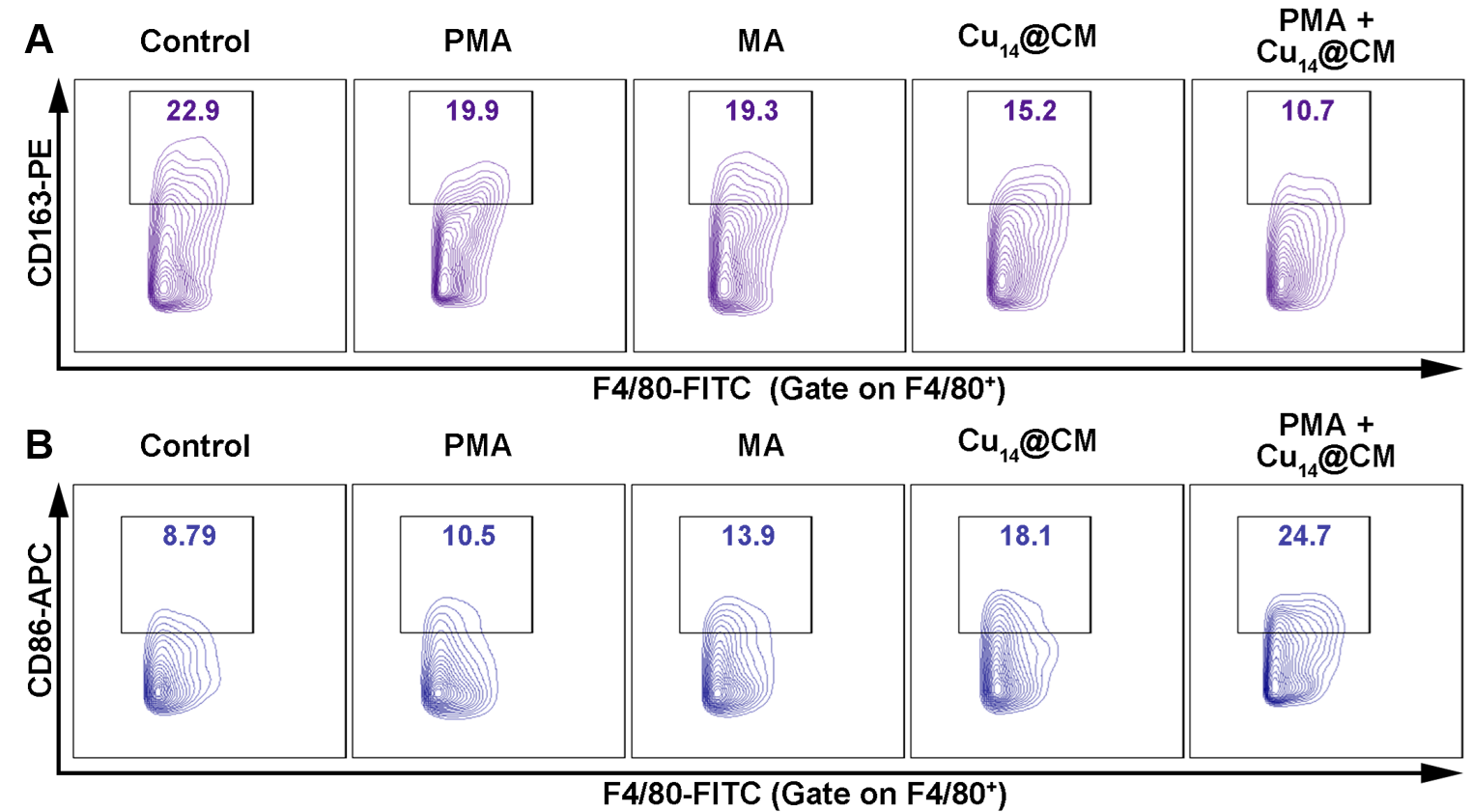


**Figure S44.** Flow cytometric analysis of (A) M2-like macrophages and (B) M1-like macrophages in tumor tissues.


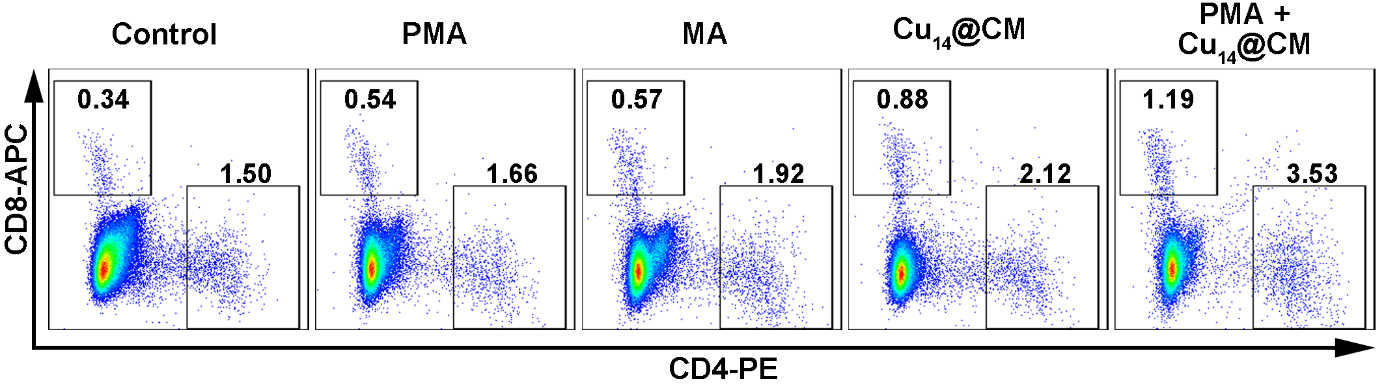


**Figure S45.** The flow cytometry analysis of the percentage of CD4^+^ and CD8^+^ T cells in tumor tissue with different treatments after the treatment ended.


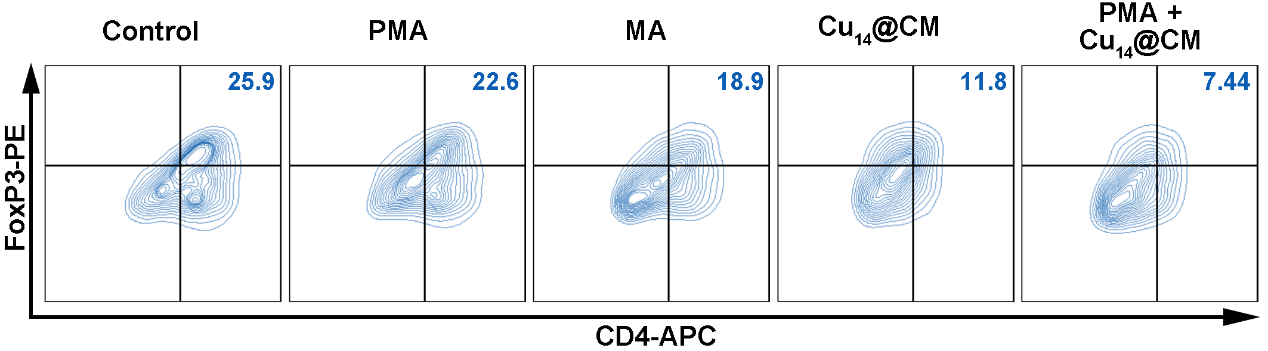


**Figure S46.** Flow cytometric analysis of regulatory T cells in tumor tissues.


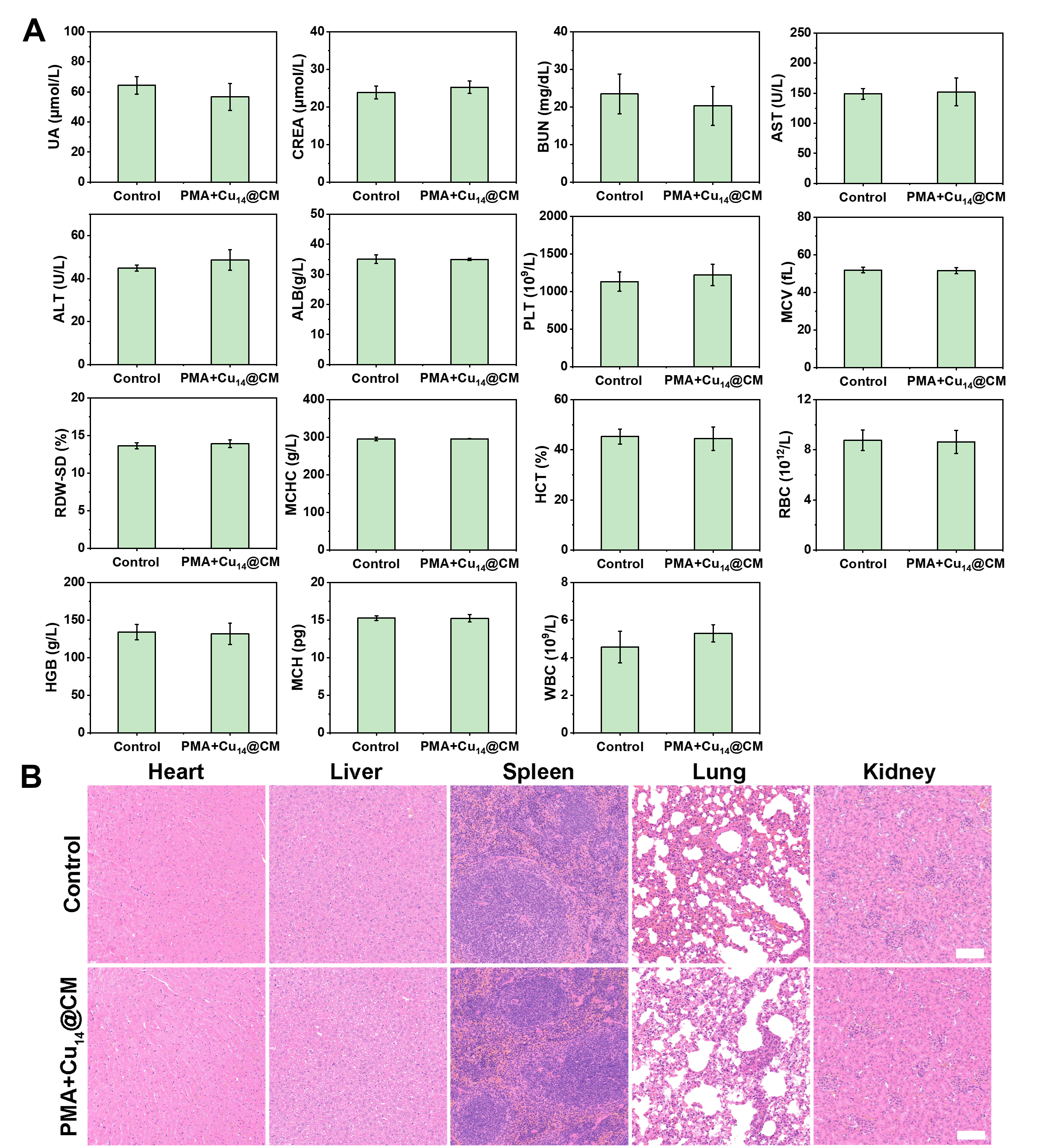


**Figure S47.** Long-term biocompatibility assessment. (A) Blood biochemical and hematological analysis in mice following various treatments. UA, urea; CREA, creatinine; BUN, blood urea nitrogen; AST, aspartate transferase; ALT, alanine transferase; ALB, alburnin; PLT, platelet count; MCV, mean corpuscular volume; RDW-SD, red blood cell distribution width; MCHC, mean corpuscular hemoglobin concentration; HCT, hematocrit; RBC, red blood cell count; HGB, hemoglobin; MCH, mean corpuscular hemoglobin content; WBC, white blood cell count. Data are presented as mean ± s.d. from 3 independent biological replicates. (B) H&E staining images of major organs from mice treated with PBS or PMA+Cu_14_@CM. Scale bar, 100 μm.


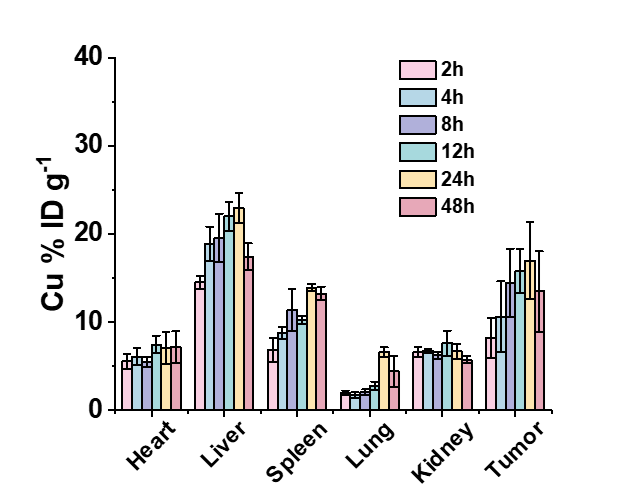


**Figure S48.** Biodistribution of Cu_14_@CM in orthotopic 4T1 tumor-bearing mice at different time points following intravenous injection. Data are presented as mean ± s.d. from 3 independent biological replicates.

[1] Y.L. Li, J. Wang, P. Luo, X.H. Ma, X.Y. Dong, Z.Y. Wang, C.X. Du, S.Q. Zang, T.C.W. Mak, Cu_14_ Cluster with Partial Cu(0) Character: Difference in Electronic Structure from Isostructural Silver Analog, *Advanced Science* 6, no. 18 (2019): 1900833. https://doi.org/10.1002/advs.201900833.
